# The Gearbox of the Bacterial Flagellar Motor Switch

**DOI:** 10.1016/j.str.2016.05.012

**Published:** 2016-07-06

**Authors:** Alessandro Pandini, Faruck Morcos, Shahid Khan

**Affiliations:** 1 Department of Computer Science and Synthetic Biology Theme, Brunel University London, Uxbridge UB8 3PH, UK; 2 Computational Cell and Molecular Biology, The Francis Crick Institute, London NW1 1AT, UK; 3 Department of Biological Sciences, University of Texas at Dallas, Richardson, TX 75080, USA; 4 Molecular Biology Consortium, Lawrence Berkeley National Laboratory, Berkeley, CA 94720, USA

## Abstract

Switching of flagellar motor rotation sense dictates bacterial chemotaxis. Multi-subunit FliM-FliG rotor rings couple signal protein binding in FliM with reversal of a distant FliG C-terminal (FliG_C_) helix involved in stator contacts. Subunit dynamics were examined in conformer ensembles generated by molecular simulations from the X-ray structures. Principal component analysis extracted collective motions. Interfacial loop immobilization by complex formation coupled elastic fluctuations of the FliM middle (FliM_M_) and FliG middle (FliG_M_) domains. Coevolved mutations captured interfacial dynamics as well as contacts. FliG_M_ rotation was amplified via two central hinges to the FliG_C_ helix. Intrinsic flexibility, reported by the FliG_MC_ ensembles, reconciled conformers with opposite FliG_C_ helix orientations. FliG domain stacking deformed the inter-domain linker and reduced flexibility; but conformational changes were not triggered by engineered linker deletions that cause a rotation-locked phenotype. These facts suggest that binary rotation states arise from conformational selection by stacking interactions.

## Highlights

• Switch complex exploits differential subunit stiffness for mechanical amplification
• Distinct rotor protein X-ray structures generate overlapping conformer ensembles
• Stacking constraints on a flexible helix linker could select diverse rotation states
• Non-contact elastic couplings at the subunit interface in the complex have coevolved

## Graphical Abstract

**Figure:**
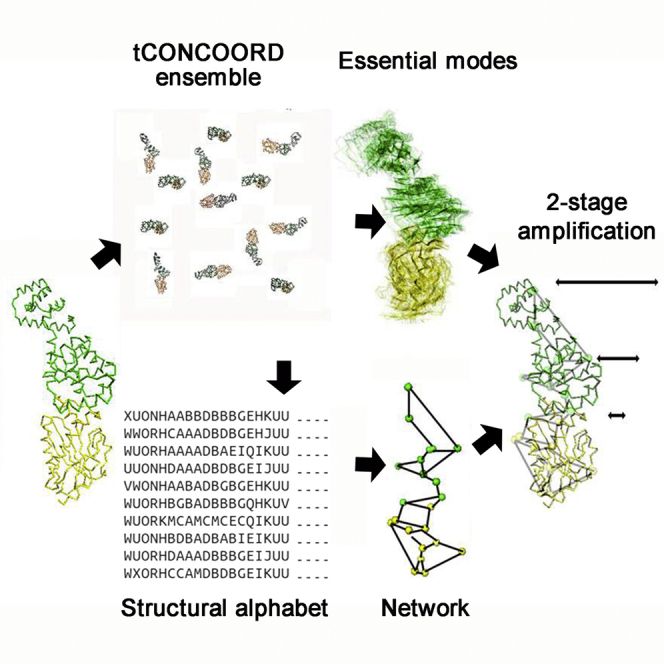

## Teaser

Pandini et al. analyze X-ray structures to chart mechanical signal transmission in a flagellar motor switch complex. Conformational ensembles reveal an evolutionarily conserved interface, two-stage amplification of elastic fluctuations, and conformational selection to explain the fast switching kinetics and provide insight into the allosteric mechanism.

## Introduction

The switching of bacterial flagellar rotation provides a remarkable example of a cooperative switch in a large, biomolecular assembly (Bray and Duke, 2004). The assembly, the rotor of the bacterial flagellar motor within the basal body, is composed of about 200 subunits of the component proteins (FliG, FliM, and FliN). These proteins attach to the membrane scaffold formed by FliF subunits forming the C and MS rings (Lux et al., 2000). The interaction of membrane-embedded Mot stator complexes with FliG subunits couples proton transfer to torque generation (Zhou et al., 1998). Chemotactic stimuli change the association of the CheY signal protein with the distal FliM_NC_FliN C ring (Dyer et al., 2009, Sarkar et al., 2010). Coupled conformational transitions in FliM_M_ (Sircar et al., 2015) trigger large displacements of a distant α helix in FliG, involved in stator contacts (Lam et al., 2012, Paul et al., 2011), henceforth designated toque helix (TH). The chemotactic motor output is a changed clockwise (CW)/counter-clockwise (CCW) rotation bias. CW and CCW intervals have second lifetimes, but switch within milliseconds, mostly with no detectible change in rotation speed (Bai et al., 2013, Lele and Berg, 2015). Absence of intermediate states implies cooperative switching of the multiple subunits (Ma et al., 2012). Activated CheY elicits an “ultra-sensitive” (H = 21) change in CW/CCW bias (Yuan and Berg, 2013), but its binding to motors in situ or rotor assemblies in vitro is not cooperative (Sagi et al., 2003, Sourjik and Berg, 2002). Thus, cooperativity must arise from mechanical amplification within the rotor.

**Figure 1:**
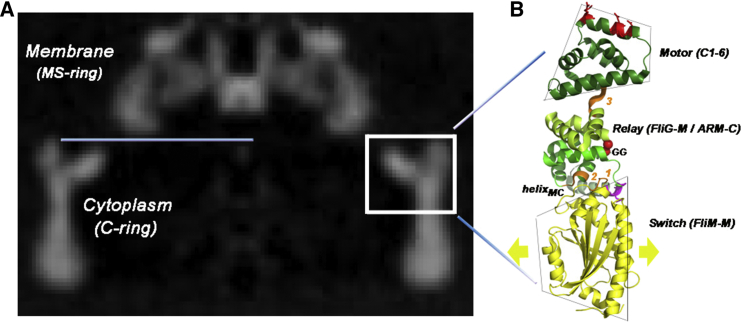
The *Salmonella* Basal Body MSC Ring and the *T. maritima* Proximal Switch Complex (A) A cross-section through the *Salmonella* flagellar basal body electron microscopy reconstruction (Thomas et al., 2006) showing transmembrane MS ring and the cytoplasmic C ring. Blue line marks membrane cytoplasm boundary. Box marks the surmised location of FliG and associated FliM_M_. The FliG ring interacts with transmembrane Mot stator complexes. FliM_M_ reports CheY binding to FliG and adjacent FliM subunits (gold arrows). (B) The atomic structure (PDB: 4FHR) of the *T. maritima* complex: FliM_M_ (gold), FliG_M_ (green), FliGc ARM-C (olive), C1-6 (dark green), MFVF motif (orange), and TH (red side chains). Deletion of three *Salmonella* residues homologous to *T. maritima* PEV (magenta) produces a CW biased phenotype. See also Figure S1 for secondary structures and the contact interface.

Genetic and biochemical studies on the enteric bacteria *Escherichia coli* and *Salmonella enterica* serovar (“*Salmonella*”) provide the paradigm for energization and switching of motor rotation. Non-motile, flagellate (*mot*) and non-chemotactic (*che*) mutations are found in all three proteins. The TH is targeted by *mot* mutations (Lloyd and Blair, 1997). The majority of *che* mutations are in FliM (Magariyama et al., 1990), FliG helix_MC_, and GG loop (Figure 2 of Brown et al., 2002). Other conserved loop motifs (GGXG in FliM_M_, EHPQ in FliG_M_, MFXF in FliG_C_ (letter = conserved residue; X = variable residue), are also targeted by *che* mutations. Figure 1 shows the surmised location of one of ∼35 copies of the most complete X-ray structure (*T. maritima* FliM_M_FliG_MC_ [Vartanian et al., 2012]) in the *Salmonella* basal body. FliM_M_, a dedicated switch module, is a pseudo-symmetric α/β/α sandwich with CW and CCW *che* mutations localized to distinct surface patches (Park et al., 2006). FliG_MC_ has multiple armadillo (ARM) domains; an architectural design that characterizes the entire protein (Lee et al., 2010). The FliG_C_ C-terminal six-helix bundle (C1-6) contains the TH, forming the motor module.

Here we study the X-ray structures (noted by PDB IDs) to understand the conformational coupling between the switch and motor modules. The available FliG and FliM X-ray structure library is marked by conformational heterogeneity, exemplified by two FliG_MC_ *Helicobacter pylori* structures with opposite (180°) FliG_C_ C1-6 orientations relative to its N-terminal ARM-C (Lam et al., 2012), that has engendered a lively debate (Stock et al., 2012). The heterogeneity could arise because component subunits have discrete states trapped in different minima in the energy landscape; analogous to the open and closed states of sugar binding proteins (Morcos et al., 2013). Alternatively, it could be due to intrinsic flexibility, with the two rotation states generated by conformational selection as found for binding of ADP to the F_0_F_1_ ATP synthase (Czub and Grubmuller, 2014). We used tCONCOORD to discriminate between these alternatives. tCONCOORD generates atomic-detail conformational ensembles from a single structure based on distance constraints (de Groot et al., 1997). Detection of labile hydrogen bonds facilitates conformational transitions (Fernandez and Scheraga, 2003, Seeliger et al., 2007). Collective motions were extracted from principal component analysis (PCA) (Amadei et al., 1993) of the ensembles. The dynamics of successive four-residue fragments in conformers encoded as a set of strings with a structural alphabet (SA) (Pandini et al., 2010) unveiled the local motions generating collective modes. Network analysis related interfacial dynamics and coevolution; an important issue for protein machines being addressed by various groups (Morcos et al., 2013, Sfriso et al., 2016, Sutto et al., 2015). Finally, we engineered a three-residue FliG linker helix_MC_ deletion in all structures to assess whether it triggers conversion to the stacked conformation observed for the deletion protein X-ray structure.

The FliM_M_ structure ensembles reveal a stiff domain that fluctuates between two states. The FliG_MC_ structure ensembles, irrespective of species, sample a broad conformer space that is constrained by FliG ARM domain stacking. Residue coevolution identifies both FliG_M_FliG_MC_ interfacial contact and elastic couplings. Complex formation couples FliM_M_ fluctuations to FlG_M_ rotation, amplified via two central hinges to a large angular reorientation of FliG_C_ C1-6. The design allows rapid reorientation of FliG_C_ C1-6 upon altered tilt of the more rigid FliM_M_ ring within the basal body. Helix_MC_ architecture is too pliable for deletions within it to trigger ARM domain stacking. Instead, the stacking could select alternative rotation states from a broad conformational spectrum.

## Results

Our analysis of the tCONCOORD conformational ensembles had two stages. First, we examined the ensemble from the FliM_M_FliG_MC_ complex. Anharmonic collective motions were identified by PCA of residue C_α_ position fluctuations and the principal components (PCs) mapped onto the structure. Conformational dynamics of SA-encoded fragments were correlated with the PC motions and each other for characterization of the mechanical network, its relation to interface coevolution, and perturbation by engineered CW-locked deletion mimics. Second, we applied the methodology to the complete structure library of the component FliM_M_ domains, FliG_MC_ and FliM_M_FliG_M_ complexes. The comparative analysis assessed the effects of complex formation on the individual components, determined a common mechanical design, and evaluated the species-dependent contribution to the variability.

### The *T. maritima* FliM_M_FliG_MC_ Conformer Ensemble Records Large FliG_c_ Motor Domain Movements

**Figure 2:**
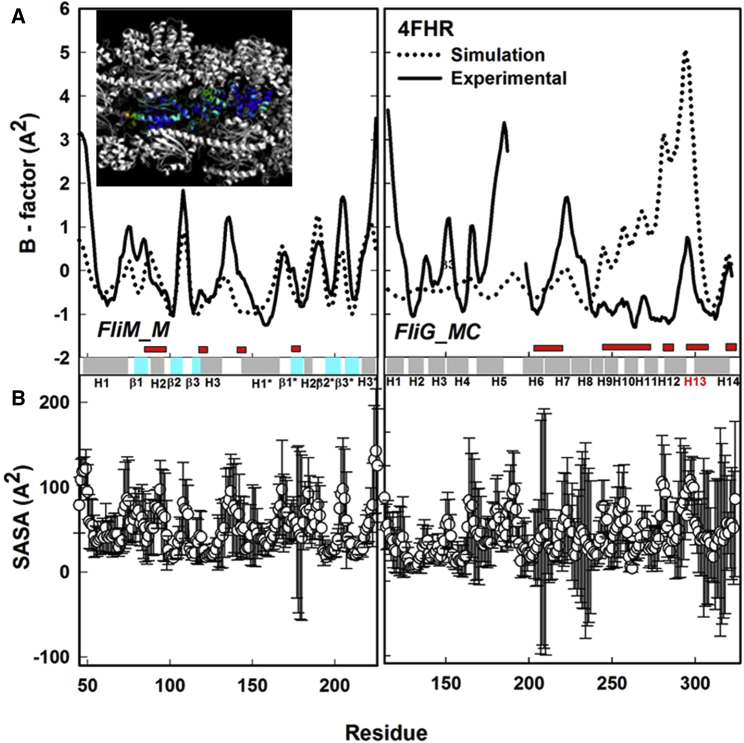
Residue Fluctuations in FliM_M_FliG_MC_ (A) Experimental B-factor values (solid line) compared with simulated values (dotted line). The B factors were normalized. B = ((B_res_ − B_mean_)/σ_B_), where B_mean_ and σ_B_ are mean and SD, respectively, of the simulated B-factor (B_res_) distribution. Parameters in later figures are normalized similarly. Red bars denote residues in contact with neighboring complexes in the crystal. FliM_M_ P_corr_ = 0.63. FliG_MC_ P_corr_ = (−0.2 [overall]; 0 [non-contact N-terminal]). Inset: PDB: 4FHR unit cell (B factor high = orange; intermediate = green/cyan); low = blue) and neighboring complexes (white). H13 = TH. (B) Residue SASA (mean [open circles] ± σ). Horizontal bar shows secondary structure elements: α helices (gray), β sheet (cyan), loops (white).

Computed residue temperature factors (B factors) for the FliM_M_FliG_MC_ ensemble were compared with experimental values (Figure 2A). The simulated FliM_M_ B-factor profile was in reasonable agreement with the crystallographic factors. In contrast, the match was poor for FliG_MC_. The dominant peak in the simulated profile, at the TH, was damped in the experimental profile. The most prominent peak in the experimental profile bordered the missing seven-residue segment (V_188_SRTFSK_194_) adjacent to the G_196_G_197_, grafted in from another *T. maritima* structure (PDB: 1LKV). The second peak was centered at ARM-C E_223_. Downweighted, low-amplitude peaks were obtained at these positions in the simulated profile. Solvent-accessible surface area (SASA) variations within the ensemble (Figure 2B) identified the β1^∗^/H2^∗^ loop (E180-P184) as the most variable FliM_M_ segment. In FliG_MC_, the high B-factor segments (H6/H7 GG, H8/H9 MXVF, and TH N-terminal loops) had the most variable SASA.

### PCA Identifies TH Displacements as the Principal Collective Motion

**Figure 3:**
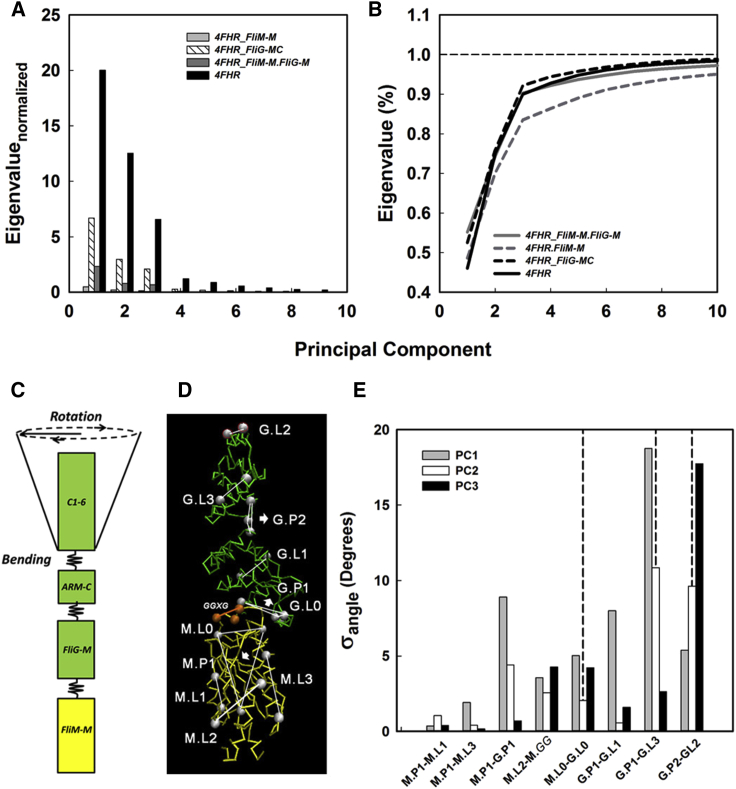
PCA of the FliM_M_FliG_MC_ Complex (A) Eigenvalues for the first ten PCs of the ensembles normalized by the sum of the FliM_M_ (gray) eigenvalues. (B) Cumulative spectra (lines) show the anisotropy of FliM_M_ motions is increased in complex with FliG_M_ (dark gray line) or FliM_M_FliG_MC_ (black line) than when alone (dashed gray line). The anisotropy of FliM_MC_ (dashed black line) motions is not affected by complex formation. (C) Schematic of the complex as a segmented rod with intervening hinges. FliM_M_ (gold), FliG_MC_ (green). (D) Vectors (white lines) used for measurement of hinge and interfacial motions. White spheres mark C_α_ atoms of connected residues. White arrows show perpendiculars to the chosen planes (triangles). Orange vector (line) marks GGPG motif (spheres). (E) First three PC amplitudes between vector pairs measured as the σ of the difference angle distributions. Bending = MP1.ML1, MP1.ML3, MP1.GP1, GP1.GL1, GP1.GL3. Rotation = ML2.MGG, ML0.GL0, GP2.GL2. Dashed line separates FliM_M_ and FliG_MC_ pairs. Vector labels are as in (D).

The isotropic motions of an ideal molten globule have a flat PC spectrum with equal amplitude eigenvalues. Secondary structures create hinges and shear planes that coordinate collective, anisotropic movements to generate dominant PC modes. The complete PC spectrum measures overall flexibility. Domains were isolated from the complex in silico to assess the effects of complex formation. The relative PC amplitudes were normalized with respect to the summed amplitude of the FliM_M_ eigenvalue spectrum (Figure 3A) to show that the intrinsic flexibility of FliG_MC_ was substantially more than that of either the smaller FliM_M_ or the larger FliM_M_FliG_M_ complex. The variance of the ensemble was largely (∼90%) captured by the first three PCs. We plotted cumulative amplitudes to better determine differences in anisotropy (Figure 3B). The FliM_M_ PC spectrum becomes more anisotropic upon complex formation with either FliM_M_ or FliG_MC_. In contrast, FliG_MC_ was not affected by complex formation.

We constructed a mechanical analog to physically map the PC amplitudes onto the structure (Figure 3C). The FliM_M_FliG_MC_ complex was represented as a segmented beam. The FliG GG and MXVF loops constituted flexible hinges consistent with the SASA profile, in addition to the subunit interface. Hinge motions were most simply deconvolved into bending and rotary components as measured for another segmented protein, the myosin rod (Highsmith et al., 1982). We marked line vectors within the structure (Figure 3D) and recorded ensemble distributions of angle fluctuations between vector pairs to refine the mechanical model. The SDs (σ) of the angle distributions (Figure 3E) showed that the subunit interface and inter-ARM loops were more flexible relative to other parts of the protein, consistent with the initial model. However, the GGPG loop rotary twist (ML2-MGG) was prominent in FliM_M_ motions. FliG_M_, mechanically coupled to FliM_M_ bends and rotates at the interface (ML0-GL0). C1-6 bending and rotary motions (GL2-GP2) relative to FliG_M_ (GL1-GP2) are amplified from the interface motions. The overall amplification is 3.8-fold for PC2 and 4.2-fold for PC3. The PC contributions to the TH (GL2-GP2) σ were ±5.4° (PC1)/±9.6° (PC2)/±17.7° (PC3). PC1 (Movie S1) predominantly recorded bending motions at the interface and the MFVF motif, and PC3 (Movie S2) the rotation of FliG C1-6 relative to FliM_M_.

### The Elastic Compliance in the Coupling between FliM_M_ and FliG C1-6

**Figure 4:**
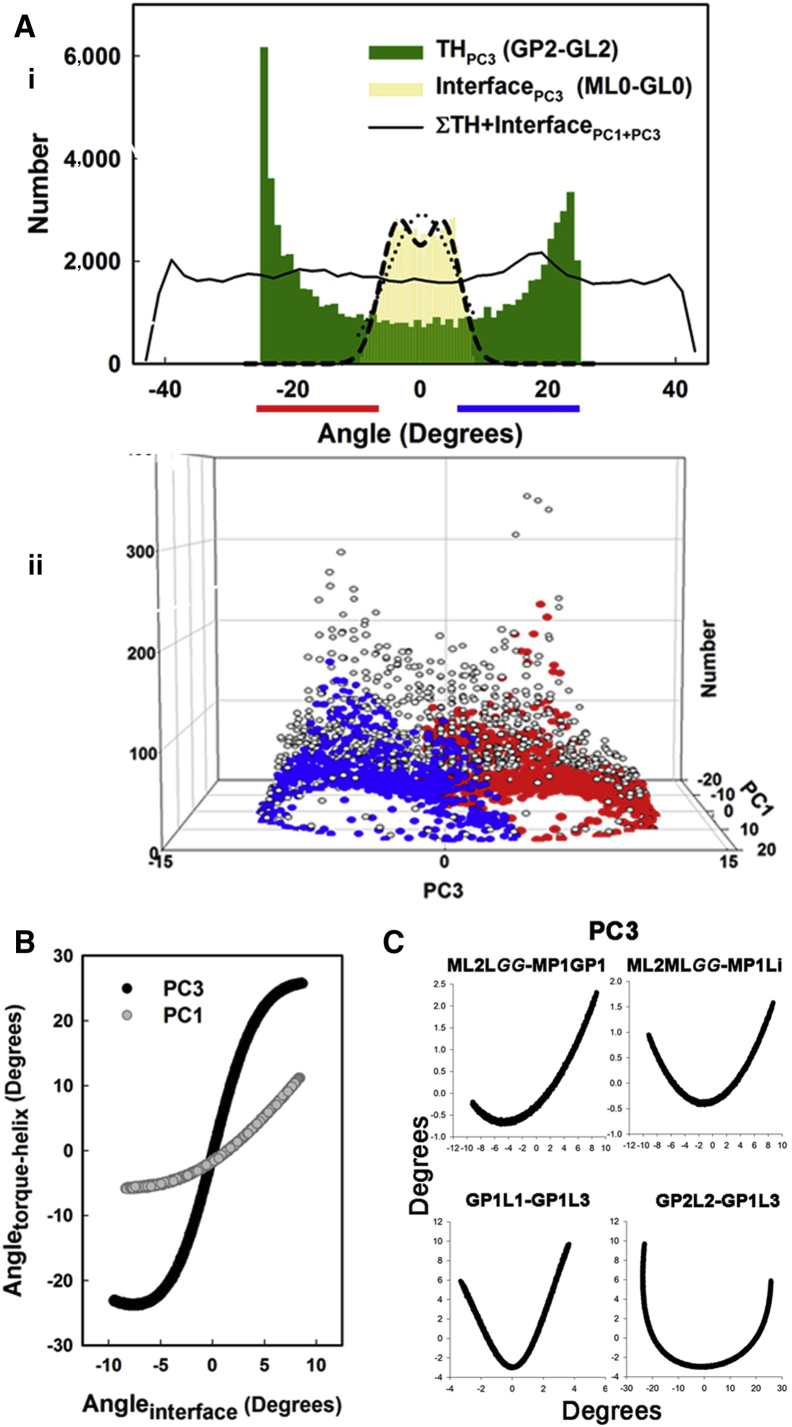
Two-Stage Rotary Amplification (A) (i) PC3 rotation angle distributions. Horizontal bars (red [<5°]/blue symbols [>5°]) denote subpopulations of the TH (GL2-GP2) distribution. Fits to the FliMFliG interface (ML0-GL0) distribution are single Gaussian (y = a^∗^exp(−0.5((x − x0)/b)^2^)), where a = 2,904, b = 5.48, x0 = 0.2 (dotted line) and double Gaussian (a^∗^exp(−0.5((x − x0)/b)^2^)) + (a^∗^exp(−0.5((x + x0)/b)^2^)), where a = 2,701, b = 2.72, x0 = 3.55 (dashed line). Combined PC1 + PC3 angle distribution (σ = ±13.0°) for both vector pairs (line). (ii) The subpopulations of the TH distribution partition to opposing ends of the PC1PC3 plot, showing that conformer spread (open symbols/gray edges) tracks TH motions. (B) The coupling between interface and TH motions. (C) Elastic coupling (PC3 rotation) between other elements in the complex. Vectors are as in Figure 3C. Mean orientations (0°) in (B) and (C) are for the ensemble-averaged structure.

Two angle distributions for the PC motions were examined in detail. The distribution for the TH PC3 rotation relative to the MFVF hinge is bimodal with the ends more populated than the center (Figure 4A). A two-Gaussian (R^2^ = 0.94) fit is also better than a single Gaussian (R^2^ = 0.78) for angular displacements measured across the subunit interface. The torsional stiffness estimated from the interface rotation is 740 pNnm (one state) to 1,500 pNnm (two states). C1-6 rotation determined the conformer spread as seen from projection of its angular distribution on the PC1PC3 plane. Similar results were obtained for projection onto the PC2PC3 plane.

The composite PC1 + PC3 rotation at the interface and MFVF hinge had a flat angular distribution with increased spread. The flat distribution resulted from summation of two PCs with different relations for the TH-interface motions (Figure 4B). For PC1, the relation between the interface and TH rotation amplitudes is monotonic. For PC3, there are end states where TH orientation is insensitive to interface motions, separated by a linear (8 ± 1) response. Both relations are distinct from relations between inter-domain bending motions that have a parabolic form consistent with motion in an elastic potential well (Figure 4C). Specifically, interface rotation of the GGPG loop relative to the FliM_M_ H1/H2 long axis is constrained by H2 displacement from its favored orientation relative to β1-β3 sheets. Rotation of FliG C1-6_c_ around the MFVF loop is constrained by its bending relative to FliG_M_. These elastic couplings preserve the protein fold. The C1-6 rotational flexibility at the MFVF hinge (GP2-GL2) from the complete PC has σ = ±28°.

### Detailed Analysis of Hinge Elements

**Figure 5:**
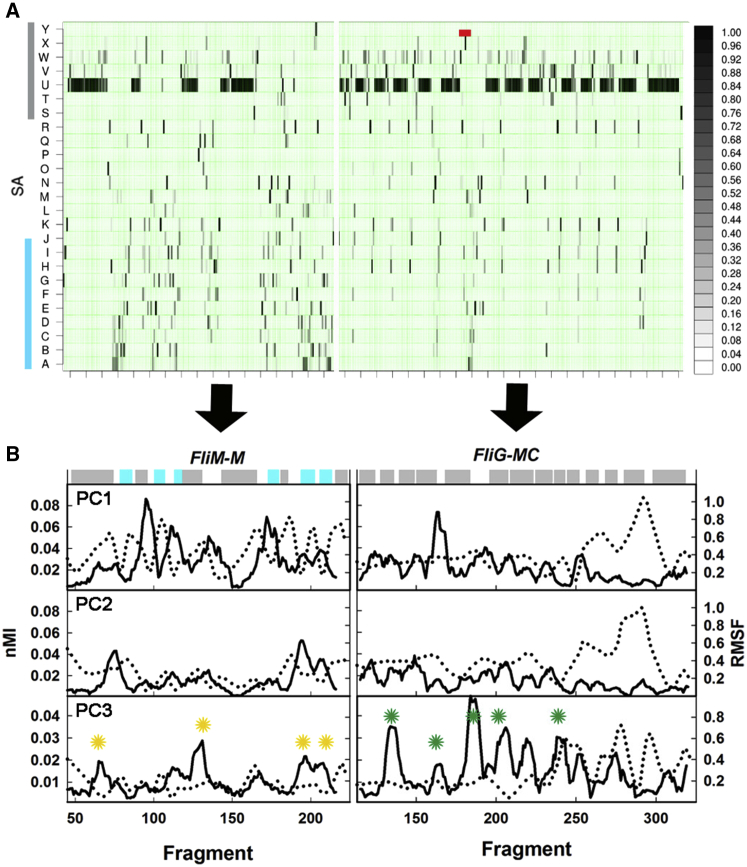
Hinge Elements for the PC Motions (A) SA representation of ensemble fluctuations. SA letters readout of secondary structure (α helix [gray bar], β sheet [blue bar]) as detailed (Figure S2). Red bar denotes grafted PDB: 1LKV helix_MC_ segment. Grayscale bar (black, high; white, low) denotes populated ensemble fraction. (B) Superimposed nMI_PC_ (black lines) and RMSF (dotted lines) profiles for PDB: 4FHR. Horizontal bar, colors as in (A), shows secondary structure profile. Peaks that represent hinges for the PC3 rotation in FliM_M_ (yellow asterisks) and FliG_MC_ (green asterisks) are marked. For FliM_M_ RMSF-nMI_PC1_ P_corr_ = −0.17, RMSF-nMI_PC3_ P_corr_ = −0.2, nMI_PC1_-nMI_PC3_ P_corr_ = 0.27. For FliG_MC_, RMSF-nMI_PC1_ P_corr_ = −0.36, RMSF-nMI_PC3_ P_corr_ = −0.21, nMI_PC1_-nMI_PC3_ P_corr_ = 0.21. See also Figure S2.

SA-encoded fragment motions (Figure S2) characterized local fluctuations (Figure 5A). Fragments from secondary structures in the crystal sampled conformations that preserved type throughout the ensemble. Short loops sampled loop-specific conformations; but long loops (e.g., GGPG loop), also sampled β-sheet conformations. The helix_MC_ segment grafted from PDB: 1LKV sampled loop and β-sheet, rather than α-helix, conformations.

Local fragment fluctuations were correlated with trajectory displacements along the PCs (Figure 5B). Hinges, defined as segments with high nMI_PC_ contribution, included both static (low root-mean-square fluctuation [RMSF]) and dynamic (high RMSF) elements. Within FliM_M_, the prominent static hinges were loops between H2/β2, H1^∗^/β1^∗^ (PC1), H1/β1, H2^∗^/β2^∗^ (PC2), and H2^∗^/β2^∗^, β2^∗^/β3^∗^ (PC3). For all three PCs, the long FliM_M_ GGPG loop was a dynamic hinge. The FliM_M_ HI long helix central segment, enriched in polar residues and thus susceptible to hydrolysis, was the second dynamic hinge (PC3). Within FliG_MC_, the N-terminal helix_MC_ loop was a hinge for PC1 and PC3; while the EHPQ, GG, and MFVF motif loops formed additional PC3 hinges. For all three PCs, the RMSF profile peaked at or adjacent to the TH, accounting for the B-factor profile (Figure 2A).

In conclusion, the premise for the segmented beam model is validated, but supplemented with knowledge of the inherent elasticity of the FliM_M_ and FliG_M_ segments.

### The Mechanical Network between FliM and FliG

**Figure 6:**
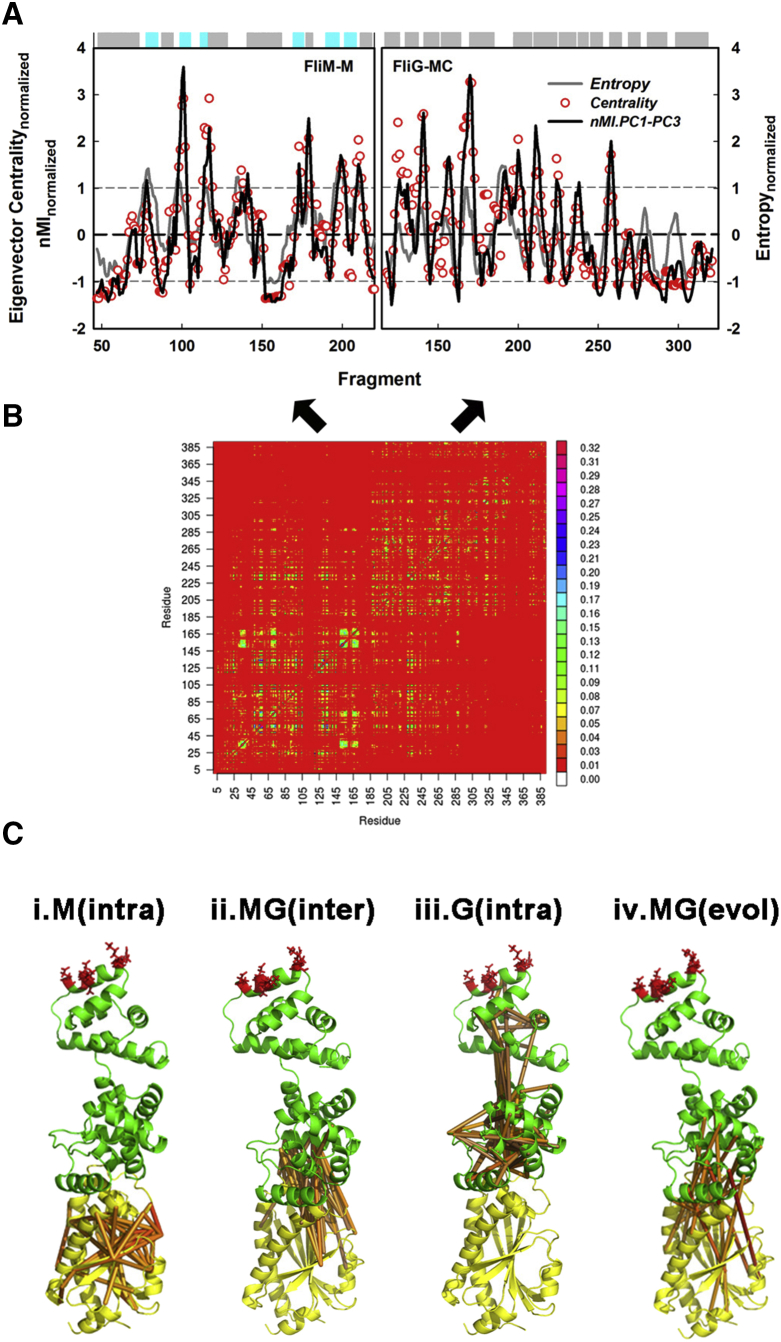
Network Analysis of Local Correlations (A) The eigenvector centrality tracks the averaged nMI_PC_ profile for the PC1-PC3 motions (FliM_M_ P_corr_ = 0.96; FliG_MC_ P_corr_ = 0.90). Fragment centrality and entropy are correlated (FliM_M_ P_corr_ = 0.44; FliG_MC_ P_corr_ = 0.13). Horizontal bar shows secondary structure profile as in Figure 2B. (B) The covariance matrix. Side bar shows nMI_local_ color scale. (C) The top (nMI > 0.15; red [high] − bluish brown [low]) (i) FliM_M_ intra-domain, (ii) FliM_M_FliG_M_ inter-subunit, and (iii) FliG_MC_ intra-domain correlations; and (iv) top coevolved inter-subunit couplings mapped onto the PDB: 4FHR structure, color-coded as in Figure 1. See also Figures S3 and S4.

We constructed FliM_M_ and FliG_MC_ centrality profiles from the covariance matrix of the encoded fragment correlations to measure the contribution of each fragment to the network of local motions (Figures 6A and 6B). The entropy profile identified flexible loops. The FliM_M_ and FliG_M_ loops form a distributed hinge system of network nodes in the composite profile. The remaining two nodes localized to the MFVF motif and the central C1-6 helix. The C1-6 loops inter-helix loops did not influence the network. The profile peaks represented the major nodes: nine for FliM_M_ and ten for FliG_MC_. The profiles superimposed with the composite nMI_PC_ profile for the PC1-PC3 motions (P_corr_ > 0.9). Thus, the PC1-PC3 motions are the dominant output of the mechanical network.

Three helix_MC_ residues (PEV) homologous to the CW-locked *Salmonella* deletion (PAA) form its N terminus close to the interface. Their deletion reduced FliG_M_ intra-domain contacts as well as contacts with FliM_M_ (Figure S4). However, long-range couplings between FliM_M_ and loops adjacent to the FliG TH persisted and collective motions were largely unaltered (Movie S3), highlighting the robust nature of the distributed system.

Structural maps of the top nMI_local_ correlations (Figure 6C) revealed the mechanical relay between FliM_M_ and FliG_C_. FliM_M_, with its β-sheet center as pivot, connects to FliG_M_ core helices H2 and H4. Comparison of the FliMFliG interface residue coevolution and dynamics (Figure S3) showed that, in addition to interface contacts, nodes of the coevolved network overlap/flank long-range dynamic network nodes. The overlap is evident in the structural maps of the communication pathways. However, coevolution only reports some β-sheet motions important for interface dynamics, for reasons not presently understood. FliG_M_ also has a dense network built around its core helices with sparse connectivity to C1-6. The network centrality and spatial architecture are consistent with the idea that mechanical transmission may be conceptually divided into two stages: mechanical coupling at the interface that transmits fluctuations of the stiff FliM_M_ domain to FliG_M_; with subsequent transmission via hinge motions to effect C1-6 rotation.

### Comparative Analysis of Component Structures

X-Ray structures of component proteins and partial complexes were superimposed with the reference PDB: 4FHR structure on a domain-by-domain basis based on common residue positions. Superposition of these static structures did not reveal differences between the species (Figure S5). We proceeded with analysis of the conformer ensembles.

### The FliM_M_ GPGG Loop Is Immobilized by Complex Formation

**Figure 7:**
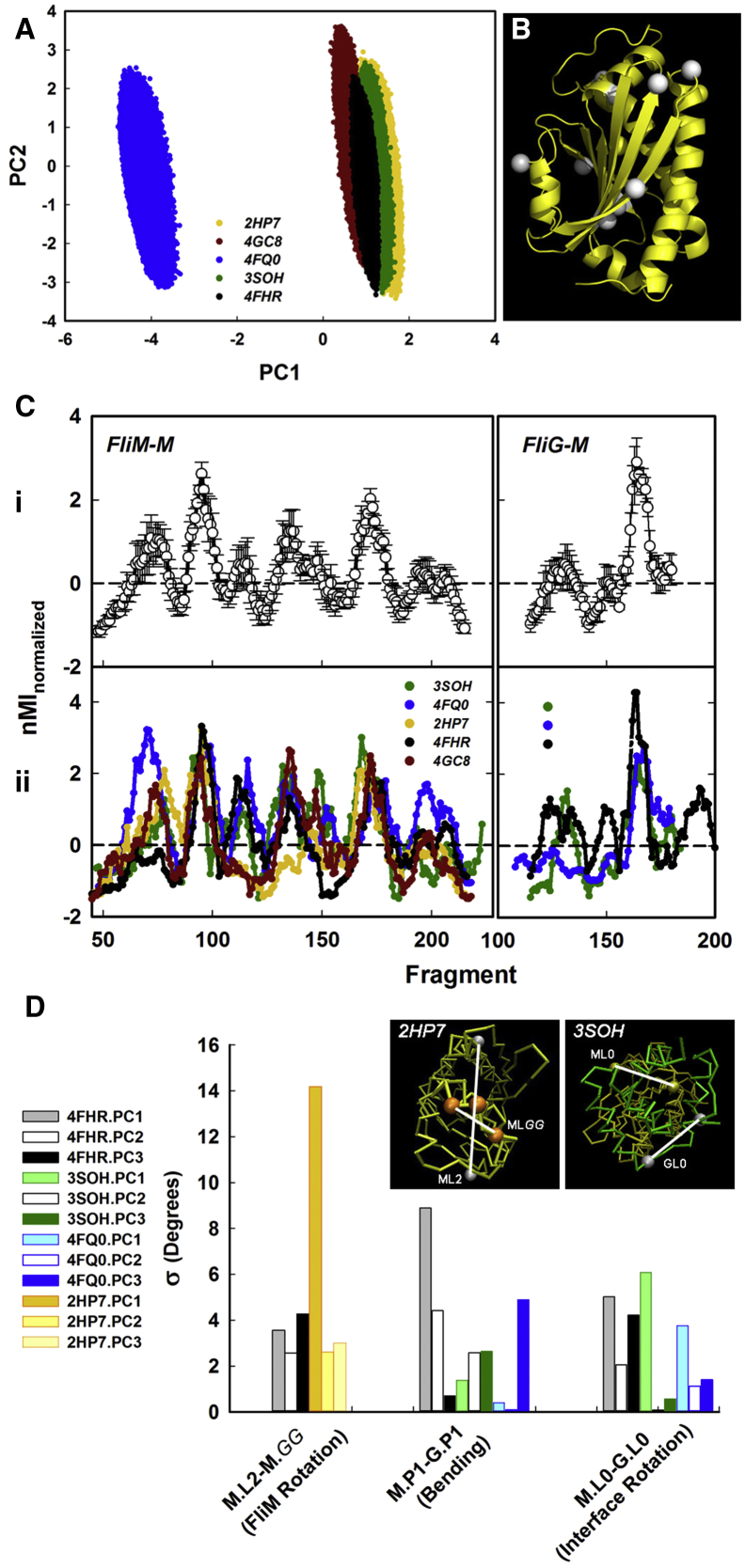
Comparative Dynamics of FliM Structures (A) PC1PC2 plots of FliM_M_ ensembles from the monomers (PDB: 2HP7, 4GC8) and complexes (PDB: 3SOH, 4FQ0, 4FHR). The PC1-PC3 conformer spread (σ) was 0.628 ± 0.002 (PDB: 2HP7) and 0.578 ± 0.002 (PDB: 4GC8); 0.448 ± 0.002 (PDB: 3SOH), 0.578 ± 0.002 (PDB: 4FQ0), and 0.582 ± 0.002 (PDB: 4FHR). (B) Dynamic network nodes (white spheres) mapped onto the PDB: 4FHR FliM_M_ backbone. (C) Hinge detection from the fragment nMI_PC_, contribution. (i) Averaged nMI_PC1_ profile (±σ). (ii) Individual nMI_PC1_ profiles. P_corr_ values (PDB: 4FHR reference) were 0.43 (PDB: 2HP7), 0.52 (PDB: 4GC8), 0.44 (PDB: 3SOH), and 0.57 (PDB: 4FQ0). (D) First three PC distribution σ of the bending and rotary motions of PDB: 4FHR and FliM_M_FliG_M_ complexes measured with vector pairs as defined in Figure 3D. Insets: snapshots from Movie S4 (PDB: 2HP7) and Movie S5 (PDB: 3SOH) documenting PC1 and complete PC motions. See also Figure S5.

The PC1PC2 plots for the FliM_M_ monomers superimposed with the plot for PDB: 4FHR (Figure 7A). The FliM_M_ plot of the *H. pylori* FliM_M_FliG_M_ complex (PDB: 4FQ0) was displaced, albeit of similar form, from the other plots. The PC1-PC3 σ of the ensembles ranged from 0.628 ± 0.002 (PDB: 2HP7) to 0.448 ± 0.002 (PDB: 3SOH). The overlap showed that species differences and subsequent rearrangements of the stiff domain upon complex formation were small.

We correlated fragment dynamics with the global PC motions as for the PDB: 4FHR FliM_M_ dynamic network (Figure 7B). The nMI_PC1_ profiles of the FliM_M_ monomers and the FliM_M_FliG_M_ complexes followed the PDB: 4FHR profile (Figure 5B) (average P_corr_ = 0.49 ± 0.03) (Figure 7C), with some differences. The FliM_M_ monomer profiles lacked the β2/β3 loop network node. In addition, the contribution of the central GGPG node was reduced in the *T. maritima* monomer (PDB: 2HP7). The dominant node for FliM_M_FliG_M_ profiles, as in PDB: 4FHR, was N-terminal helix_MC_ interfacial loop. In the *H. pylori* PDB: 4FQ0 profile the FliM_M_ N-terminal H1 and the C-terminal β2^∗^/β3^∗^ loop were more prominent, the FliG H1/H2 EHPQ motif loop less so. The PDB: 4FHR profile agreed more with the profiles of the complexes rather than the monomers.

Rotary twist of the GGPG loop relative to the central long axis was the principal (PC1) motion in the FliM_M_ monomers (*T. maritima* PDB: 2HP7 [σ = ±14°] [Movie S4], *H. pylori* PDB: 4GC8 [σ = ±11°]). These motions exceeded the combined PC1-PC3 PDB: 4FHR FliM_M_ motion. The interfacial rotation of FliG_M_ relative to FliM_M_ was the principal PC1 motion in FliM_M_FliG_M_ complexes (Movies S5 and S6); while bending dominated the PC2 and PC3 motions (Figure 7C). We conclude that species interface dynamics vary in degree, not strategy; with twist of the GGPG loop, the dominant intrinsic motion of FliM_M_, harnessed upon complex formation to drive FliG_M_ rotation. Other features of the dynamic FliM_M_ network are conserved across all ensembles.

### Two Hinges Determine FliG_MC_ Flexibility

**Figure 8:**
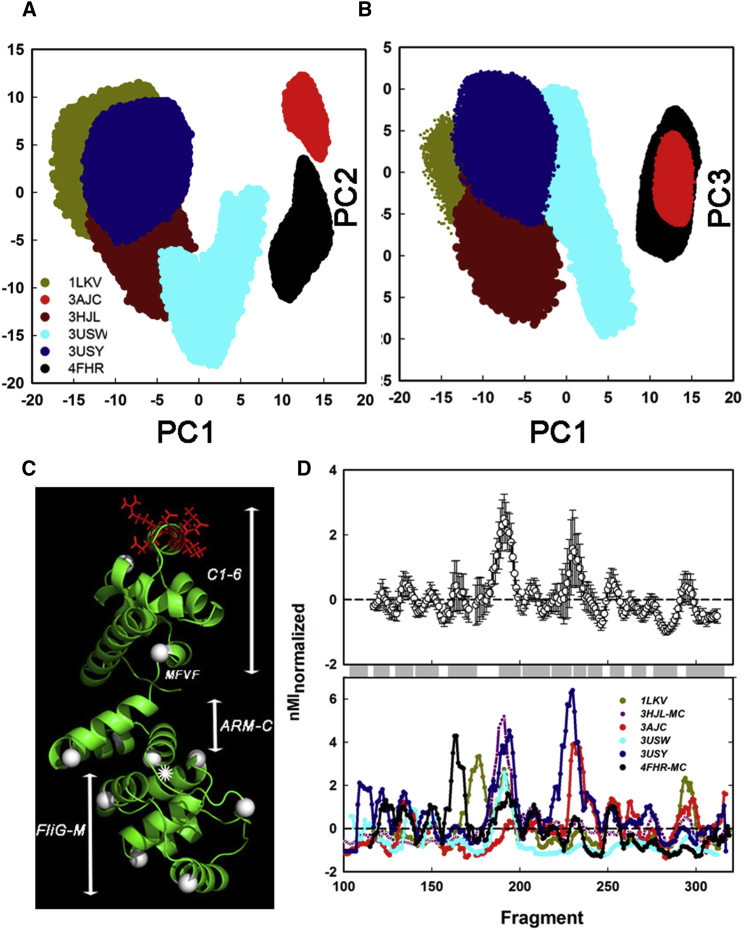
Comparative Dynamics of FliG_MC_ Structures (A) PC1PC2 plots. (B) PC1PC3 plots. (C) Dynamic network nodes (white spheres) mapped onto the PDB: 4FHR FliG_Mc_ backbone. GG pair (asterisk), TH (red side chains). (D) Averaged nMI_PC1_ profile (±σ) and the individual profiles. See also Figure S5.

The modulation of intrinsic FliG_MC_ flexibility by complex formation was determined similarly. The first three PCs were projected onto 2D planes (Figures 8A and 8B). All FliG_MC_ ensembles had greater spread (PC1-PC3 σ [nm]) than the FliM_M_ ensembles. The ensembles were resolved into two sets based on overlap and spread (PC1-PC3 σ). The overlapping *T. maritima* PDB: 1LKV (2.04 ± 0.006), *Aquifex aeolicus* PDB: 3HJL (2.09 ± 0.006), and *H. pylori* PDB: 3USY (2.655 ± 0.006) and 3USW (3.86 ± 0.014) ensembles formed one set separate from the *T. maritima* PDB: 3AJC (1.36 ± 0.004) and 4FHR (2.42 ± 0.008) ensembles. The latter structures have FliG_M_/ARM-C stacking interactions.

Correlations of the ensemble nMI_PC1_ profiles with PDB: 4FHR (Figure 8C) were worse (P_corr_ = 0.16 ± 0.06) than for FliM_M_, consistent with greater conformational variability. The nMI_PC1_ profiles of all ensembles were merged to detect common nodes (Figure 8D). The interface (EHPQ and N-terminal helix_MC_) loops were not prominent in the FliG_MC_ networks, showing that their PDB: 4FHR network centrality was due to complex formation. The GG and the MFVF loop formed the dominant nodes, with 3-fold greater amplitude than the next prominent node (TH C-terminal loop). This result extends the PDB: 4FHR segmented beam model to all FliG_MC_ structures.

### The Effects of Domain Stacking and FliM_M_ Complex Formation on FliG_MC_ Flexibility

**Figure 9:**
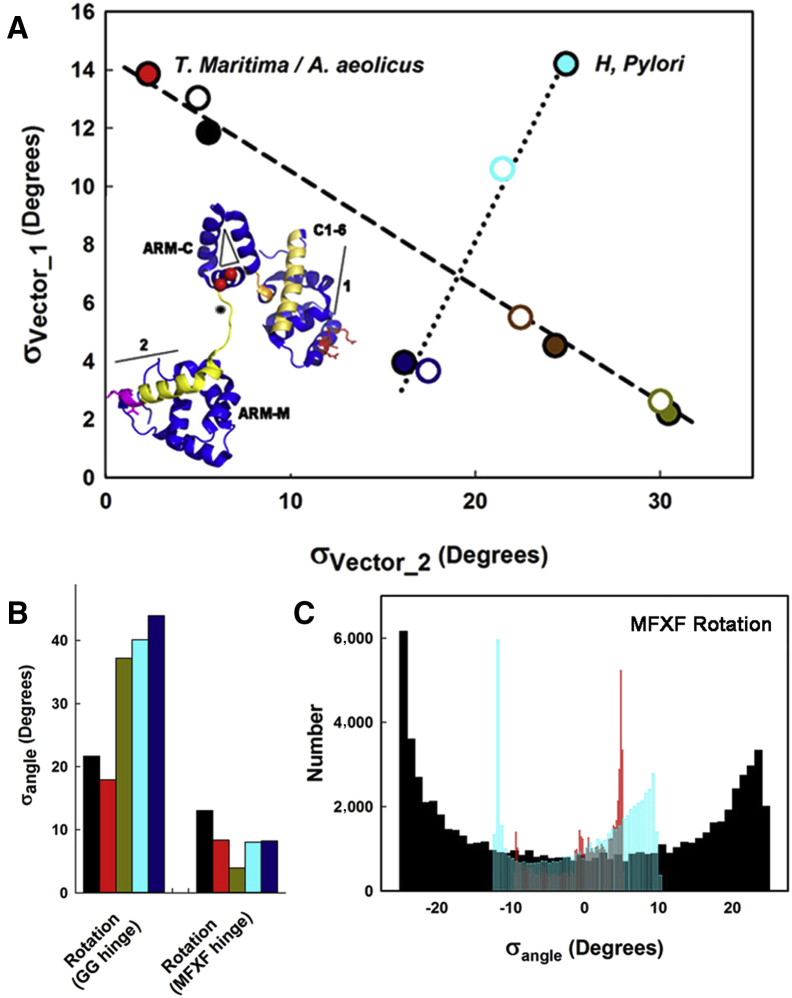
Central Hinge Dynamics (A) Complete PC hinge-bending amplitudes, recorded as difference angle distributions σ in FliG_M_ and C1-6 helices relative to ARM-C (inset). Open symbols denote engineered deletion structures (edge color = native structure). Inset: example PDB: 3USY C1-6 terminal helix (1, pale brown), FliG_M_ helix_MC_ (2, yellow), and ARM-C plane (triangle) (GG pair [red], PQV [magenta], and TH [red side chains]). (B) Complete PC hinge rotation amplitudes. GL1.GP2 (GG) and GP2.GL2 (MFXF) vector sets (defined in Figure 3C). (C) PC1 (PDB: 3AJC, 3USY) and PC3 (PDB: 4FHR) MFXF hinge rotation. Colors denote structure ensembles as in Figure 8A. See also Figures S6 and S7.

We reasoned that FliG_MC_ conformer ensembles are best compared by motions around the two central hinges. As for PDB: 4FHR, we generated ensembles from structures with engineered PEV or homologous deletions. Complete PC spectra recorded the bending (Figure 9A) and rotary (Figure 9B) flexibility of the two hinges. The hinge distributions formed two distinct relations. The *T. maritima*/*A. aeolicus* native and deletion FliG_MC_ structure ensembles formed one relation (R^2^ = 0.99) that spanned a large GG hinge range due to the presence of both unstacked and stacked structures. The structures with the stacking interaction had markedly reduced GG hinge flexibility, partly compensated by increased flexibility at the MFVF hinge. The *H. pylori* relation (R^2^ = 0.97) had a similar range for MFVF hinge-bending flexibility, but its reduction was coupled to decrease, not increase, at the GG hinge. The deletions reduced GG hinge-bending flexibility, as expected from the reduced helix_MC_ length, hence leverage.

Comparison of the two most divergent *T. maritima* and *H. pylori* X-ray crystal structures reveals that in both cases, ARM-C moves between coaxial and orthogonal orientations with respect to FliG_M_ (Figure S6). The coaxial ARM-C structures, *H. pylori* PDB: 3USW and *T. maritima* PDB: 3AJC, were most similar to PDB: 4FHR. TH displacements produced by rotational flexibility of the MFVF hinge were determined from the main PC modes. MFVF hinge rotation was the principal (PC1) motion for both structures (Movies S7 and S8). While the PDB: 3USW angle distribution has similar form to the PDB: 4FHR PC3 distribution, the PDB: 3AJC distribution is asymmetric. In both cases, the motions were restricted compared with the corresponding PDB: 4FHR rotation (Figure 9C). Therefore, the large PDB: 4FHR MFVF hinge rotation is not intrinsic to FliG_MC_, but a consequence of complex formation.

Local helix_MC_/GG hinge dynamics gave insight into the regulation of domain motions by this hinge and deletions within it (Figure S7). The dynamics are different for the unstacked versus stacked *T. maritima* conformations. The C-terminal half of this segmented hinge behaves as an unstructured loop element, rather than an α helix in the stacked (PDB: 3AJC, 4FHR) conformations, despite graft-in of seven missing residues from the unstacked (PDB: 1LKV) structure where these residues form an extended α helix. In contrast, helix_MC_ is more flexible in both *H. pylori* conformations due to a long C-terminal loop segment. The long loop eliminates the compensatory coupling between the two hinges seen for *T. maritima*. In contrast to *T. maritima*, the homologous PQV deletion will more severely reduce the shorter helix in the *H. pylori* serial N-terminal helix/C-terminal loop relay and, hence, torque transfer to the MFVF hinge in the coaxial conformation. In the orthogonal conformation the long C-terminal loop will determine hinge flexibility.

## Discussion

The analysis of the *T. maritima* FliG_M_Fli_MC_ complex revealed the following. (1) Large deviations in FliGc C1-6 residue positions were masked by inter-molecular crystal contacts. (2) Large C1-6 rotary and bending motions were the output of a two-stage amplification of FliM_M_ rotary twist fluctuations mediated by the FliG_MC_ GG and MFVF loops. (3) Interfacial loops coupled dynamics of the contacting domains while their internal loops preserved protein fold. (4) FliG_M_ and ARM-C loops formed a sparsely distributed network. (5) A CW-locked *Salmonella* deletion mimic weakens adjacent FliG_M_ couplings, but long-range couplings between FliM_M_ and the TH persist.

The analysis of the FliM and FliG structure library established that: (1) immobilization of the FliM_M_ GGPG loop upon complex formation generates coaxial rotation of FliG_M_ relative to FliM_M_ in *H. pylori* as well as *T. maritima*; (2) different FliG_MC_ conformations from these species show distinct relations between central hinge motions; (3) FliG_MC_ dynamic network architecture is minimally altered by CW-locked deletion mimics; and (4) the FliG_M_Fli_MC_ MFXF hinge C1-6 rotation is not matched in isolated FliG_MC_ complexes, despite high intrinsic flexibility. These results integrated with previous knowledge lead to a model for flagellar switch mechanics.

### A Mechanical Model for the Flagellar Motor Switch

**Figure 10:**
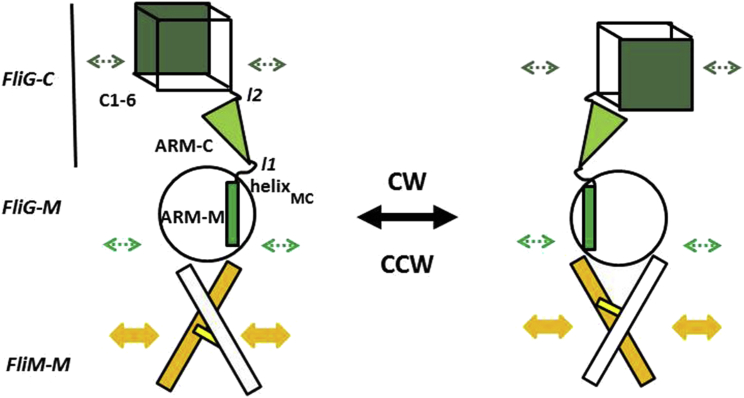
Mechanical Model of the Flagellar Motor Switch Rotary twist of the stiff FliM_M_ domain is transmitted via a localized two-point contact to FliG ARM-M. Two central hinges (l1 = GG motif loop, l2 = MFXF motif loop) bounding ARM-C partition FliG_MC_ into three segments, providing two-stage amplification for FliG_C_ C1-6 reorientation. Long loop l1 flexibility depends on ARM-M/ARM-C stacking interactions. Arrowheads denote location of in situ FliG crosslinks (green) and conformational coupling between adjacent FliM_M_ (yellow).

The model (Figure 10) encapsulates the following mechanical properties.

#### The FliM_M_ Switch Module

FliM_M_ is mechanically stiff, consistent with its role as a dedicated switch module able to propagate conformational transitions distally across FliG to reverse rotor-stator contacts. Complex formation effects a localized change, immobilization of a long loop tethered at both ends to α helices that pivot around the β-sheet center of the αβα sandwich to effect FliG_M_ rotation. The mechanics support the role of FliM_M_ inter-subunit contacts in transverse conformational spread, as localized by in situ crosslinks and indicated by CW mutations (Park et al., 2006), residue coevolution (Pandini et al., 2015a), and electron paramagnetic spectroscopy (Sircar et al., 2015). Atomic force microscopy data have documented the mechanical rigidity of folds with mixed αβ topology (Guzman et al., 2010), whereas unshielded β sheets alone deform readily to accommodate shear compared with more rigid, hydrogen-bonded α-helix backbones (Ackbarow et al., 2007). The FliM_M_ mechanics are in accord with this knowledge.

#### The FliG_M_/ARM-C Mechanical Relay

The FliM_M_FliG_M_ interface couples domain motions via a two-point contact between the FliM_M_ GPGG long loop and FliG_M_ EHPQ and N-terminal helix_MC_ loops. These couplings link the three layers of the FliM_M_ sandwich to ARM-M core helices. The ARM-M fold, composed of rigid α-helical levers linked by short loops, forms an elastic domain resilient to deformation during rotation. Its architecture is consistent with the mechanical properties of ARM proteins (Alfarano et al., 2012). Helix_MC_ leverages ARM-M rotation to ARM-C. Engineered N-terminal PEV and homologous deletions in N-terminal helix_MC_ have predictable effects consistent with a shortened lever arm. The MFVF motif, the second central hinge, amplifies ARM-C rotation to C1-6. Torque from FliM_M_ twist fluctuations is distributed between the hinges, with constrained GG hinge motions compensated for by increased MFVF hinge motions. The flexibility of the composite helix_MC_/GG hinge may be a key source of species variation. Species differences in hinge length and sequence with consequent variations in ARM-C position and domain interactions offer a rationale for the weak ARM-C coevolution signal (Pandini et al., 2015a).

#### The C1-6 Motor Module

In contrast to FliG_M_ the C1-6 module is largely devoid of hinge elements, as contacts with adjacent helices attach the TH onto the C3-6 fold, consistent with coevolution data (Pandini et al., 2015a). Short loops adjacent to the TH fine-tune its orientation relative to C1-6 collective motions. The in situ crosslinks target the loops adjacent to the TH as well as the FliG_M_ interfacial loops (Figure 10). Steric constraints at these end locations would be maximally effective in blocking FliG_MC_ bending motions.

### Implications for Mechanism

Structural models of the flagellar motor switch, reviewed in Stock et al. (2012), seek to explain the large TH reorientation in terms of altered domain contacts. The models agree that FliM_M_ contacts with FliG_MC_ are critical, but differ on the nature of the contacts. One set of models, based on crystallographic data, takes alterations in the FliM_M_-FliG_M_ contact as pivotal and sufficient to explain switching. Other models, based on biochemical evidence and presumed mismatch between FliM and FliG subunit stoichiometry in the C ring, posit the pivotal contact as being between FliM_M_ and FliG ARM-C, although some FliM_M_ units also contact FliG_M_. Our study strengthens the case for a pivotal FliM_M_-FliG_M_ contact.

The PDB: 4FHR complex reveals that complex formation accentuates a large, angular TH reorientation. The reorientation is still 2-fold, or more, lower than is documented in situ. Additional factors will operate in the C ring. First, hinge-bending motions dominant in the isolated complexes are likely to be blocked by adjacent C-ring subunits and might be compensated for by increased rotation. Second, our study does not address whether intra- or inter-molecular FliG_MC_ stacking interactions exist in the C ring. An extended helix_MC_ in the alternative inter-molecular stacking interaction, as recently proposed (Baker et al., 2016, Sircar et al., 2015), would provide greater leverage for rotation of FliG_C_. Intra- and inter-molecular stacking contacts observed in the crystals are similar. A solution study of the salt dependence shows that the conformations are interchangeable (Baker et al., 2016).

The stacked *T. martima* conformation in the PEV deletion structure may represent a CW-locked state (Minamino et al., 2011). However, helix_MC_ is soft due to an unstructured C-terminal segment and N-terminal PEV, and homologous deletions do not switch unstacked to stacked FliG_MC_ configurations. Instead, the stacking interaction is strong enough to deform helix_MC_. The two *H. pylori* FliG_MC_ conformations provide snapshots compatible with the in vivo data (Lam et al., 2012), yet their conformational ensembles overlap with themselves and with other unstacked FliG_MC_ conformers (Figure 8). Therefore, we suggest that the stacking interaction provides a mechanism for conformational selection of an intrinsically flexible protein. Weak stacking interactions summed over the ring will provide the free energy difference to lock in the two rotation states. A functional design for the flagellar motor switch requires flexible downstream elements to rapidly switch conformation with minimal energy dissipation, once switching is initiated. Subunits chemically bonded in distinct conformations would dissipate energy and switch slowly. FliG assembles tightly onto FliF (Levenson et al., 2012) and templates the assembly of FliM(FliN)^3^ distal C-ring complexes (McDowell et al., 2015). Electron microscopy data indicate that the latter may stabilize the FliG ring since it is not clearly visualized in FliFFliG complexes due to presumed disorder (Suzuki et al., 2004), in contrast to the intact C ring (Thomas et al., 2006). Inter-molecular stacking provides a straightforward explanation for how FliG subunits carrying the PEV deletion would favor decreased circumference with a shortened helix_MC_, leading to smaller or more densely packed CW C rings consistent with adaptive remodeling (Lele and Berg, 2015).

### The Broader Context: Relevance and Prospects

This study illustrates the importance of backbone flexibility analysis for interpretation of mutagenesis data. It extends earlier work on the F_0_ ATP synthase (Pandini et al., 2015b) to show that long-range elastic couplings across subunit interfaces contribute to the coevolution signal. Elastic backbone effects have also been noted in coevolution analysis of protein-folding landscapes (Morcos et al., 2014, Sutto et al., 2015). These studies add to the literature, cited in the Introduction, stating that coevolved mutations reflect protein conformational dynamics.

The challenge now is to understand the design principles for evolution of protein-protein interactions. Functional modes should provide a more fine-tuned analysis of the dynamics (Hub and de Groot, 2009). Comparative analysis between natural and designed sequences has shown that optimal backbone flexibility is needed for a strong coevolution signal (Ollikainen and Kortemme, 2013). Optimization constraints may explain why some dynamic couplings have coevolved in the FliM_M_FliG_MC_ signal complex and others have not. X-Ray structure libraries of rotary motor assemblies, with mechanics that can be measured by single-molecule techniques, are an important stimulus for the development of such analytical tools to study the relation between protein evolution and dynamics.

## Experimental Procedures

### Generation of tCONCOORD Conformational Ensembles

The X-ray structure library used in this study was downloaded from the PDB. Secondary structure elements and the contact interface within the FliM_M_FliG_MC_ complex (PDB: 4FHR) are mapped in Figure S1. Component structures (monomers and partial complexes) are described in Figure S2. tCONCOORD produced a conformational ensemble from each X-ray structure. First, atomic pair distances with upper and lower limits were generated from the structure based on tables of bonding interactions (covalent bonds, hydrogen bonds, salt bridges, etc.) constructed from statistical analysis of the PDB database (de Groot et al., 1997). Second, a new structure was built starting from atoms positioned randomly within a bounding volume around their X-ray coordinates. Successive iterations were performed until convergence was achieved upon satisfaction of the distance constraints or an iteration limit (500) was reached. The structure was rebuilt many times to generate (256)^2^ = 65,536 equilibrium conformations with full atom detail. tCONCOORD samples large conformational protein transitions by breakage of labile hydrogen bonds solvated by surrounding residues, as validated by test cases and MD simulations (Seeliger et al., 2007).

### Principal Component Analysis

PCA, specifically of MD trajectories, was introduced when Amadei et al. (1993) showed that the configurational space can be partitioned into an “essential” subspace with few degrees of freedom describing large-scale slow anharmonic motions, with the remaining space describing local fluctuations. Functional motions of biologically relevant conformational transitions belong to the essential subspace defined by the first few PCs. These physically represent the largest-amplitude collective motions in the macromolecular assembly (de Groot et al., 1996). The variance (σ^2^) was taken as a measure of “motion” (Pandini et al., 2015b). The combined variance of the statistically independent, first three PCs (or subset) from the average structure was obtained by summation. Geometric angular distributions between selected vector pairs were used to compute the torsional stiffness and bending moments.

### Network Analysis

The conformational dynamics of four-residue fragments were encoded with the SA (Figure S2) for elucidation of the mechanical relays underlying collective PC motions and comparison with the coevolution network. Frequently occurring conformations from 798 high-resolution X-ray structures were extracted as representative fragment states (letters) (Pandini et al., 2010). The SA provides an enriched string set of local conformational states for accurate reconstruction of protein fold. Statistically significant correlations were determined and analyzed with GSATools (Pandini et al., 2013).

### Coevolution Analysis

Pfam protein sequence families FliM_M_ (PF02154), FliG_M_ (PF14821), and FliG_C_ (PF01706) (Finn et al., 2010) were filtered at 80% redundancy level. PSICOV-based analysis of residue coevolution between FliM and FliG (Pandini et al., 2015a) was supplemented with direct coupling analysis (Morcos et al., 2011) to increase contact prediction accuracy (Jones et al., 2014). Sequences were matched based on organism membership and genomic locus proximity (<100 genes, *fliG*-*fliM* distance = 18 ± 24). The final dataset contained more than 1,400 non-redundant, concatenated sequences. The coevolution network was constructed from the top 1.5% correlations, a cut-off intermediate between 2σ (2.2%) and 3σ (0.3%). Randomized libraries generated by shuffling within MSA residue positions assessed significance (Pandini et al., 2015a).

See Supplemental Experimental Procedures for operational details and formalism.

## Author Contributions

Conceptualization: A.P., F.M., and S.K.; Methodology: A.P., F.M., and S.K.; Software: A.P. and F.M.; Validation: S.K.; Writing – First Draft: A.P. and S.K.; Writing – Review & Editing: A.P., F.M., and S.K.; Visualization: F.M. and S.K.; Supervision/Project Administration: S.K.; Funding Acquisition: F.M. and S.K.

## Supplemental Information

Document S1. Supplemental Experimental Procedures and Figures S1–S7

Movie S1. PDB: 4FHR PC1 Bending Motion: Side View, Related to Figure 3C

Movie S2. PDB: 4FHR PC3 Rotary Motion: En-Face View, Related to Figure 3C

Movie S3. PDB: 4FHR PEV Deletion PC3 Rotary Motion: En-Face View, Related to Figure 6A

Movie S4. PDB: 2HP7 Rotary Motion, En-Face View: First Half, PC1 and Second Half, PC Average, Related to Figure 7D

Movie S5. PDB: 3SOH Rotary Motion, En-Face View: First Half, PC1 and Second Half, PC Average, Related to Figure 7D

Movie S6. PDB: 4FQ0 Rotary Motion, En-Face View: First Half, PC1 and Second Half, PC Average, Related to Figure 7D

Movie S7. PDB: 3AJC Rotary Motion, En-Face View: First Half, PC1 and Second Half, PC Average, Related to Figure 8F

Movie S8. PDB: 3USW Rotary Motion, En-Face View: First Half, PC1 and Second Half, PC Average, Related to Figure 8F

Model S1. PDB: 4FHR FliMM Dynamic Correlation Network, Related to Figure 6

Model S2. PDB: 4FHR FliMM-FliGM Inter-subunit Dynamic Correlation Network, Related to Figure 6

Model S3. PDB: 4FHR FliGM Dynamic Correlation Network, Related to Figure 6

Model S4. PDB: 4FHR FliMM-FliGM Inter-subunit Coevolution Network, Related to Figure 6

Document S2. Article plus Supplemental Information

## References

[bib1] Ackbarow T., Chen X., Keten S., Buehler M.J. (2007). Hierarchies, multiple energy barriers, and robustness govern the fracture mechanics of alpha-helical and beta-sheet protein domains. Proc. Natl. Acad. Sci. USA.

[bib2] Alfarano P., Varadamsetty G., Ewald C., Parmeggiani F., Pellarin R., Zerbe O., Pluckthun A., Caflisch A. (2012). Optimization of designed armadillo repeat proteins by molecular dynamics simulations and NMR spectroscopy. Protein Sci..

[bib3] Amadei A., Linssen A.B., Berendsen H.J. (1993). Essential dynamics of proteins. Proteins.

[bib4] Bai F., Che Y.S., Kami-ike N., Ma Q., Minamino T., Sowa Y., Namba K. (2013). Populational heterogeneity vs. temporal fluctuation in Escherichia coli flagellar motor switching. Biophys. J..

[bib5] Baker M.A., Hynson R.M., Ganuelas L.A., Mohammadi N.S., Liew C.W., Rey A.A., Duff A.P., Whitten A.E., Jeffries C.M., Delalez N.J. (2016). Domain-swap polymerization drives the self-assembly of the bacterial flagellar motor. Nat. Struct. Mol. Biol..

[bib6] Bray D., Duke T. (2004). Conformational spread: the propagation of allosteric states in large multiprotein complexes. Annu. Rev. Biophys. Biomol. Struct..

[bib7] Brown P.N., Hill C.P., Blair D.F. (2002). Crystal structure of the middle and C-terminal domains of the flagellar rotor protein FliG. EMBO J..

[bib8] Czub J., Grubmuller H. (2014). Rotation triggers nucleotide-independent conformational transition of the empty beta subunit of F(1)-ATPase. J. Am. Chem. Soc..

[bib9] de Groot B.L., van Aalten D.M., Amadei A., Berendsen H.J. (1996). The consistency of large concerted motions in proteins in molecular dynamics simulations. Biophys. J..

[bib10] de Groot B.L., van Aalten D.M., Scheek R.M., Amadei A., Vriend G., Berendsen H.J. (1997). Prediction of protein conformational freedom from distance constraints. Proteins.

[bib11] Dyer C.M., Vartanian A.S., Zhou H., Dahlquist F.W. (2009). A molecular mechanism of bacterial flagellar motor switching. J. Mol. Biol..

[bib12] Fernandez A., Scheraga H.A. (2003). Insufficiently dehydrated hydrogen bonds as determinants of protein interactions. Proc. Natl. Acad. Sci. USA.

[bib13] Finn R.D., Mistry J., Tate J., Coggill P., Heger A., Pollington J.E., Gavin O.L., Gunasekaran P., Ceric G., Forslund K. (2010). The Pfam protein families database. Nucleic Acids Res..

[bib14] Guzman D.L., Randall A., Baldi P., Guan Z. (2010). Computational and single-molecule force studies of a macro domain protein reveal a key molecular determinant for mechanical stability. Proc. Natl. Acad. Sci. USA.

[bib15] Highsmith S., Wang C.C., Zero K., Pecora R., Jardetzky O. (1982). Bending motions and internal motions in myosin rod. Biochemistry.

[bib16] Hub J.S., de Groot B.L. (2009). Detection of functional modes in protein dynamics. PLoS Comput. Biol..

[bib17] Jones D.T., Singh T., Kosciolek T., Tetchner S. (2014). MetaPSICOV: combining coevolution methods for accurate prediction of contacts and long range hydrogen bonding in proteins. Bioinformatics.

[bib18] Lam K.H., Ip W.S., Lam Y.W., Chan S.O., Ling T.K., Au S.W. (2012). Multiple conformations of the FliG C-terminal domain provide insight into flagellar motor switching. Structure.

[bib19] Lee L.K., Ginsburg M.A., Crovace C., Donohoe M., Stock D. (2010). Structure of the torque ring of the flagellar motor and the molecular basis for rotational switching. Nature.

[bib20] Lele P.P., Berg H.C. (2015). Switching of bacterial flagellar motors is triggered by mutant FliG. Biophys. J..

[bib21] Levenson R., Zhou H., Dahlquist F.W. (2012). Structural insights into the interaction between the bacterial flagellar motor proteins FliF and FliG. Biochemistry.

[bib22] Lloyd S.A., Blair D.F. (1997). Charged residues of the rotor protein FliG essential for torque generation in the flagellar motor of *Escherichia coli*. J. Mol. Biol..

[bib23] Lux R., Kar N., Khan S. (2000). Overproduced *Salmonella typhimurium* flagellar motor switch complexes. J. Mol. Biol..

[bib24] Ma Q., Nicolau D.V., Maini P.K., Berry R.M., Bai F. (2012). Conformational spread in the flagellar motor switch: a model study. PLoS Comput. Biol..

[bib25] Magariyama Y., Yamaguchi S., Aizawa S. (1990). Genetic and behavioral analysis of flagellar switch mutants of *Salmonella typhimurium*. J. Bacteriol..

[bib26] McDowell M.A., Marcoux J., McVicker G., Johnson S., Fong Y.H., Stevens R., Bowman L.A., Degiacomi M.T., Yan J., Wise A. (2015). Characterisation of Shigella Spa33 and Thermotoga FliM/N reveals a new model for C-ring assembly in T3SS. Mol. Microbiol..

[bib27] Minamino T., Imada K., Kinoshita M., Nakamura S., Morimoto Y.V., Namba K. (2011). Structural insight into the rotational switching mechanism of the bacterial flagellar motor. PLoS Biol..

[bib28] Morcos F., Jana B., Hwa T., Onuchic J.N. (2013). Coevolutionary signals across protein lineages help capture multiple protein conformations. Proc. Natl. Acad. Sci. USA.

[bib29] Morcos F., Pagnani A., Lunt B., Bertolino A., Marks D.S., Sander C., Zecchina R., Onuchic J.N., Hwa T., Weigt M. (2011). Direct-coupling analysis of residue coevolution captures native contacts across many protein families. Proc. Natl. Acad. Sci. USA.

[bib30] Morcos F., Schafer N.P., Cheng R.R., Onuchic J.N., Wolynes P.G. (2014). Coevolutionary information, protein folding landscapes, and the thermodynamics of natural selection. Proc. Natl. Acad. Sci. USA.

[bib31] Ollikainen N., Kortemme T. (2013). Computational protein design quantifies structural constraints on amino acid covariation. PLoS Comput. Biol..

[bib32] Pandini A., Fornili A., Kleinjung J. (2010). Structural alphabets derived from attractors in conformational space. BMC Bioinformatics.

[bib33] Pandini A., Fornili A., Fraternali F., Kleinjung J. (2013). GSATools: analysis of allosteric communication and functional local motions using a structural alphabet. Bioinformatics.

[bib34] Pandini A., Kleinjung J., Rasool S., Khan S. (2015). Coevolved mutations reveal distinct architectures for two core proteins in the bacterial flagellar motor. PLoS One.

[bib35] Pandini A., Kleinjung J., Taylor W.R., Junge W., Khan S. (2015). The phylogenetic signature underlying ATP synthase c-ring compliance. Biophys. J..

[bib36] Park S.Y., Lowder B., Bilwes A.M., Blair D.F., Crane B.R. (2006). Structure of FliM provides insight into assembly of the switch complex in the bacterial flagella motor. Proc. Natl. Acad. Sci. USA.

[bib37] Paul K., Brunstetter D., Titen S., Blair D.F. (2011). A molecular mechanism of direction switching in the flagellar motor of *Escherichia coli*. Proc. Natl. Acad. Sci. USA.

[bib38] Sagi Y., Khan S., Eisenbach M. (2003). Binding of the chemotaxis response regulator CheY to the isolated, intact switch complex of the bacterial flagellar motor: lack of cooperativity. J. Biol. Chem..

[bib39] Sarkar M.K., Paul K., Blair D. (2010). Chemotaxis signaling protein CheY binds to the rotor protein FliN to control the direction of flagellar rotation in *Escherichia coli*. Proc. Natl. Acad. Sci. USA.

[bib40] Seeliger D., Haas J., de Groot B.L. (2007). Geometry-based sampling of conformational transitions in proteins. Structure.

[bib41] Sfriso P., Duran-Frigola M., Mosca R., Emperador A., Aloy P., Orozco M. (2016). Residues coevolution guides the systematic identification of alternative functional conformations in proteins. Structure.

[bib42] Sircar R., Borbat P.P., Lynch M.J., Bhatnagar J., Beyersdorf M.S., Halkides C.J., Freed J.H., Crane B.R. (2015). Assembly states of FliM and FliG within the flagellar switch complex. J. Mol. Biol..

[bib43] Sourjik V., Berg H.C. (2002). Binding of the *Escherichia coli* response regulator CheY to its target measured in vivo by fluorescence resonance energy transfer. Proc. Natl. Acad. Sci. USA.

[bib44] Stock D., Namba K., Lee L.K. (2012). Nanorotors and self-assembling macromolecular machines: the torque ring of the bacterial flagellar motor. Curr. Opin. Biotechnol..

[bib45] Sutto L., Marsili S., Valencia A., Gervasio F.L. (2015). From residue coevolution to protein conformational ensembles and functional dynamics. Proc. Natl. Acad. Sci. USA.

[bib46] Suzuki H., Yonekura K., Namba K. (2004). Structure of the rotor of the bacterial flagellar motor revealed by electron cryomicroscopy and single-particle image analysis. J. Mol. Biol..

[bib47] Thomas D.R., Francis N.R., Xu C., DeRosier D.J. (2006). The three-dimensional structure of the flagellar rotor from a clockwise-locked mutant of *Salmonella enterica* serovar *Typhimurium*. J. Bacteriol..

[bib48] Vartanian A.S., Paz A., Fortgang E.A., Abramson J., Dahlquist F.W. (2012). Structure of flagellar motor proteins in complex allows for insights into motor structure and switching. J. Biol. Chem..

[bib49] Yuan J., Berg H.C. (2013). Ultrasensitivity of an adaptive bacterial motor. J. Mol. Biol..

[bib50] Zhou J., Lloyd S.A., Blair D.F. (1998). Electrostatic interactions between rotor and stator in the bacterial flagellar motor. Proc. Natl. Acad. Sci. USA.

