# Supplementary material for: The Gearbox of the Bacterial Flagellar Motor Switch
Source: Structure. 2016 Jul 6;24(7):1209–20. doi: 10.1016/j.str.2016.05.012 (PMC4938800; doi:10.1016/j.str.2016.05.012)
Supplement: Document S1. Supplemental Experimental Procedures and Figures S1–S7 [file mmc1.pdf]

Structure, Volume *24*

Supplemental Information

The Gearbox of the Bacterial Flagellar Motor Switch

Alessandro Pandini, Faruck Morcos, and Shahid Khan

SUPPLEMENTAL INFORMATION

Supplemental Data

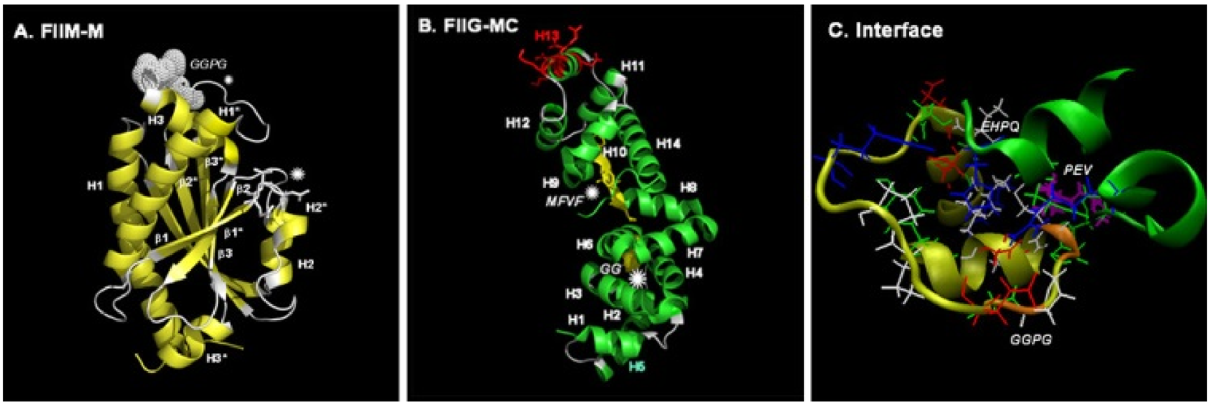

Figure S1, Related to Figure 1: 4FHR Structure Index. *4FHR domain structures and inter-helix loops (white backbone). Asterisks mark segments with high B-factors computed from the conformational ensemble; asterisk size is proportional to B-factor value.* ***A****. FliM*$_M$ *domain. GGPG motif = white spheres.* ***B****. FliG*$_{MC}$ *domains. GG / MFVF motifs (yellow segments), TH (H13, red letters / charged residue sidechains) and helix*$_{MC}$ *(H5, pale green letters) are marked. Helices H1-H5 form the FliG*$_M$ *ARM domain (ARM-M). FliG*$_C$ *has two sub-domains either side of the MFXF motif, N-terminal ARM-C (H6-H8), and C-terminal six helix bundle C1-6 (H9-H14).* ***C.*** *Contact residue side-chains at the FliM*$_M$ *(yellow backbone) and FliG*$_M$ *(green backbone) interface coloured according to residue type (non-polar (white), acidic (red), basic (blue)). GGPG (orange backbone) and PEV (magenta sidechains) residues are marked.*

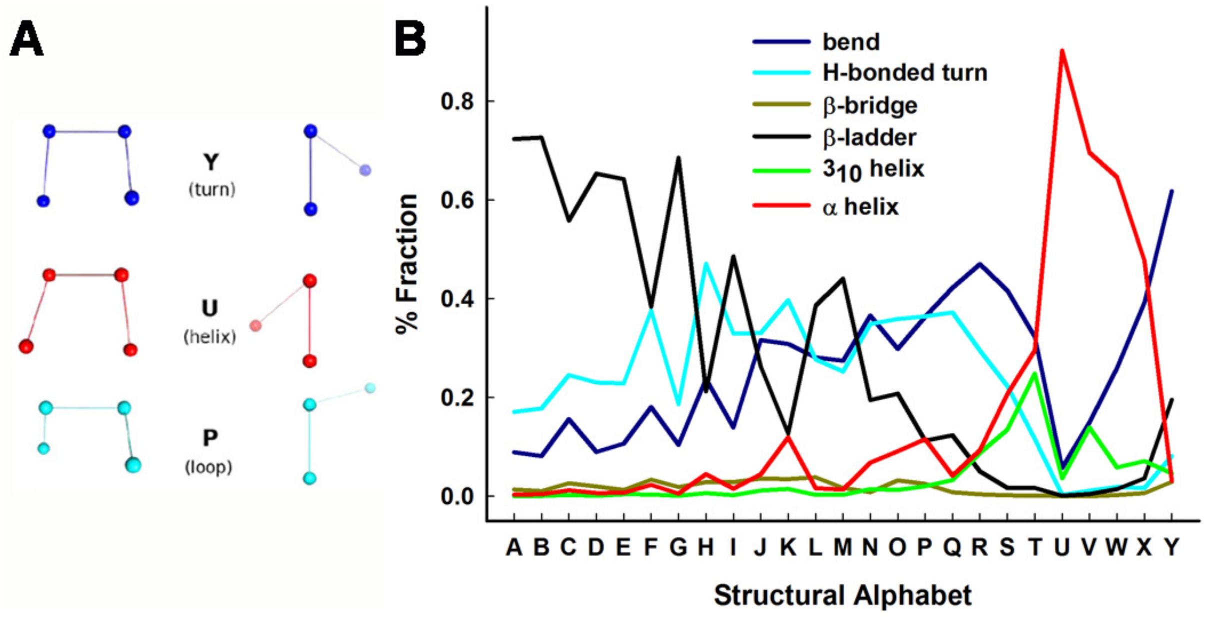

Figure S2, Related to Figure 5A: A. Examples of SA letters. *The SA has 25 letters representing fragments of 4 consecutive C*$^{\alpha}$ *atoms. Each letter represents a prototypical conformational state. Loop specific conformations are as listed in STRIDE. "The relation between the two views is a 90° rotation around a vertical axis in the paper plane and an adjustment to align the two central atoms to a Newman projection" (from (Pandini et al., 2010) with permission).* ***B. SA secondary structure assignment.*** *The letters were assigned based on screen of 798 high-resolution X-ray structures in PDB. The figure presents a more detailed correspondence of SA with secondary structure elements than initially published (Pandini et al., 2010). Secondary structure nomenclature is in accordance with the DSSP database (Touw et al., 2015).*

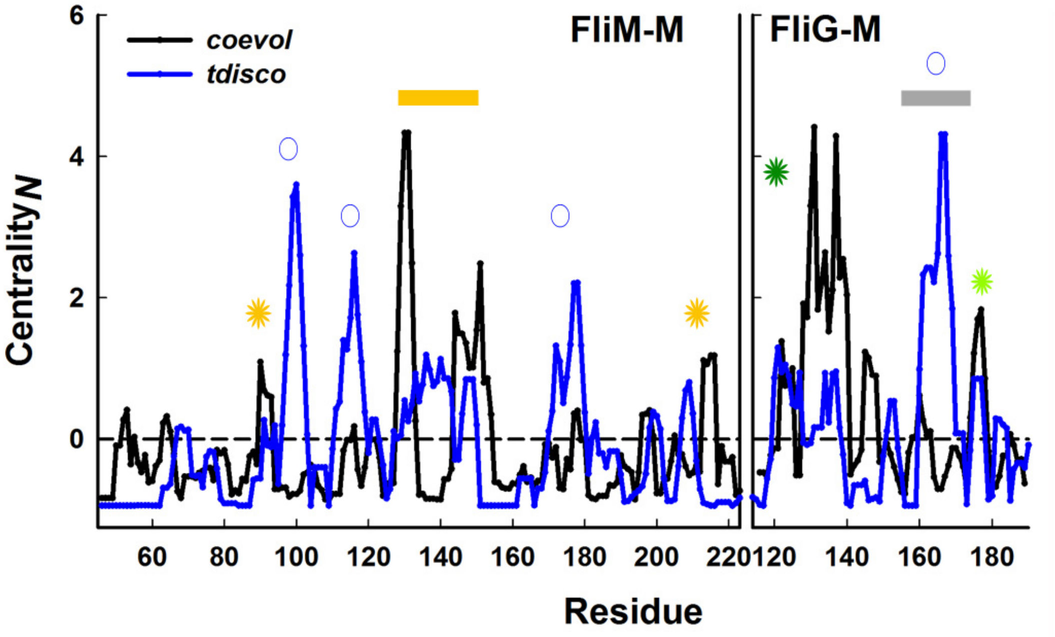

Figure S3, Related to Figure 6C: Coevolution and dynamic network centrality. *Centrality$_N$ = ((Cent$_{residue}$ – Cent$_{mean}$)/σ_{cent}), where Cent$_{mean}$ and σ_{cent} are mean and standard deviation respectively of the residue centrality (Cent$_{residue}$). The GPGG motif loop (yellow bar) and EHPQ motif (green asterisk) are part of the FliMFliG interface. Coevolution signal from these interfacial contacts was reported (Pandini et al., 2015a). FliM$_M$ sheet β1 and β3* (yellow asterisks) are dynamically coupled to FliG$_M$ core helix H2 adjacent to the EHPQ motif C-terminus. These elements are not in contact as seen from the structural map (Figure 6C). The helix$_{MC}$ (grey bar) C-terminal GG loop (light green asterisk) is also important for interface coevolution and dynamics. Other dynamic couplings (open circles), notably helix$_{MC}$ itself, and other strands in the FliM$_M$ β core lack a coevolution signal. P$_{corr}$ = 0.03.*

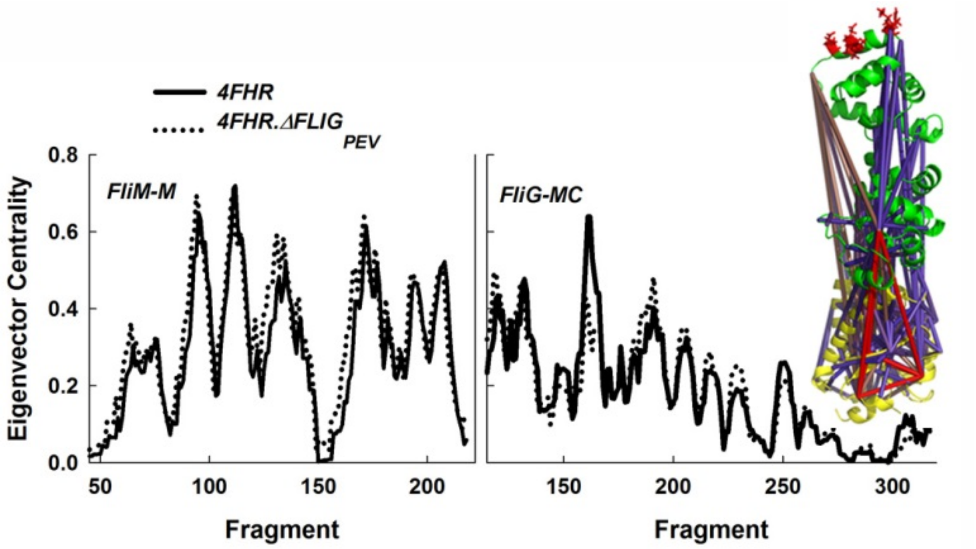

Figure S4, Related to Figure 6A: Perturbation of FliG$_{MC}$ dynamics by ΔPEV. *The energy minimized T. maritima 4FHR PEV deletion protein complex had 798 fewer atom-to-atom contacts in FliG$_{M}$, of which 431 contacts were formed by the deleted residues. The interfacial contacts between FliM$_{M}$ and FliG$_{M}$ were reduced from 530 to 468. T. maritima PEV = PQV (H. pylori), VQY (A. aeolicus). The 4FHR network centrality (± FliG$_{MC}$ PEV) P$_{corr}$ = 0.91.* ***Image:*** *Top FliG$_{M}$-FliG$_{MC}$ interfacial correlations mapped onto the 4FHR deletion mutant structure.*

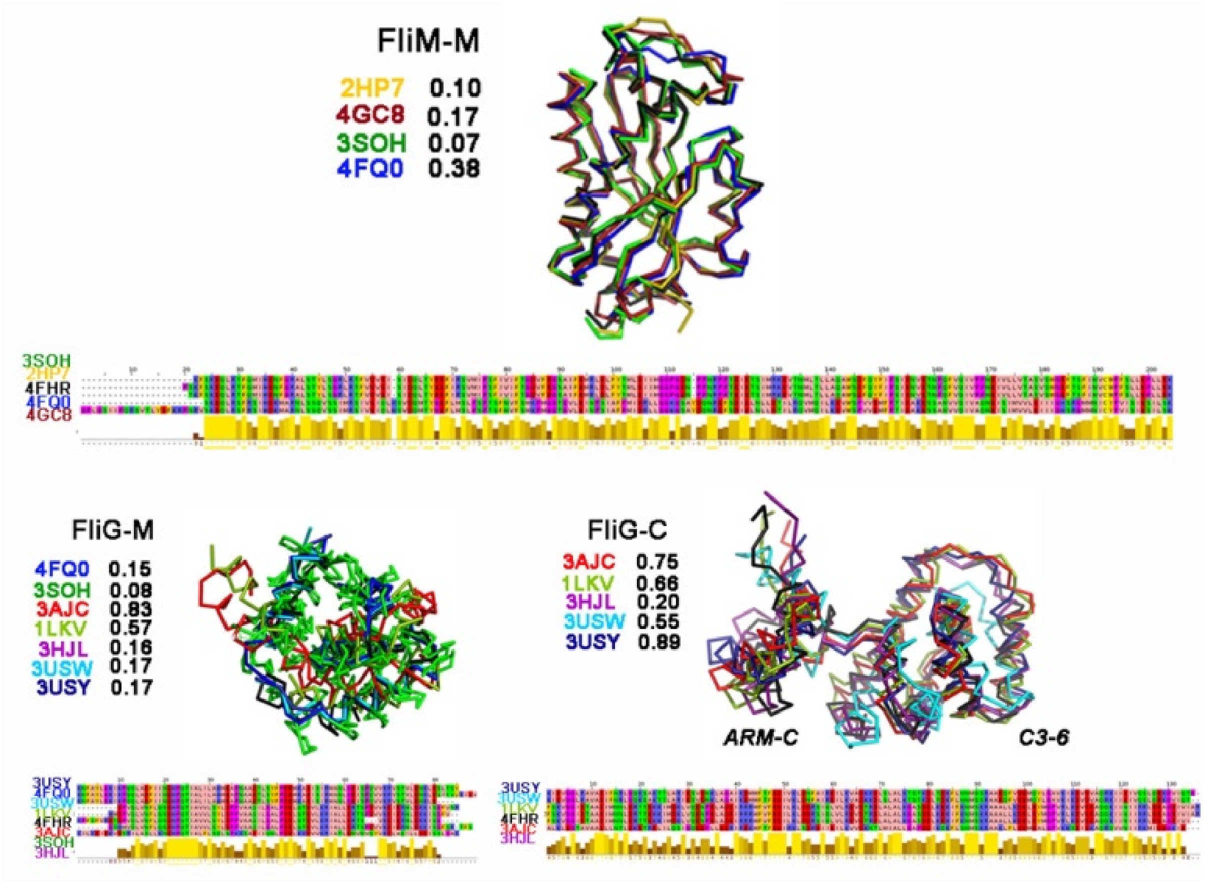

Figure S5, Related to Figures 7 & 8: Conformational variability between flagellar motor protein structures. *FliM$_M$ (T. maritima 2HP7(Park et al.,2006), H. pylori GC8 (Lam et al., 2013)), FliG$_{MC}$,(T. maritima 1LKV (Brown et al.,2002), 3AJC (Minamino et al.,2011), 3USY, 3USW(Lam et al.,2012); FliG$_{NMC}$ (A. aeolicus 3HJL (Lee et al.,2010); FliM$_M$FliG$_M$ (T. maritima 3SOH (Paul et al., 2011), H. pylori 4FQ0 (Lam et al., 2013)) and FliM$_M$FliG$_{MC}$ (T. maritima 4FHR). Superimpositions of the X-ray crystal structures for each domain are shown above the MSA of their sequences. The residues in the MSA are coloured according to type (Zappo colouring). T. maritima motility is powered by a single, monopolar flagellum with dominantly CW rotation, interspersed with brief CCW episodes that reorient the bacteria (Gluch et al., 1995). The epsilon proteobacterium, H. pylori, has a monopolar bundle with sheathed flagella to withstand acid pH in the human stomach (Lertsethtakarn et al., 2011). A. aeolicus is an ancient, thermophilic species like T. maritima, with monopolar polytrichous flagella like H. pylori (Takekawa et al., 2015). The RMSDs (angstrom^2) of the structures from the reference 4FHR structure are listed. The FliM$_M$ domain structures (T.maritima RMSD = 0.08 ± 0.02 A^2; H. pylori RMSD = 0.27 ± 0.15 A^2) were arguably different between species; but there was no meaningful difference between the FliG$_M$ domain structures (A. aeolicus RMSD = 0.16 A^2; T. maritima = 0.49 ± 0.38 A^2; H. pylori = 0.16 ± 0.01 A^2). T. maritima FliG$_{MC}$ structures 1LKV and 3AJC had greater RMSD from 4FHR FliG$_M$ compared to the A. aeolicus or H. pylori structures. The FliG$_c$ domain structures were the most variable. The FliG$_c$ RMSDs of other T. maritima structures from 4FHR were comparable to structures from other species (A. aeolicus RMSD = 0.2 A^2; T. maritima = 0.81 ± 0.08 A^2; H. pylori = 0.72 ± 0.24 A^2), as for FliG$_M$,. The 3AJC structure has the PEV deletion, homologous to the PAA deletion in the CW-locked Salmonella mutant.*

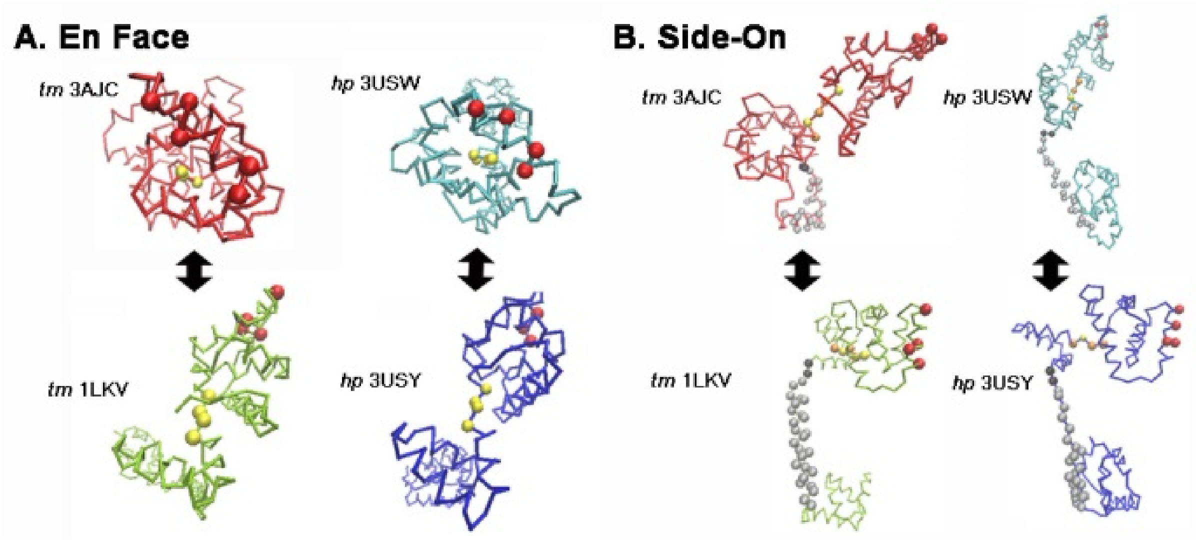

Figure S6, Related to Figure 9A: Comparison of the most divergent T. maritima (tm) and H. pylori (hp) structures. A. *En-face views show co-axial orientation of C1-6 in (3AJC, 3USW), and off-axis orientation (~ 45° tilt angle) of C1-6 relative to FliG$_M$ in (1LKV, 3USY).* ***B.*** *Side-on views show helix$_{MC}$ (grey spheres) split into two segments in 3AJC, but linearly extended in the other structures. The N-terminal half of helix$_{MC}$ contacts ARM-M in the hp structures. GG pair (black spheres); MFXF motif (yellow spheres); TH charged residues (red spheres).*

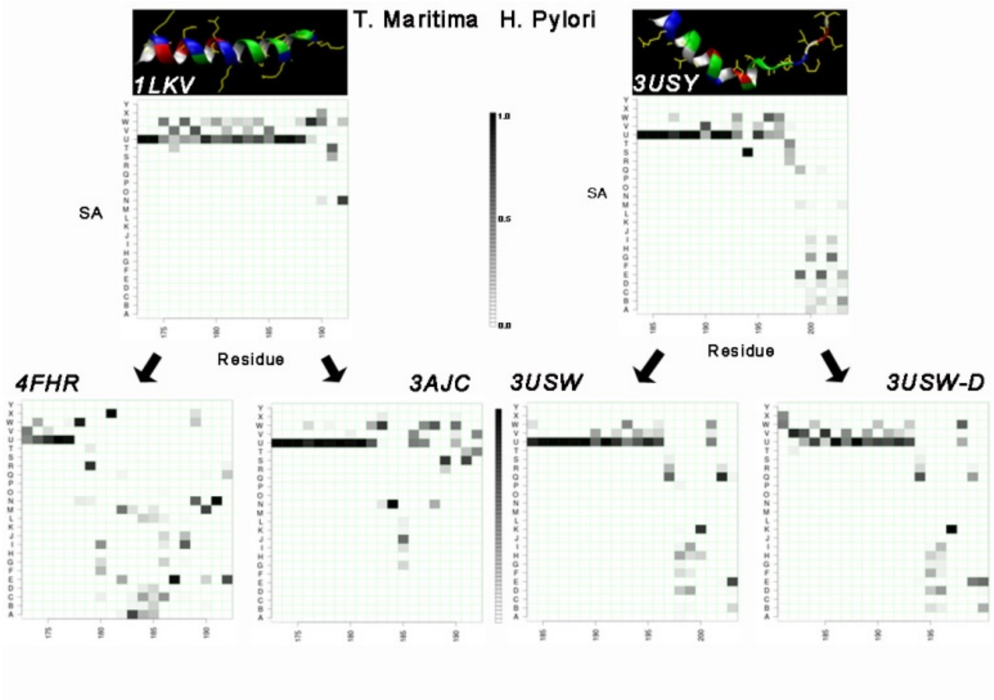

Figure S7, Related to Figure 9A: Helix$_{MC}$ dynamics. *Helix$_{MC}$ - GG loop (25 residue segment) conformational spectra. The segment includes 7 residues grafted from 1LKV / 3USW conformations to others where these were not resolved. Greyscale bars show frequency of the SA-encoded conformations as in Figure 5. Arrows link graft donor–acceptor structures. In isolation, the extended helix$_{MC}$ remains α-helical in T. maritima 1LKV. In contrast, the C-terminal third of H. pylori 3USY adopts β-sheet conformations. In the 3AJC / 4FHR structures with the intramolecular ARM-M H11 / ARM-C H13-H14 stacking contact (Vartanian et al.,2012) the helix, including the grafted residues, is disrupted. In 3AJC, the central residues adopt loop conformations. In 4FHR, most (C-terminal two-third) of helix$_{MC}$ is non-helical; possibly due to torsion created by FliM$_{M.}$ In the H. pylori 3USW or its deletion (3USW-D), helix$_{MC}$ dynamics are similar to 3USY.* ***Images*** *show the segment in the donor structures. Backbone coloured according to residue type (acid (red); basic (blue); polar (green); hydrophobic (white)) indicates propensity for internal solvation. Crystal contacts (yellow sidechains).*

Supplemental Experimental Procedures

Structure preparation and tCONCOORD simulation

The PDB structure files were prepared for tCONCOORD simulations at neutral pH and 300°K in Molecular Operating Environment 2013.08 (Chemical Computing Group Inc., Montreal, QC H3A 2R7, Canada). Missing residues were grafted in from alternate conformations. The system was energy-minimized with the OPLS-AA force field before simulation within the GROMACS 4.5.5 environment (Pronk et al., 2013), as in the recent extensive comparison of tCONCOORD and MD (Fornili et al., 2013) . Default solvation score was 2.2. Comparison with experimental B-factors and geometrical analyses of the conformer ensembles were performed with the GROMACS *g-rmsd* and *g-sgangle* functions respectively. Crystal contacts were extracted from the PDB files with the CCP4 suite *ncont* utility (Winn et al., 2011).

PCA and network analysis

The PCs were generated by diagonalization of the covariance matrix of C$^{\alpha}$ positions after removal of the overall rotational and translational motions. The combined variance of the PCs was obtained by summation. For example,

$$(\sigma_{PC1\text{-}3})^2 = (\sigma_{PC1})^2 + (\sigma_{PC2})^2 + (\sigma_{PC3})^2 \quad (1)$$

The SA-encoded tCONCOORD ensembles formed string sets. The variance at fragment positions was given by the Shannon entropy, S_f.

$$S_f = -\sum_{j=1}^{k} p_{ij}.log_2\, p_{ij}; \quad (2)$$

where p_{ij} is the fraction of the ensemble with fragment position *i* occupied by SA letter *j*.

The SA-encoded covariance matrix was used to generate a network model; with the residues as nodes and the correlations as edges. The contribution of a node to the network scaled with its connectivity, estimated by the eigenvector centrality, *E*, calculated directly from the correlation matrix:

$$E.(M)_{corr} = E.\lambda; \quad (3)$$

where $(M)_{corr}$ is the correlation matrix and λ the corresponding eigenvalue.

The correlation of conformational changes in a pair of protein segments *(i,j)* was calculated as normalized mutual information (nMI_{local}) between the associated columns in the structural string alignment.

$$nMI_{local}(Ci; Cj) = (I(Ci; Cj) - \varepsilon(Ci; Cj))/H(Ci, Cj) \quad (4)$$

where *Ci* and *Cj* are the relevant columns in the structural string, *I(Ci;Cj)* is the mutual information between them, *H(Ci,Cj)* is the joint entropy, and *ε(Ci;Cj)* is the expected finite size error. Significance (nMI > 0) was determined with the false discovery rate test by comparison against a randomized background distribution obtained by shuffling. The top correlations (nMI > 0.15, <10% of the total) were mapped onto the PDB structures.

The correlation between local and global motions was calculated as the nMI (nMI_{PC}) between the array of fragment states and the array of PC states obtained from the C$^{\alpha}$ covariance matrix.

$$nMI_{PC} = I(Ci; sPCj) / H(Ci, sPCj) \quad (5)$$

where *Ci* is the vector of states sampled by fragment *i*, *sPCj* is the vector of global states associated with the *j*th PC , *I*(*Ci*;*sPCj*) is their MI, and *H*(*Ci*,*sPCj*) is their joint entropy. Further details are given in (Pandini et al.,2012. Pandini et al., 2015b).

Mechanics

The torsional stiffness, K, is given by the equation $\sigma^2 = k_B T K^{-1}$; where k_B = Boltzmann constant and T = temperature. The bending moment, M is computed from the Euler-Bernoulli equation $(\frac{d^2w}{dx^2}) = -M/EI$; where E = Young's modulus, I = area moment of inertia, w = deflection, x = length. Force and M balance, applicable to each segment, apply over the beam.

Supplemental References

Fornili, A., Pandini, A., Lu, H.C., and Fraternali, F. (2013). Specialized Dynamical Properties of Promiscuous Residues Revealed by Simulated Conformational Ensembles. Journal of chemical theory and computation *9*, 5127-5147.

Gluch, M.F., Typke, D., and Baumeister, W. (1995). Motility and thermotactic responses of Thermotoga maritima. Journal of bacteriology *177*, 5473-5479.

Lam, K.H., Lam, W.W., Wong, J.Y., Chan, L.C., Kotaka, M., Ling, T.K., Jin, D.Y., Ottemann, K.M., and Au, S.W. (2013). Structural basis of FliG-FliM interaction in Helicobacter pylori. Molecular microbiology *88*, 798-812.

Lertsethtakarn, P., Ottemann, K.M., and Hendrixson, D.R. (2011). Motility and chemotaxis in Campylobacter and Helicobacter. Annual review of microbiology *65*, 389-410.

Paul, K., Gonzalez-Bonet, G., Bilwes, A.M., Crane, B.R., and Blair, D. (2011). Architecture of the flagellar rotor. The EMBO journal *30*, 2962-2971.

Pronk, S., Pall, S., Schulz, R., Larsson, P., Bjelkmar, P., Apostolov, R., Shirts, M.R., Smith, J.C., Kasson, P.M., van der Spoel, D., *et al.* (2013). GROMACS 4.5: a high-throughput and highly parallel open source molecular simulation toolkit. Bioinformatics (Oxford, England) *29*, 845-854.

Takekawa, N., Nishiyama, M., Kaneseki, T., Kanai, T., Atomi, H., Kojima, S., and Homma, M. (2015). Sodium-driven energy conversion for flagellar rotation of the earliest divergent hyperthermophilic bacterium. Scientific reports *5*, 12711.

Touw, W.G., Baakman, C., Black, J., te Beek, T.A., Krieger, E., Joosten, R.P., and Vriend, G. (2015). A series of PDB-related databanks for everyday needs. Nucleic acids research *43*, D364-368.

Winn, M.D., Ballard, C.C., Cowtan, K.D., Dodson, E.J., Emsley, P., Evans, P.R., Keegan, R.M., Krissinel, E.B., Leslie, A.G., McCoy, A., *et al.* (2011). Overview of the CCP4 suite and current developments. Acta crystallographica *67*, 235-242.

Molecular Models

MM S1, Related to Figure 6: *4FHR FliM$_M$ dynamic correlation network*
MM S2, Related to Figure 6: *4FHR FliM$_M$ – FliG$_M$ inter-subunit dynamic correlation network*
MM S3, Related to Figure 6: *4FHR FliG$_M$ dynamic correlation network*
MM S4, Related to Figure 6: *4FHR FliM$_M$ – FliG$_M$ inter-subunit coevolution network*

Movies

Movie S1, Related to Figure 3C: *4FHR PC1 bending motion (side view)*
Movie S2, Related to Figure 3C: *4FHR PC3 rotary motion (en-face view)*
Movie S3, Related to Figure 6A: *4FHR PEV deletion PC3 rotary motion (en-face view)*
Movie S4, Related to Figure 7D: *2HP7 rotary motion (en-face view). PC1 (first half) and PC average (second half)*
Movie S5, Related to Figure 7D: *3SOH rotary motion (en-face view). PC1 (first half) and PC average (second half)*
Movie S6, Related to Figure 7D: *4FQ0 rotary motion (en-face view). PC1 (first half) and PC average (second half)*
Movie S7, Related to Figure 8F: *3AJC rotary motion (en-face view). PC1 (first half) and PC average (second half)*
Movie S8, Related to Figure 8F: *3USW rotary motion (en-face view). PC1 (first half) and PC average (second half)*
