# Supplementary figures and images for: The Gearbox of the Bacterial Flagellar Motor Switch
Source: Structure. 2016 Jul 6;24(7):1209–20. doi: 10.1016/j.str.2016.05.012 (PMC4938800; doi:10.1016/j.str.2016.05.012)

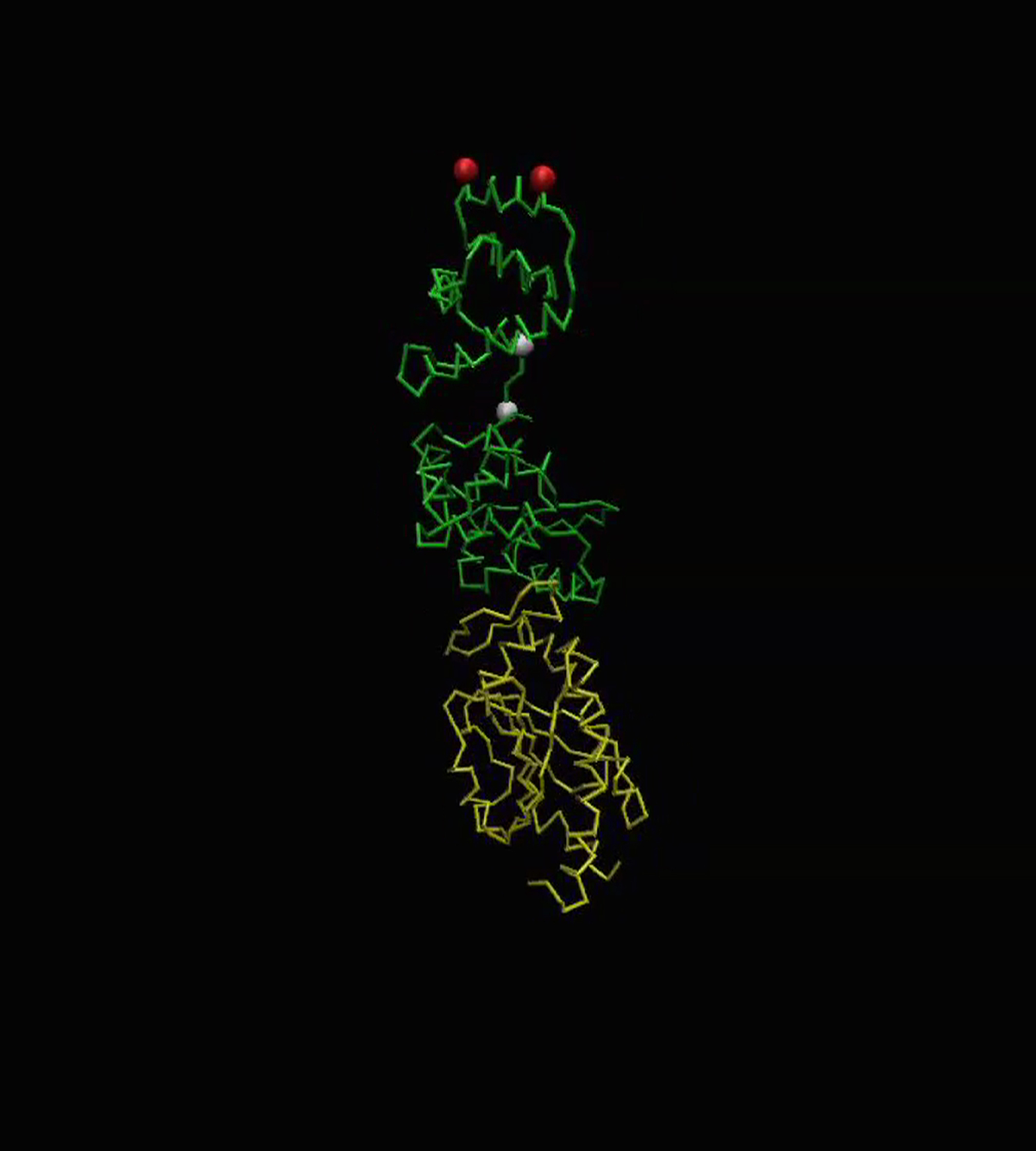

Supplement: Movie S1. PDB: 4FHR PC1 Bending Motion: Side View, Related to Figure 3C [file mmc2.jpg]

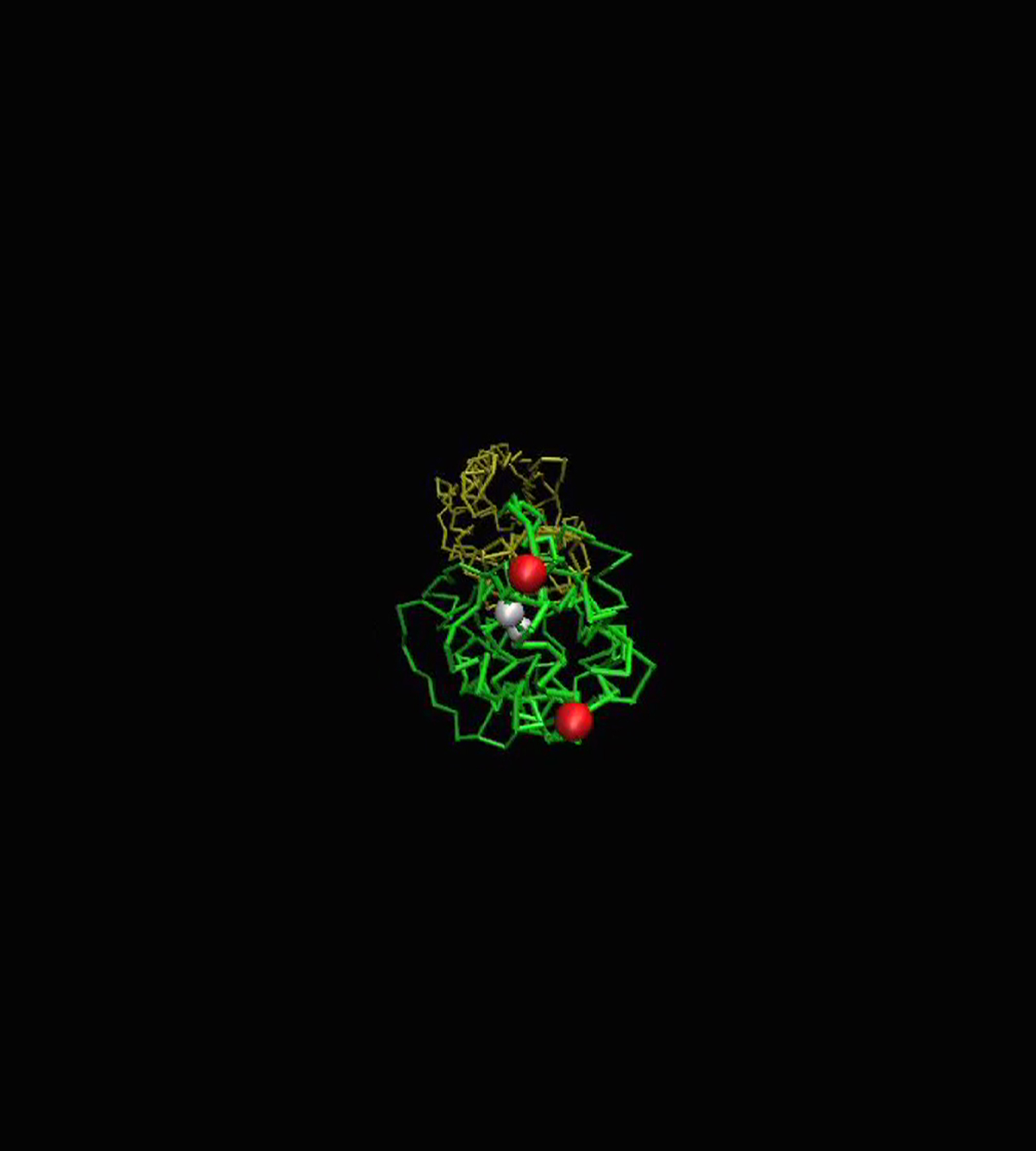

Supplement: Movie S2. PDB: 4FHR PC3 Rotary Motion: En-Face View, Related to Figure 3C [file mmc3.jpg]

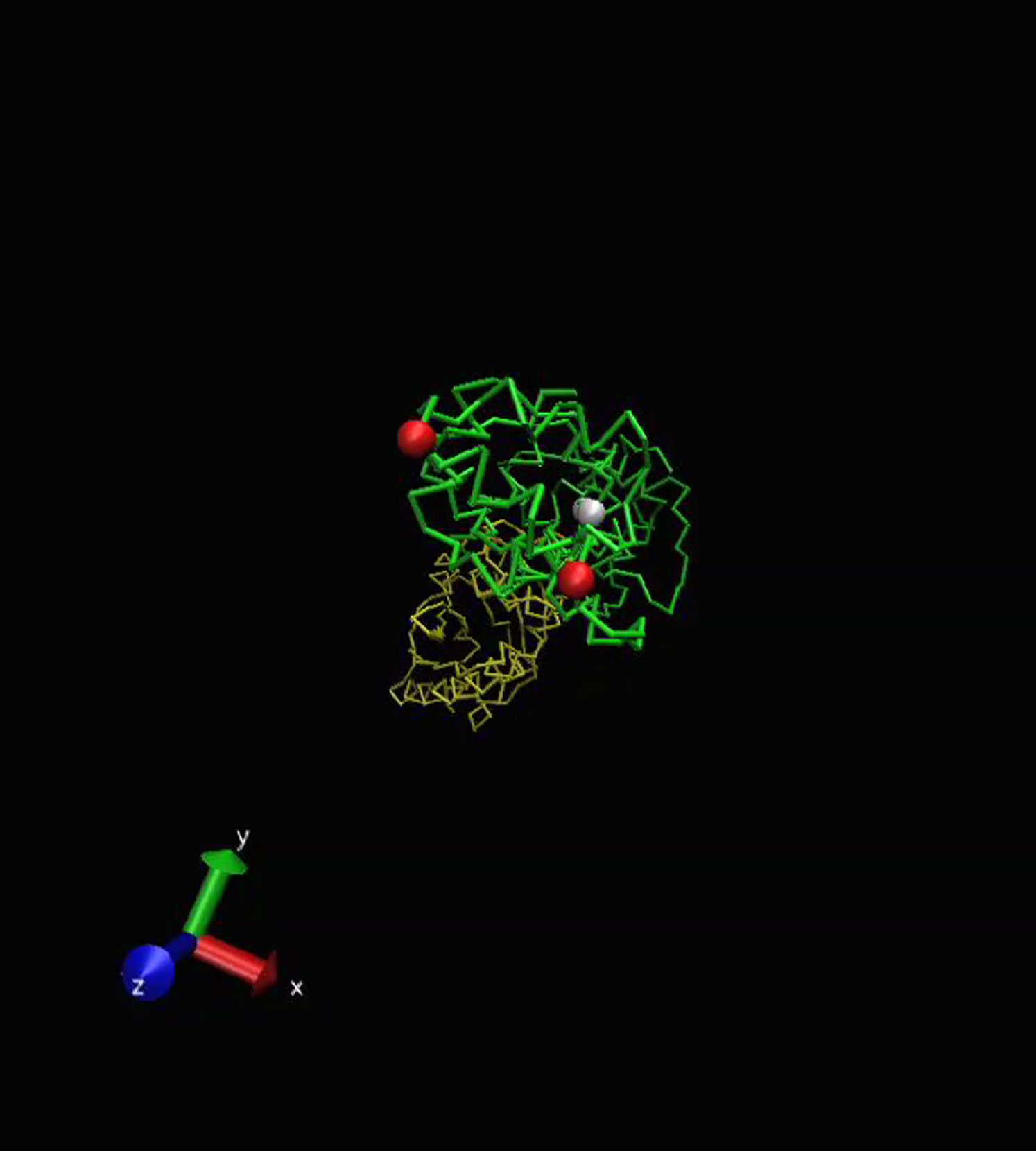

Supplement: Movie S3. PDB: 4FHR PEV Deletion PC3 Rotary Motion: En-Face View, Related to Figure 6A [file mmc4.jpg]

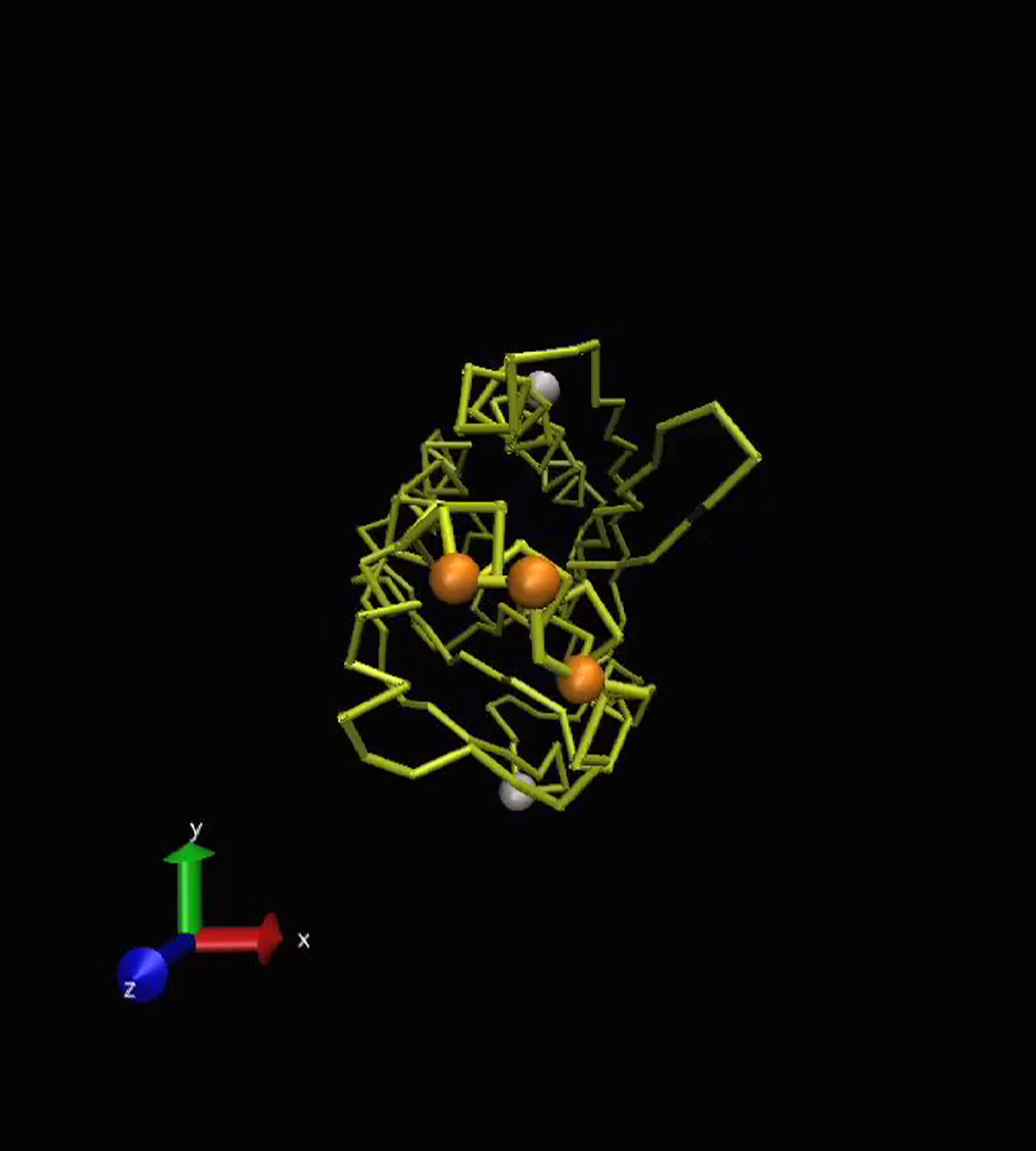

Supplement: Movie S4. PDB: 2HP7 Rotary Motion, En-Face View: First Half, PC1 and Second Half, PC Average, Related to Figure 7D [file mmc5.jpg]

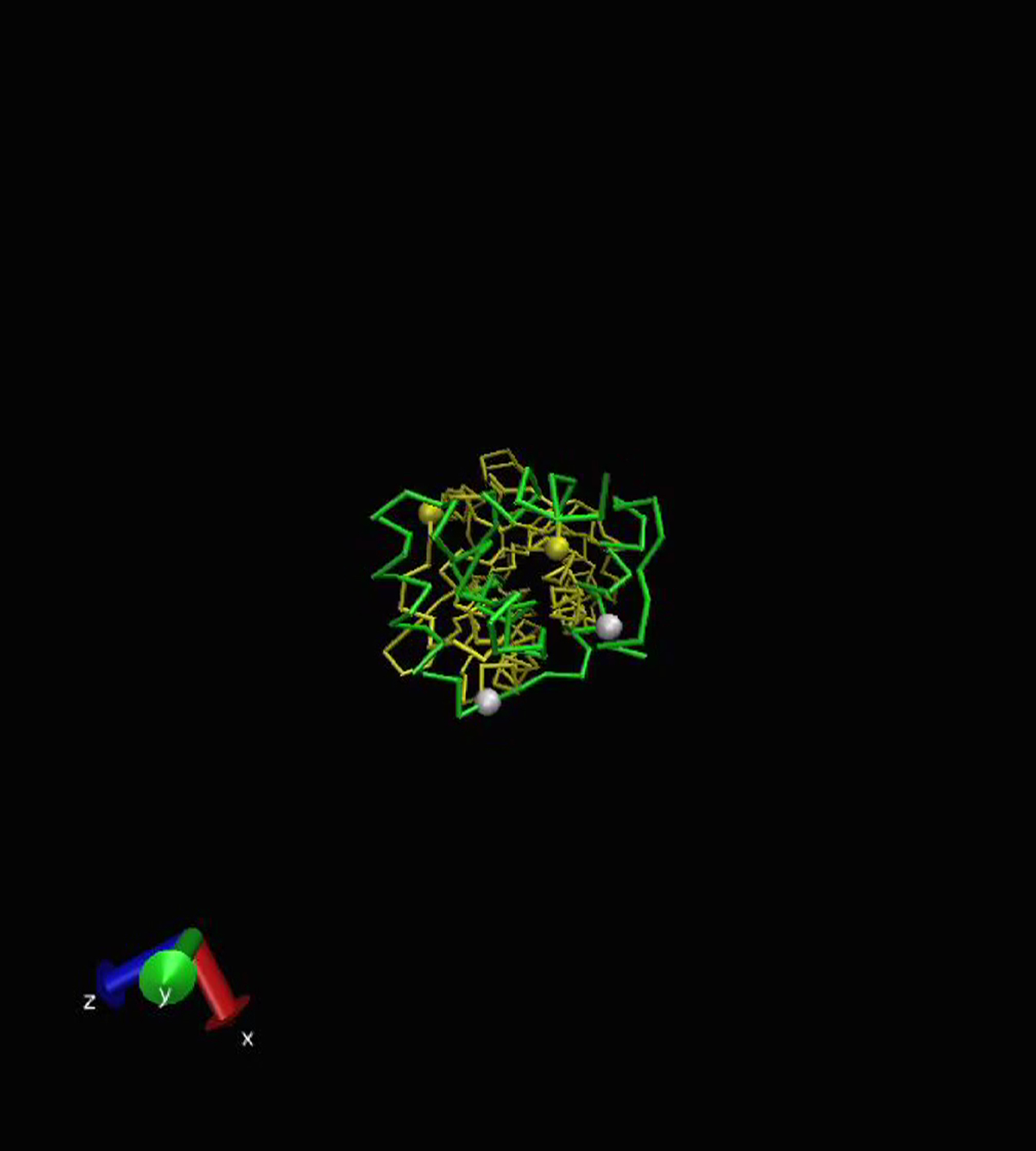

Supplement: Movie S5. PDB: 3SOH Rotary Motion, En-Face View: First Half, PC1 and Second Half, PC Average, Related to Figure 7D [file mmc6.jpg]

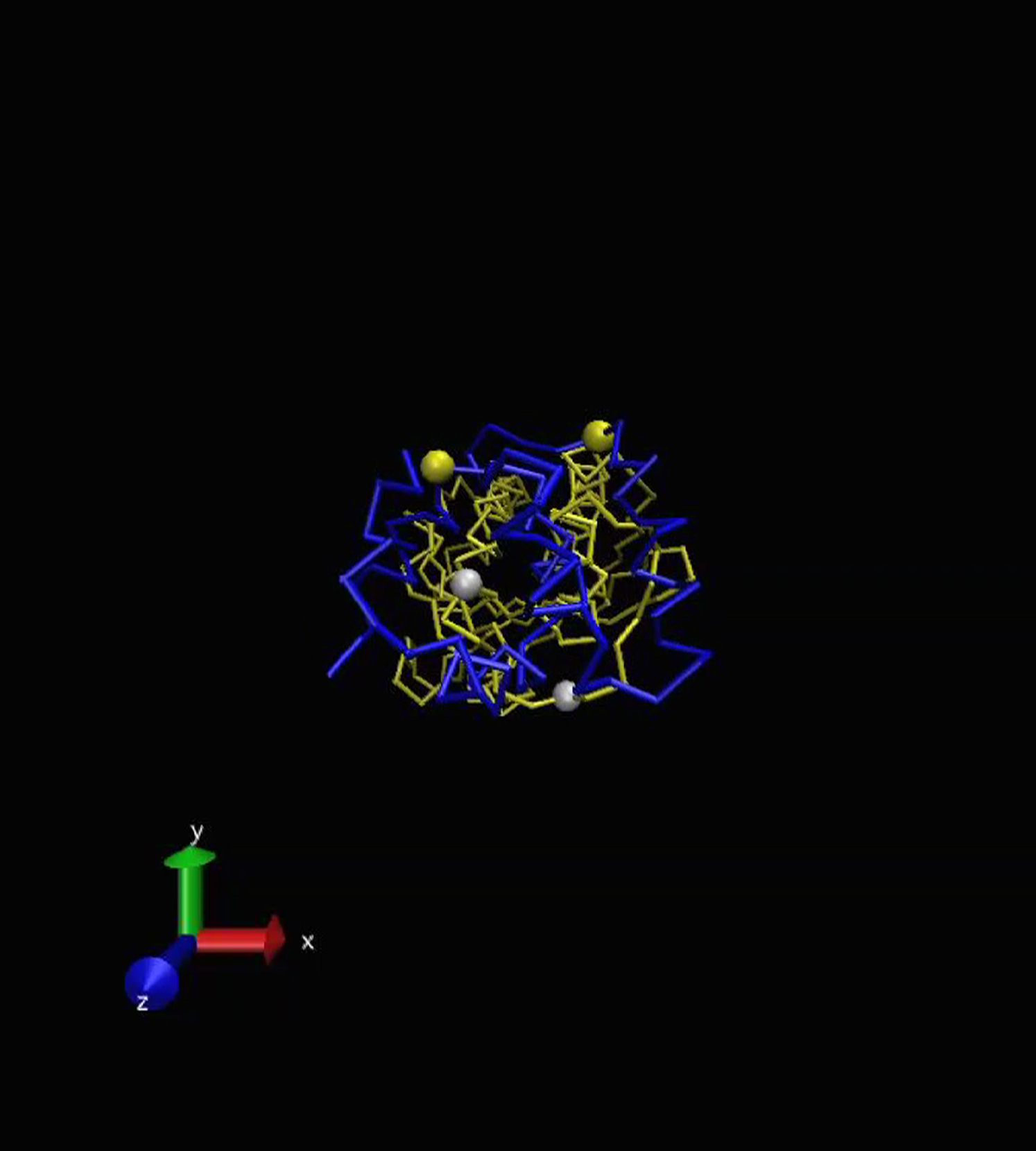

Supplement: Movie S6. PDB: 4FQ0 Rotary Motion, En-Face View: First Half, PC1 and Second Half, PC Average, Related to Figure 7D [file mmc7.jpg]

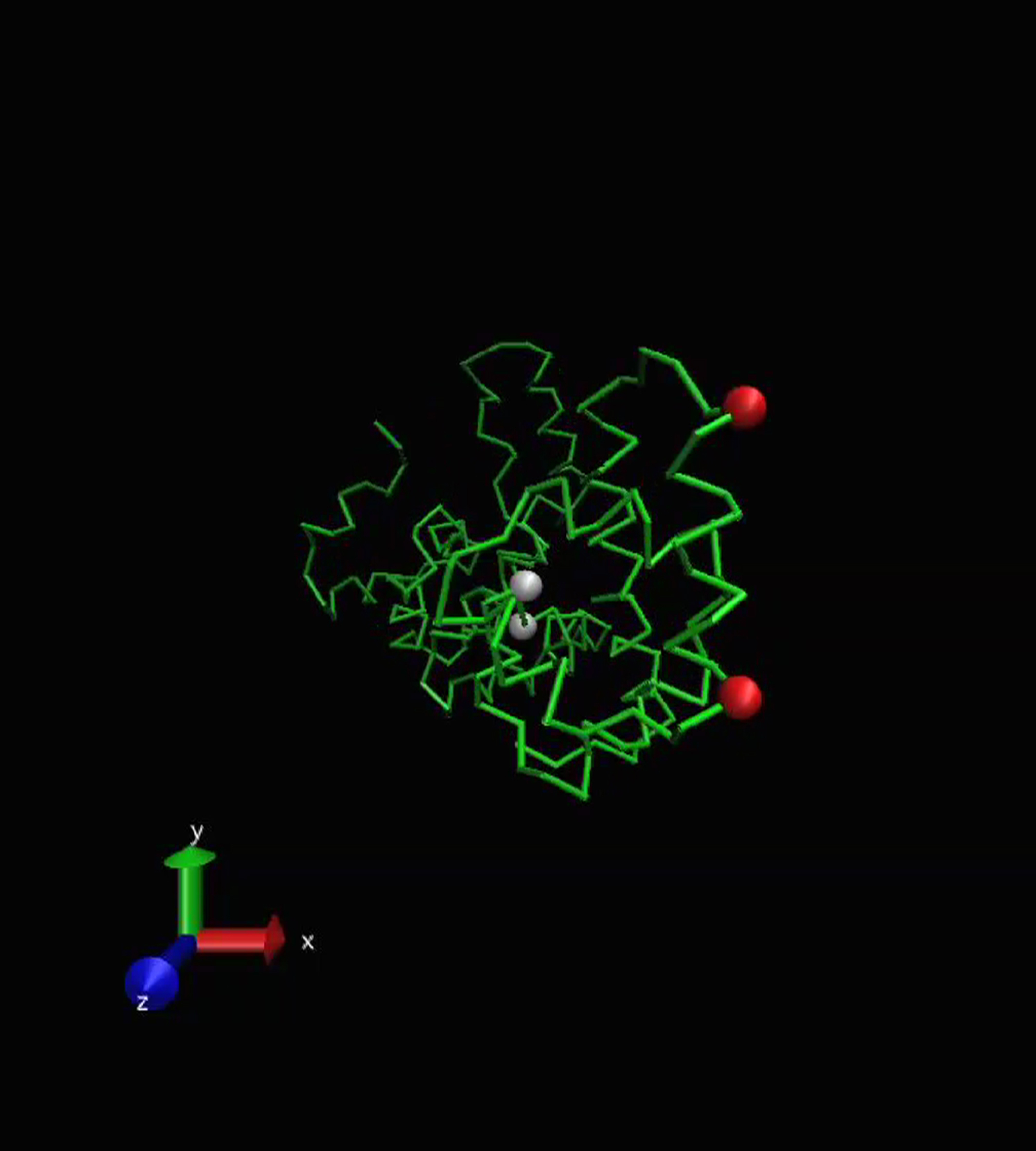

Supplement: Movie S7. PDB: 3AJC Rotary Motion, En-Face View: First Half, PC1 and Second Half, PC Average, Related to Figure 8F [file mmc8.jpg]

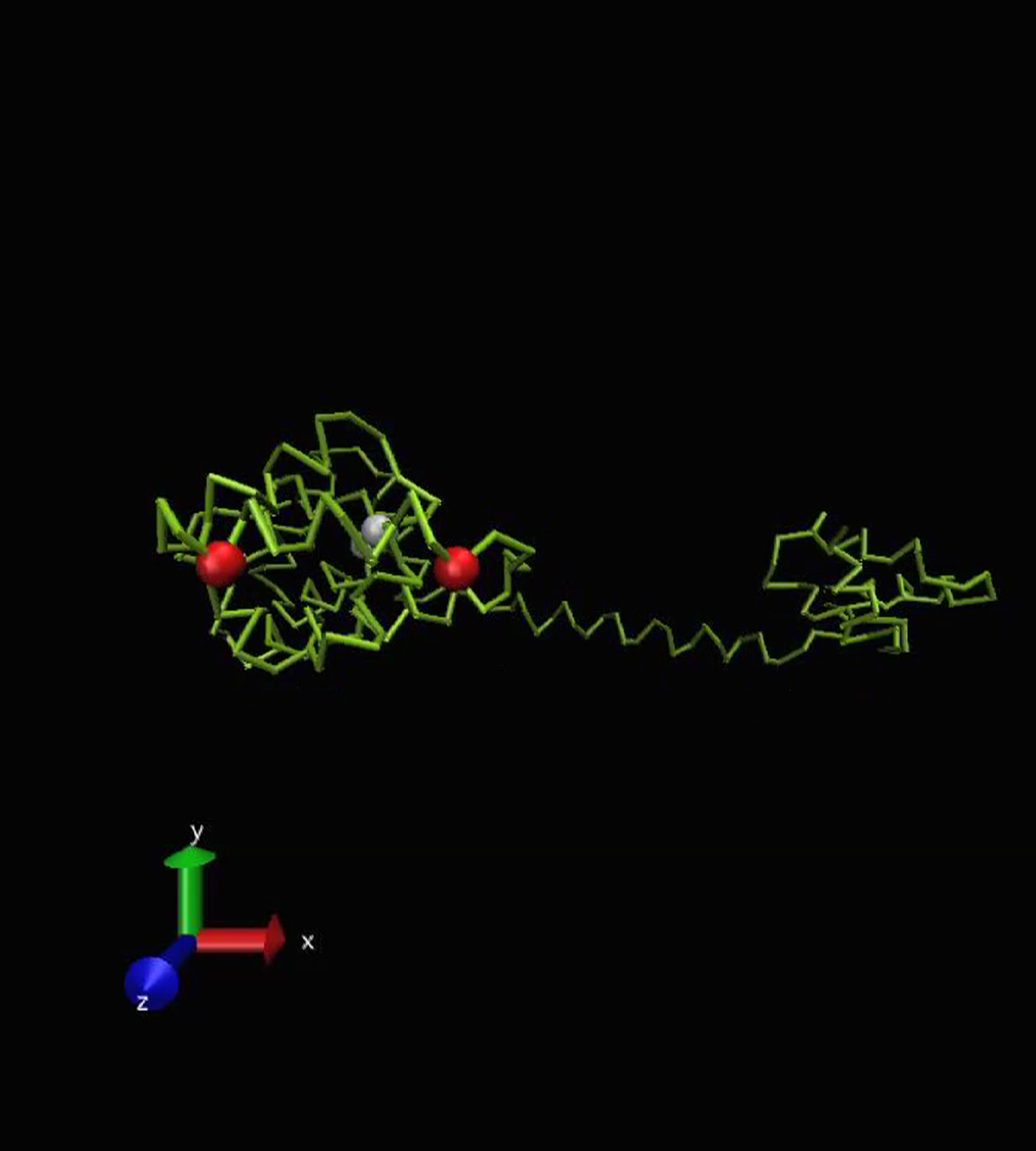

Supplement: Movie S8. PDB: 3USW Rotary Motion, En-Face View: First Half, PC1 and Second Half, PC Average, Related to Figure 8F [file mmc9.jpg]
