# Supplementary material for: The Gearbox of the Bacterial Flagellar Motor Switch
Source: Structure. 2016 Jul 6;24(7):1209–20. doi: 10.1016/j.str.2016.05.012 (PMC4938800; doi:10.1016/j.str.2016.05.012)
Supplement: Document S2. Article plus Supplemental Information [file mmc14.pdf]

Theory

Structure

The Gearbox of the Bacterial Flagellar Motor Switch

Graphical Abstract

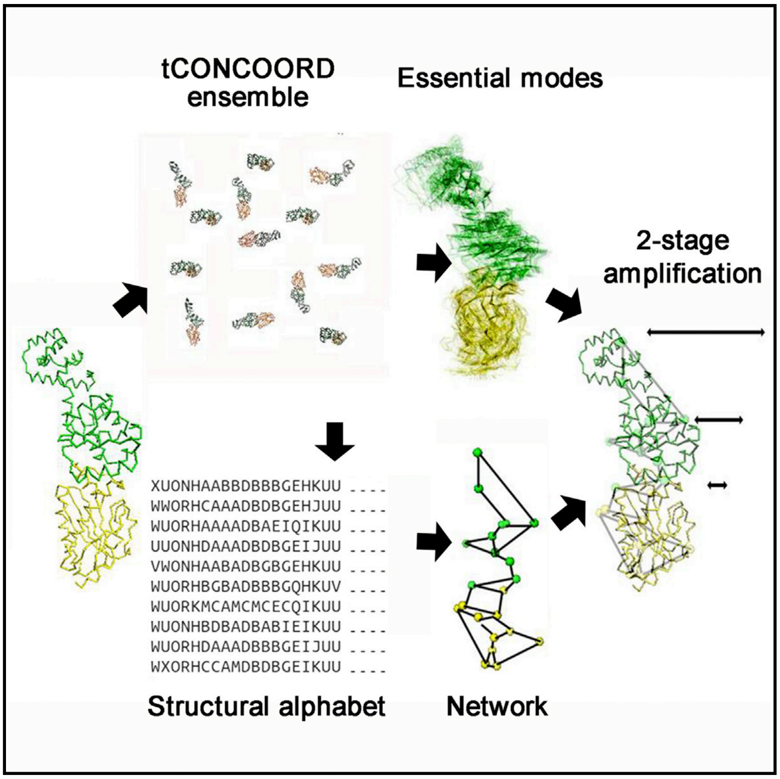

Highlights

- Switch complex exploits differential subunit stiffness for mechanical amplification
- Distinct rotor protein X-ray structures generate overlapping conformer ensembles
- Stacking constraints on a flexible helix linker could select diverse rotation states
- Non-contact elastic couplings at the subunit interface in the complex have coevolved

Authors

Alessandro Pandini, Faruck Morcos, Shahid Khan

Correspondence

khan@mbc-als.org

In Brief

Pandini et al. analyze X-ray structures to chart mechanical signal transmission in a flagellar motor switch complex. Conformational ensembles reveal an evolutionarily conserved interface, two-stage amplification of elastic fluctuations, and conformational selection to explain the fast switching kinetics and provide insight into the allosteric mechanism.

Pandini et al., 2016, Structure *24*, 1209–1220
July 6, 2016 © 2016 The Authors. Published by Elsevier Ltd.
http://dx.doi.org/10.1016/j.str.2016.05.012

CellPress

The Gearbox of the Bacterial Flagellar Motor Switch

Alessandro Pandini,[1,2] Faruck Morcos,[3] and Shahid Khan[4,*]
[1]Department of Computer Science and Synthetic Biology Theme, Brunel University London, Uxbridge UB8 3PH, UK
[2]Computational Cell and Molecular Biology, The Francis Crick Institute, London NW1 1AT, UK
[3]Department of Biological Sciences, University of Texas at Dallas, Richardson, TX 75080, USA
[4]Molecular Biology Consortium, Lawrence Berkeley National Laboratory, Berkeley, CA 94720, USA
*Correspondence: khan@mbc-als.org
http://dx.doi.org/10.1016/j.str.2016.05.012

SUMMARY

Switching of flagellar motor rotation sense dictates bacterial chemotaxis. Multi-subunit FliM-FliG rotor rings couple signal protein binding in FliM with reversal of a distant FliG C-terminal ($FliG_C$) helix involved in stator contacts. Subunit dynamics were examined in conformer ensembles generated by molecular simulations from the X-ray structures. Principal component analysis extracted collective motions. Interfacial loop immobilization by complex formation coupled elastic fluctuations of the FliM middle ($FliM_M$) and FliG middle ($FliG_M$) domains. Coevolved mutations captured interfacial dynamics as well as contacts. $FliG_M$ rotation was amplified via two central hinges to the $FliG_C$ helix. Intrinsic flexibility, reported by the $FliG_{MC}$ ensembles, reconciled conformers with opposite $FliG_C$ helix orientations. FliG domain stacking deformed the inter-domain linker and reduced flexibility; but conformational changes were not triggered by engineered linker deletions that cause a rotation-locked phenotype. These facts suggest that binary rotation states arise from conformational selection by stacking interactions.

INTRODUCTION

The switching of bacterial flagellar rotation provides a remarkable example of a cooperative switch in a large, biomolecular assembly (Bray and Duke, 2004). The assembly, the rotor of the bacterial flagellar motor within the basal body, is composed of about 200 subunits of the component proteins (FliG, FliM, and FliN). These proteins attach to the membrane scaffold formed by FliF subunits forming the C and MS rings (Lux et al., 2000). The interaction of membrane-embedded Mot stator complexes with FliG subunits couples proton transfer to torque generation (Zhou et al., 1998). Chemotactic stimuli change the association of the CheY signal protein with the distal $FliM_{NC}$FliN C ring (Dyer et al., 2009; Sarkar et al., 2010). Coupled conformational transitions in $FliM_M$ (Sircar et al., 2015) trigger large displacements of a distant α helix in FliG, involved in stator contacts (Lam et al., 2012; Paul et al., 2011), henceforth designated toque helix (TH). The chemotactic motor output is a changed clockwise (CW)/counter-clockwise (CCW) rotation bias. CW and CCW intervals have second lifetimes, but switch within milliseconds, mostly with no detectible change in rotation speed (Bai et al., 2013; Lele and Berg, 2015). Absence of intermediate states implies cooperative switching of the multiple subunits (Ma et al., 2012). Activated CheY elicits an "ultra-sensitive" (H = 21) change in CW/CCW bias (Yuan and Berg, 2013), but its binding to motors in situ or rotor assemblies in vitro is not cooperative (Sagi et al., 2003; Sourjik and Berg, 2002). Thus, cooperativity must arise from mechanical amplification within the rotor.

Genetic and biochemical studies on the enteric bacteria *Escherichia coli* and *Salmonella enterica* serovar ("*Salmonella*") provide the paradigm for energization and switching of motor rotation. Non-motile, flagellate (*mot*) and non-chemotactic (*che*) mutations are found in all three proteins. The TH is targeted by *mot* mutations (Lloyd and Blair, 1997). The majority of *che* mutations are in FliM (Magariyama et al., 1990), FliG $helix_{MC}$, and GG loop (Figure 2 of Brown et al., 2002). Other conserved loop motifs (GGXG in $FliM_M$, EHPQ in $FliG_M$, MFXF in $FliG_C$ (letter = conserved residue; X = variable residue), are also targeted by *che* mutations. Figure 1 shows the surmised location of one of ~35 copies of the most complete X-ray structure (*T. maritima* $FliM_M$$FliG_{MC}$ [Vartanian et al., 2012]) in the *Salmonella* basal body. $FliM_M$, a dedicated switch module, is a pseudo-symmetric $\alpha/\beta/\alpha$ sandwich with CW and CCW *che* mutations localized to distinct surface patches (Park et al., 2006). $FliG_{MC}$ has multiple armadillo (ARM) domains; an architectural design that characterizes the entire protein (Lee et al., 2010). The $FliG_C$ C-terminal six-helix bundle (C1-6) contains the TH, forming the motor module.

Here we study the X-ray structures (noted by PDB IDs) to understand the conformational coupling between the switch and motor modules. The available FliG and FliM X-ray structure library is marked by conformational heterogeneity, exemplified by two $FliG_{MC}$ *Helicobacter pylori* structures with opposite (180°) $FliG_C$ C1-6 orientations relative to its N-terminal ARM-C (Lam et al., 2012), that has engendered a lively debate (Stock et al., 2012). The heterogeneity could arise because component subunits have discrete states trapped in different minima in the energy landscape; analogous to the open and closed states of sugar binding proteins (Morcos et al., 2013). Alternatively, it could be due to intrinsic flexibility, with the two rotation states generated by conformational selection as found for binding of ADP to the F_0F_1 ATP synthase (Czub and Grubmuller, 2014). We used tCONCOORD to discriminate between these

CrossMark

This is an open access article under the CC BY license (http://creativecommons.org/licenses/by/4.0/).

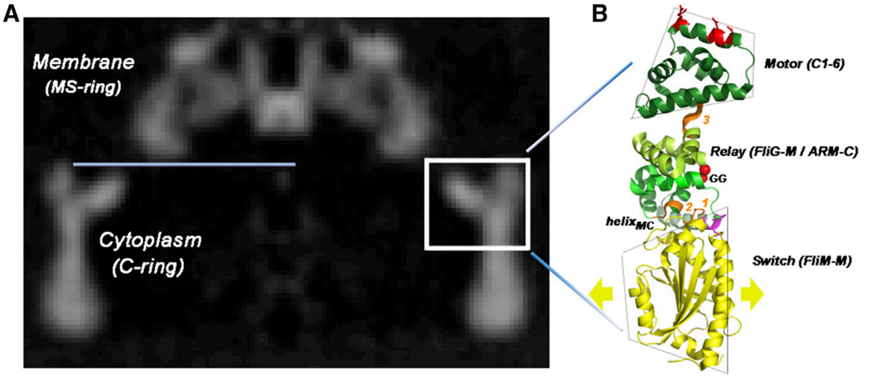

Figure 1. The *Salmonella* Basal Body MSC Ring and the *T. maritima* Proximal Switch Complex

(A) A cross-section through the *Salmonella* flagellar basal body electron microscopy reconstruction (Thomas et al., 2006) showing transmembrane MS ring and the cytoplasmic C ring. Blue line marks membrane cytoplasm boundary. Box marks the surmised location of FliG and associated $FliM_M$. The FliG ring interacts with transmembrane Mot stator complexes. $FliM_M$ reports CheY binding to FliG and adjacent FliM subunits (gold arrows).

(B) The atomic structure (PDB: 4FHR) of the *T. maritima* complex: $FliM_M$ (gold), $FliG_M$ (green), FliGc ARM-C (olive), C1-6 (dark green), MFVF motif (orange), and TH (red side chains). Deletion of three *Salmonella* residues homologous to *T. maritima* PEV (magenta) produces a CW biased phenotype. See also Figure S1 for secondary structures and the contact interface.

alternatives. tCONCOORD generates atomic-detail conformational ensembles from a single structure based on distance constraints (de Groot et al., 1997). Detection of labile hydrogen bonds facilitates conformational transitions (Fernandez and Scheraga, 2003; Seeliger et al., 2007). Collective motions were extracted from principal component analysis (PCA) (Amadei et al., 1993) of the ensembles. The dynamics of successive four-residue fragments in conformers encoded as a set of strings with a structural alphabet (SA) (Pandini et al., 2010) unveiled the local motions generating collective modes. Network analysis related interfacial dynamics and coevolution; an important issue for protein machines being addressed by various groups (Morcos et al., 2013; Sfriso et al., 2016; Sutto et al., 2015). Finally, we engineered a three-residue FliG linker $helix_{MC}$ deletion in all structures to assess whether it triggers conversion to the stacked conformation observed for the deletion protein X-ray structure.

The $FliM_M$ structure ensembles reveal a stiff domain that fluctuates between two states. The $FliG_{MC}$ structure ensembles, irrespective of species, sample a broad conformer space that is constrained by FliG ARM domain stacking. Residue coevolution identifies both $FliG_MFliG_{MC}$ interfacial contact and elastic couplings. Complex formation couples $FliM_M$ fluctuations to FlG_M rotation, amplified via two central hinges to a large angular reorientation of $FliG_C$ C1-6. The design allows rapid reorientation of $FliG_C$ C1-6 upon altered tilt of the more rigid $FliM_M$ ring within the basal body. $Helix_{MC}$ architecture is too pliable for deletions within it to trigger ARM domain stacking. Instead, the stacking could select alternative rotation states from a broad conformational spectrum.

RESULTS

Our analysis of the tCONCOORD conformational ensembles had two stages. First, we examined the ensemble from the $FliM_MFliG_{MC}$ complex. Anharmonic collective motions were identified by PCA of residue C_α position fluctuations and the principal components (PCs) mapped onto the structure. Conformational dynamics of SA-encoded fragments were correlated with the PC motions and each other for characterization of the mechanical network, its relation to interface coevolution, and perturbation by engineered CW-locked deletion mimics. Second, we applied the methodology to the complete structure library of the component $FliM_M$ domains, $FliG_{MC}$ and $FliM_MFliG_M$ complexes. The comparative analysis assessed the effects of complex formation on the individual components, determined a common mechanical design, and evaluated the species-dependent contribution to the variability.

The *T. maritima* $FliM_MFliG_{MC}$ Conformer Ensemble Records Large $FliG_c$ Motor Domain Movements

Computed residue temperature factors (B factors) for the $FliM_M$ $FliG_{MC}$ ensemble were compared with experimental values (Figure 2A). The simulated $FliM_M$ B-factor profile was in reasonable agreement with the crystallographic factors. In contrast, the match was poor for $FliG_{MC}$. The dominant peak in the simulated profile, at the TH, was damped in the experimental profile. The most prominent peak in the experimental profile bordered the missing seven-residue segment ($V_{188}SRTFSK_{194}$) adjacent to the $G_{196}G_{197}$, grafted in from another *T. maritima* structure (PDB: 1LKV). The second peak was centered at ARM-C E_{223}. Downweighted, low-amplitude peaks were obtained at these positions in the simulated profile. Solvent-accessible surface area (SASA) variations within the ensemble (Figure 2B) identified the β1*/H2* loop (E180-P184) as the most variable $FliM_M$ segment. In $FliG_{MC}$, the high B-factor segments (H6/H7 GG, H8/H9 MXVF, and TH N-terminal loops) had the most variable SASA.

PCA Identifies TH Displacements as the Principal Collective Motion

The isotropic motions of an ideal molten globule have a flat PC spectrum with equal amplitude eigenvalues. Secondary structures create hinges and shear planes that coordinate collective, anisotropic movements to generate dominant PC modes. The complete PC spectrum measures overall flexibility. Domains were isolated from the complex in silico to assess the effects of complex formation. The relative PC amplitudes were normalized with respect to the summed amplitude of the $FliM_M$ eigenvalue spectrum (Figure 3A) to show that the intrinsic flexibility of $FliG_{MC}$ was substantially more than that of either the smaller $FliM_M$ or the larger $FliM_MFliG_M$ complex. The variance of the ensemble was largely (~90%) captured by the first three PCs. We plotted cumulative amplitudes to better determine

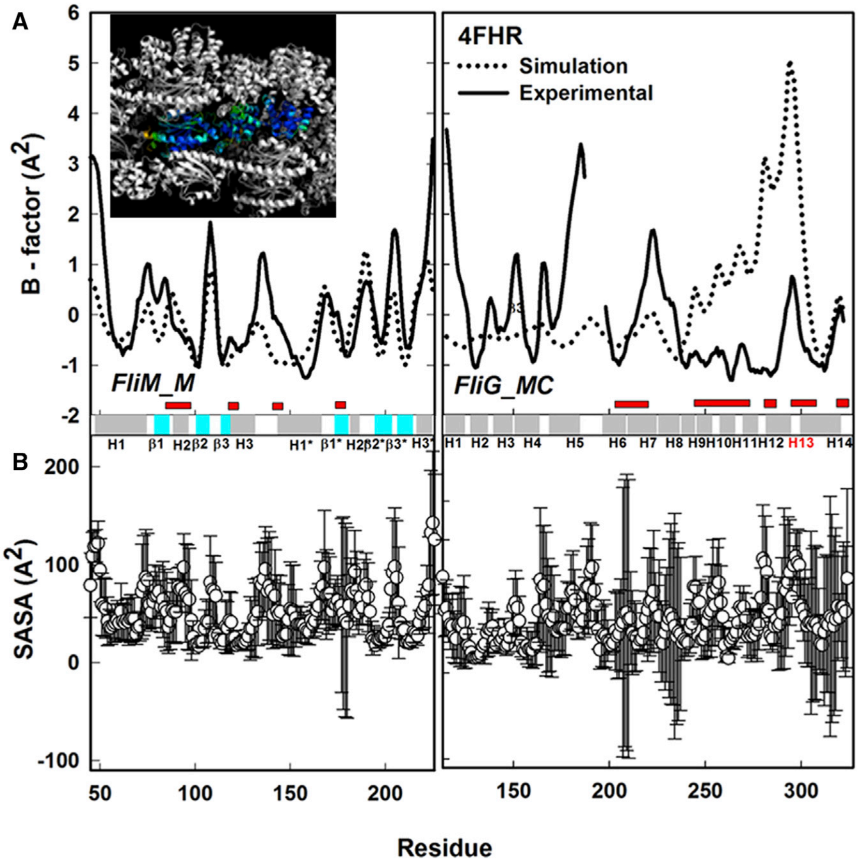

Figure 2. Residue Fluctuations in $FliM_M$ $FliG_{MC}$

(A) Experimental B-factor values (solid line) compared with simulated values (dotted line). The B factors were normalized. $B = ((B_{res} - B_{mean})/\sigma_B)$, where B_{mean} and σ_B are mean and SD, respectively, of the simulated B-factor (B_{res}) distribution. Parameters in later figures are normalized similarly. Red bars denote residues in contact with neighboring complexes in the crystal. $FliM_M$ $P_{corr} = 0.63$. $FliG_{MC}$ P_{corr} = (−0.2 [overall]; 0 [non-contact N-terminal]). Inset: PDB: 4FHR unit cell (B factor high = orange; intermediate = green/cyan); low = blue) and neighboring complexes (white). H13 = TH.

(B) Residue SASA (mean [open circles] ± σ). Horizontal bar shows secondary structure elements: α helices (gray), β sheet (cyan), loops (white).

differences in anisotropy (Figure 3B). The $FliM_M$ PC spectrum becomes more anisotropic upon complex formation with either $FliM_M$ or $FliG_{MC}$. In contrast, $FliG_{MC}$ was not affected by complex formation.

We constructed a mechanical analog to physically map the PC amplitudes onto the structure (Figure 3C). The $FliM_MFliG_{MC}$ complex was represented as a segmented beam. The FliG GG and MXVF loops constituted flexible hinges consistent with the SASA profile, in addition to the subunit interface. Hinge motions were most simply deconvolved into bending and rotary components as measured for another segmented protein, the myosin rod (Highsmith et al., 1982). We marked line vectors within the structure (Figure 3D) and recorded ensemble distributions of angle fluctuations between vector pairs to refine the mechanical model. The SDs (σ) of the angle distributions (Figure 3E) showed that the subunit interface and inter-ARM loops were more flexible relative to other parts of the protein, consistent with the initial model. However, the GGPG loop rotary twist (ML2-MGG) was prominent in $FliM_M$ motions. $FliG_M$, mechanically coupled to $FliM_M$ bends and rotates at the interface (ML0-GL0). C1-6 bending and rotary motions (GL2-GP2) relative to $FliG_M$ (GL1-GP2) are amplified from the interface motions. The overall amplification is 3.8-fold for PC2 and 4.2-fold for PC3. The PC contributions to the TH (GL2-GP2) σ were ±5.4° (PC1)/±9.6° (PC2)/±17.7° (PC3). PC1 (Movie S1) predominantly recorded bending motions at the interface and the MFVF motif, and PC3 (Movie S2) the rotation of FliG C1-6 relative to $FliM_M$.

The Elastic Compliance in the Coupling between $FliM_M$ and FliG C1-6

Two angle distributions for the PC motions were examined in detail. The distribution for the TH PC3 rotation relative to the MFVF hinge is bimodal with the ends more populated than the center (Figure 4A). A two-Gaussian (R^2 = 0.94) fit is also better than a single Gaussian (R^2 = 0.78) for angular displacements measured across the subunit interface. The torsional stiffness estimated from the interface rotation is 740 pNnm (one state) to 1,500 pNnm (two states). C1-6 rotation determined the conformer spread as seen from projection of its angular distribution on the PC1PC3 plane. Similar results were obtained for projection onto the PC2PC3 plane.

The composite PC1 + PC3 rotation at the interface and MFVF hinge had a flat angular distribution with increased spread. The flat distribution resulted from summation of two PCs with different relations for the TH-interface motions (Figure 4B). For PC1, the relation between the interface and TH rotation amplitudes is monotonic. For PC3, there are end states where TH orientation is insensitive to interface motions, separated by a linear (8 ± 1) response. Both relations are distinct from relations between inter-domain bending motions that have a parabolic form consistent with motion in an elastic potential well (Figure 4C). Specifically, interface rotation of the GGPG loop relative to the $FliM_M$ H1/H2 long axis is constrained by H2 displacement from its favored orientation relative to β1-β3 sheets. Rotation of FliG C1-6_c around the MFVF loop is constrained by its bending relative to $FliG_M$. These elastic couplings preserve the protein fold. The C1-6 rotational flexibility at the MFVF hinge (GP2-GL2) from the complete PC has σ = ±28°.

Detailed Analysis of Hinge Elements

SA-encoded fragment motions (Figure S2) characterized local fluctuations (Figure 5A). Fragments from secondary structures in the crystal sampled conformations that preserved type throughout the ensemble. Short loops sampled loop-specific

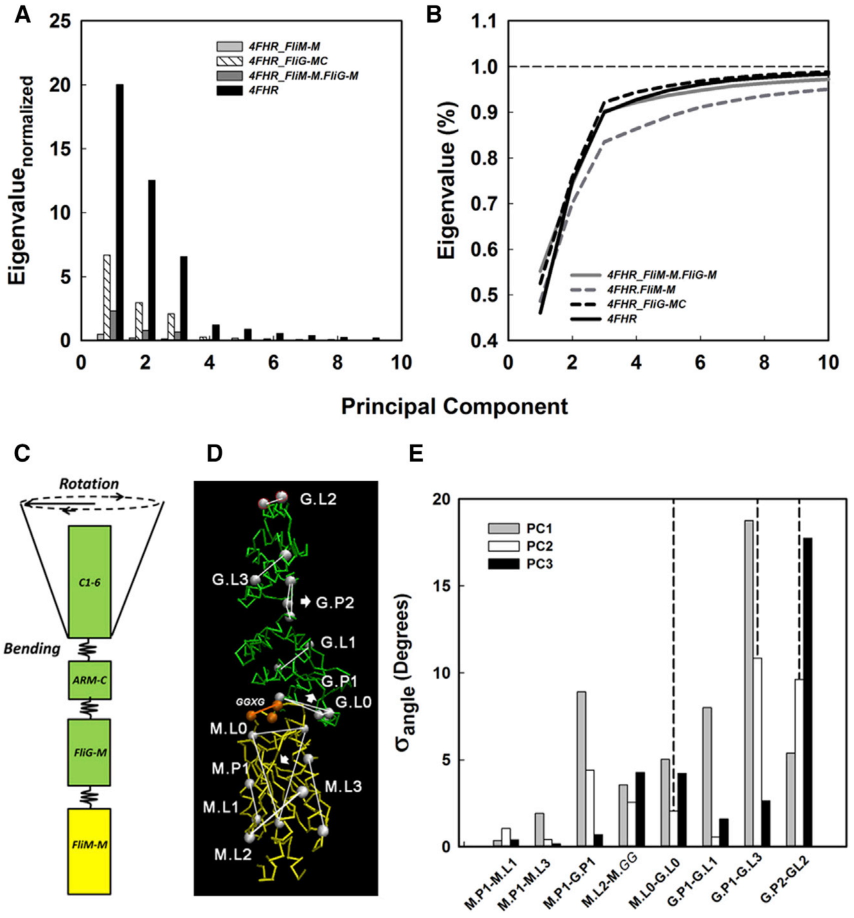

Figure 3. PCA of the FliM$_M$FliG$_{MC}$ Complex

(A) Eigenvalues for the first ten PCs of the ensembles normalized by the sum of the FliM$_M$ (gray) eigenvalues.

(B) Cumulative spectra (lines) show the anisotropy of FliM$_M$ motions is increased in complex with FliG$_M$ (dark gray line) or FliM$_M$FliG$_{MC}$ (black line) than when alone (dashed gray line). The anisotropy of FliM$_{MC}$ (dashed black line) motions is not affected by complex formation.

(C) Schematic of the complex as a segmented rod with intervening hinges. FliM$_M$ (gold), FliG$_{MC}$ (green).

(D) Vectors (white lines) used for measurement of hinge and interfacial motions. White spheres mark C_α atoms of connected residues. White arrows show perpendiculars to the chosen planes (triangles). Orange vector (line) marks GGPG motif (spheres).

(E) First three PC amplitudes between vector pairs measured as the σ of the difference angle distributions. Bending = MP1.ML1, MP1.ML3, MP1. GP1, GP1.GL1, GP1.GL3. Rotation = ML2.MGG, ML0.GL0, GP2.GL2. Dashed line separates FliM$_M$ and FliG$_{MC}$ pairs. Vector labels are as in (D).

conformations; but long loops (e.g., GGPG loop), also sampled β-sheet conformations. The helix$_{MC}$ segment grafted from PDB: 1LKV sampled loop and β-sheet, rather than α-helix, conformations.

Local fragment fluctuations were correlated with trajectory displacements along the PCs (Figure 5B). Hinges, defined as segments with high nMI$_{PC}$ contribution, included both static (low root-mean-square fluctuation [RMSF]) and dynamic (high RMSF) elements. Within FliM$_M$, the prominent static hinges were loops between H2/β2, H1*/β1* (PC1), H1/β1, H2*/β2* (PC2), and H2*/β2*, β2*/β3* (PC3). For all three PCs, the long FliM$_M$ GGPG loop was a dynamic hinge. The FliM$_M$ HI long helix central segment, enriched in polar residues and thus susceptible to hydrolysis, was the second dynamic hinge (PC3). Within FliG$_{MC}$, the N-terminal helix$_{MC}$ loop was a hinge for PC1 and PC3; while the EHPQ, GG, and MFVF motif loops formed additional PC3 hinges. For all three PCs, the RMSF profile peaked at or adjacent to the TH, accounting for the B-factor profile (Figure 2A).

In conclusion, the premise for the segmented beam model is validated, but supplemented with knowledge of the inherent elasticity of the FliM$_M$ and FliG$_M$ segments.

The Mechanical Network between FliM and FliG

We constructed FliM$_M$ and FliG$_{MC}$ centrality profiles from the covariance matrix of the encoded fragment correlations to measure the contribution of each fragment to the network of local motions (Figures 6A and 6B). The entropy profile identified flexible loops. The FliM$_M$ and FliG$_M$ loops form a distributed hinge system of network nodes in the composite profile. The remaining two nodes localized to the MFVF motif and the central C1-6 helix. The C1-6 loops inter-helix loops did not influence the network. The profile peaks represented the major nodes: nine for FliM$_M$ and ten for FliG$_{MC}$. The profiles superimposed with the composite nMI$_{PC}$ profile for the PC1-PC3 motions (P_{corr} > 0.9). Thus, the PC1-PC3 motions are the dominant output of the mechanical network.

Three helix$_{MC}$ residues (PEV) homologous to the CW-locked *Salmonella* deletion (PAA) form its N terminus close to the interface. Their deletion reduced FliG$_M$ intra-domain contacts as well as contacts with FliM$_M$ (Figure S4). However, long-range couplings between FliM$_M$ and loops adjacent to the FliG TH persisted and collective motions were largely unaltered (Movie S3), highlighting the robust nature of the distributed system.

Structural maps of the top nMI$_{local}$ correlations (Figure 6C) revealed the mechanical relay between FliM$_M$ and FliG$_C$. FliM$_M$, with its β-sheet center as pivot, connects to FliG$_M$ core helices H2 and H4. Comparison of the FliMFliG interface residue coevolution and dynamics (Figure S3) showed that, in addition to interface contacts, nodes of the coevolved network overlap/flank long-range dynamic network nodes. The overlap is evident in the structural maps of the communication pathways. However, coevolution only reports some β-sheet motions important for interface dynamics, for reasons not presently understood. FliG$_M$ also has a dense network built around its core helices with sparse connectivity to C1-6. The network centrality and spatial architecture are consistent with the idea that mechanical transmission may be conceptually divided into two

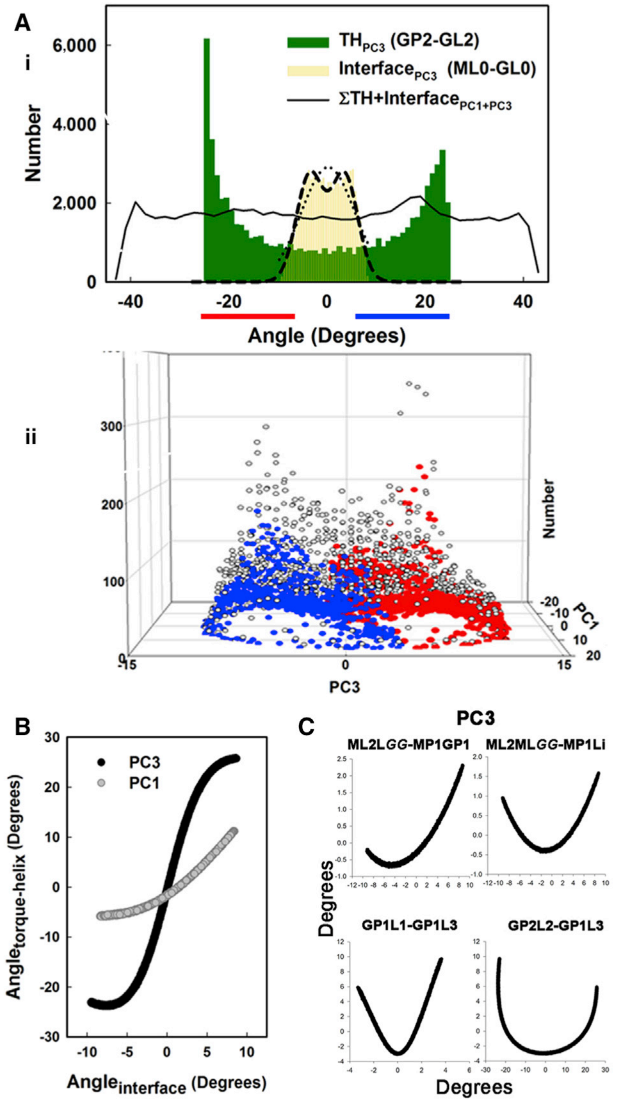

Figure 4. Two-Stage Rotary Amplification

(A) (i) PC3 rotation angle distributions. Horizontal bars (red [<5°]/blue symbols [>5°]) denote subpopulations of the TH (GL2-GP2) distribution. Fits to the FliMFliG interface (ML0-GL0) distribution are single Gaussian ($y = a*\exp(-0.5((x-x0)/b)^2)$), where a = 2,904, b = 5.48, x0 = 0.2 (dotted line) and double Gaussian ($a*\exp(-0.5((x-x0)/b)^2)) + (a*\exp(-0.5((x+x0)/b)^2)$), where a = 2,701, b = 2.72, x0 = 3.55 (dashed line). Combined PC1 + PC3 angle distribution ($\sigma = \pm 13.0°$) for both vector pairs (line). (ii) The subpopulations of the TH distribution partition to opposing ends of the PC1PC3 plot, showing that conformer spread (open symbols/gray edges) tracks TH motions.

(B) The coupling between interface and TH motions.

(C) Elastic coupling (PC3 rotation) between other elements in the complex. Vectors are as in Figure 3C. Mean orientations (0°) in (B) and (C) are for the ensemble-averaged structure.

stages: mechanical coupling at the interface that transmits fluctuations of the stiff $FliM_M$ domain to $FliG_M$; with subsequent transmission via hinge motions to effect C1-6 rotation.

Comparative Analysis of Component Structures

X-Ray structures of component proteins and partial complexes were superimposed with the reference PDB: 4FHR structure on a domain-by-domain basis based on common residue positions. Superposition of these static structures did not reveal differences between the species (Figure S5). We proceeded with analysis of the conformer ensembles.

The $FliM_M$ GPGG Loop Is Immobilized by Complex Formation

The PC1PC2 plots for the $FliM_M$ monomers superimposed with the plot for PDB: 4FHR (Figure 7A). The $FliM_M$ plot of the *H. pylori* $FliM_MFliG_M$ complex (PDB: 4FQ0) was displaced, albeit of similar form, from the other plots. The PC1-PC3 σ of the ensembles ranged from 0.628 ± 0.002 (PDB: 2HP7) to 0.448 ± 0.002 (PDB: 3SOH). The overlap showed that species differences and subsequent rearrangements of the stiff domain upon complex formation were small.

We correlated fragment dynamics with the global PC motions as for the PDB: 4FHR $FliM_M$ dynamic network (Figure 7B). The nMI_{PC1} profiles of the $FliM_M$ monomers and the $FliM_MFliG_M$ complexes followed the PDB: 4FHR profile (Figure 5B) (average $P_{corr} = 0.49 \pm 0.03$) (Figure 7C), with some differences. The $FliM_M$ monomer profiles lacked the $\beta2/\beta3$ loop network node. In addition, the contribution of the central GGPG node was reduced in the *T. maritima* monomer (PDB: 2HP7). The dominant node for $FliM_MFliG_M$ profiles, as in PDB: 4FHR, was N-terminal $helix_{MC}$ interfacial loop. In the *H. pylori* PDB: 4FQ0 profile the $FliM_M$ N-terminal H1 and the C-terminal $\beta2^*/\beta3^*$ loop were more prominent, the FliG H1/H2 EHPQ motif loop less so. The PDB: 4FHR profile agreed more with the profiles of the complexes rather than the monomers.

Rotary twist of the GGPG loop relative to the central long axis was the principal (PC1) motion in the $FliM_M$ monomers (*T. maritima* PDB: 2HP7 [$\sigma = \pm 14°$] [Movie S4], *H. pylori* PDB: 4GC8 [$\sigma = \pm 11°$]). These motions exceeded the combined PC1-PC3 PDB: 4FHR $FliM_M$ motion. The interfacial rotation of $FliG_M$ relative to $FliM_M$ was the principal PC1 motion in $FliM_MFliG_M$ complexes (Movies S5 and S6); while bending dominated the PC2 and PC3 motions (Figure 7C). We conclude that species interface dynamics vary in degree, not strategy; with twist of the GGPG loop, the dominant intrinsic motion of $FliM_M$, harnessed upon complex formation to drive $FliG_M$ rotation. Other features of the dynamic $FliM_M$ network are conserved across all ensembles.

Two Hinges Determine $FliG_{MC}$ Flexibility

The modulation of intrinsic $FliG_{MC}$ flexibility by complex formation was determined similarly. The first three PCs were projected onto 2D planes (Figures 8A and 8B). All $FliG_{MC}$ ensembles had greater spread (PC1-PC3 σ [nm]) than the $FliM_M$ ensembles. The ensembles were resolved into two sets based on overlap and spread (PC1-PC3 σ). The overlapping *T. maritima* PDB: 1LKV (2.04 ± 0.006), *Aquifex aeolicus* PDB: 3HJL (2.09 ± 0.006), and *H. pylori* PDB: 3USY (2.655 ± 0.006) and 3USW (3.86 ± 0.014) ensembles formed one set separate from the *T. maritima* PDB: 3AJC (1.36 ± 0.004) and 4FHR (2.42 ± 0.008) ensembles. The latter structures have $FliG_M$/ARM-C stacking interactions.

Correlations of the ensemble nMI_{PC1} profiles with PDB: 4FHR (Figure 8C) were worse ($P_{corr} = 0.16 \pm 0.06$) than for $FliM_M$, consistent with greater conformational variability. The

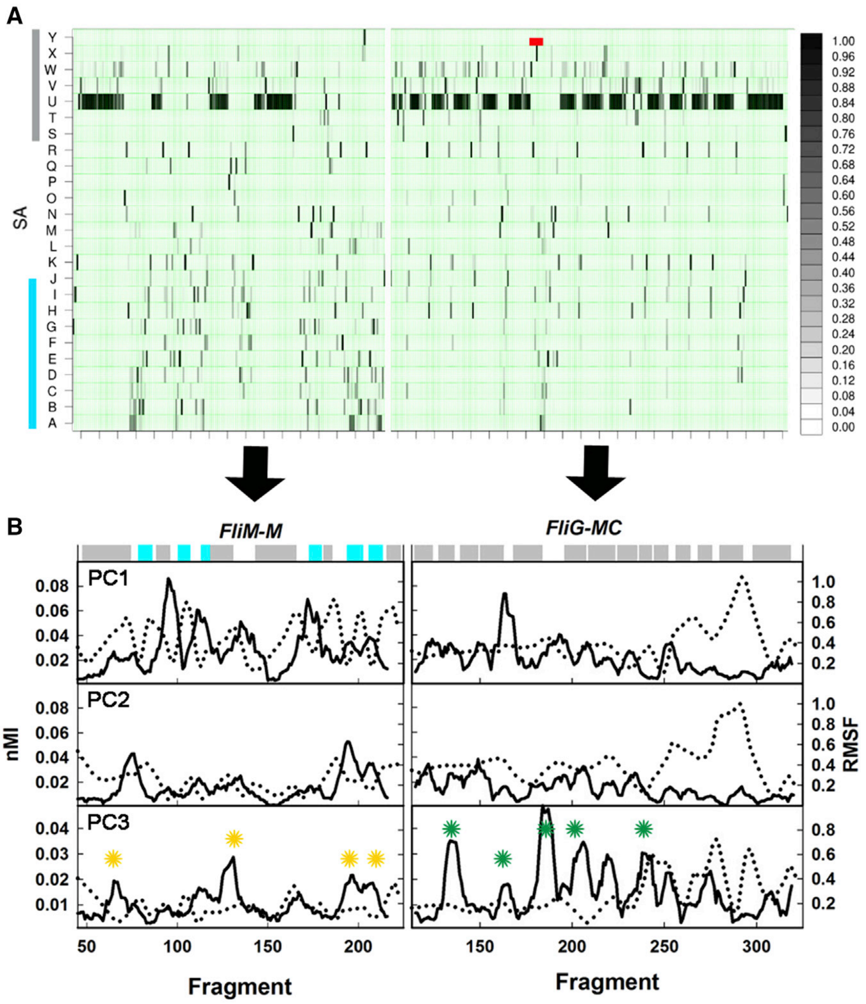

Figure 5. Hinge Elements for the PC Motions

(A) SA representation of ensemble fluctuations. SA letters readout of secondary structure (α helix [gray bar], β sheet [blue bar]) as detailed (Figure S2). Red bar denotes grafted PDB: 1LKV helix$_{MC}$ segment. Grayscale bar (black, high; white, low) denotes populated ensemble fraction.

(B) Superimposed nMI$_{PC}$ (black lines) and RMSF (dotted lines) profiles for PDB: 4FHR. Horizontal bar, colors as in (A), shows secondary structure profile. Peaks that represent hinges for the PC3 rotation in FliM$_M$ (yellow asterisks) and FliG$_{MC}$ (green asterisks) are marked. For FliM$_M$ RMSF-nMI$_{PC1}$ $P_{corr} = -0.17$, RMSF-nMI$_{PC3}$ $P_{corr} = -0.2$, nMI$_{PC1}$-nMI$_{PC3}$ $P_{corr} = 0.27$. For FliG$_{MC}$, RMSF-nMI$_{PC1}$ $P_{corr} = -0.36$, RMSF-nMI$_{PC3}$ $P_{corr} = -0.21$, nMI$_{PC1}$-nMI$_{PC3}$ $P_{corr} = 0.21$.

See also Figure S2.

nMI$_{PC1}$ profiles of all ensembles were merged to detect common nodes (Figure 8D). The interface (EHPQ and N-terminal helix$_{MC}$) loops were not prominent in the FliG$_{MC}$ networks, showing that their PDB: 4FHR network centrality was due to complex formation. The GG and the MFVF loop formed the dominant nodes, with 3-fold greater amplitude than the next prominent node (TH C-terminal loop). This result extends the PDB: 4FHR segmented beam model to all FliG$_{MC}$ structures.

The Effects of Domain Stacking and FliM$_M$ Complex Formation on FliG$_{MC}$ Flexibility

We reasoned that FliG$_{MC}$ conformer ensembles are best compared by motions around the two central hinges. As for PDB: 4FHR, we generated ensembles from structures with engineered PEV or homologous deletions. Complete PC spectra recorded the bending (Figure 9A) and rotary (Figure 9B) flexibility of the two hinges. The hinge distributions formed two distinct relations. The *T. maritima/A. aeolicus* native and deletion FliG$_{MC}$ structure ensembles formed one relation (R^2 = 0.99) that spanned a large GG hinge range due to the presence of both unstacked and stacked structures. The structures with the stacking interaction had markedly reduced GG hinge flexibility, partly compensated by increased flexibility at the MFVF hinge. The *H. pylori* relation (R^2 = 0.97) had a similar range for MFVF hinge-bending flexibility, but its reduction was coupled to decrease, not increase, at the GG hinge. The deletions reduced GG hinge-bending flexibility, as expected from the reduced helix$_{MC}$ length, hence leverage.

Comparison of the two most divergent *T. maritima* and *H. pylori* X-ray crystal structures reveals that in both cases, ARM-C moves between coaxial and orthogonal orientations with respect to FliG$_M$ (Figure S6). The coaxial ARM-C structures, *H. pylori* PDB: 3USW and *T. maritima* PDB: 3AJC, were most similar to PDB: 4FHR. TH displacements produced by rotational flexibility of the MFVF hinge were determined from the main PC modes. MFVF hinge rotation was the principal (PC1) motion for both structures (Movies S7 and S8). While the PDB: 3USW angle distribution has similar form to the PDB: 4FHR PC3 distribution, the PDB: 3AJC distribution is asymmetric. In both cases, the motions were restricted compared with the corresponding PDB: 4FHR rotation (Figure 9C). Therefore, the large PDB: 4FHR MFVF hinge rotation is not intrinsic to FliG$_{MC}$, but a consequence of complex formation.

Local helix$_{MC}$/GG hinge dynamics gave insight into the regulation of domain motions by this hinge and deletions within it (Figure S7). The dynamics are different for the unstacked versus stacked *T. maritima* conformations. The C-terminal half of this segmented hinge behaves as an unstructured loop element, rather than an α helix in the stacked (PDB: 3AJC, 4FHR) conformations, despite graft-in of seven missing residues from the unstacked (PDB: 1LKV) structure where these residues form an extended α helix. In contrast, helix$_{MC}$ is more flexible in both *H. pylori* conformations due to a long C-terminal loop segment. The long loop eliminates the compensatory coupling between the two hinges seen for *T. maritima*. In contrast to *T. maritima*, the homologous PQV

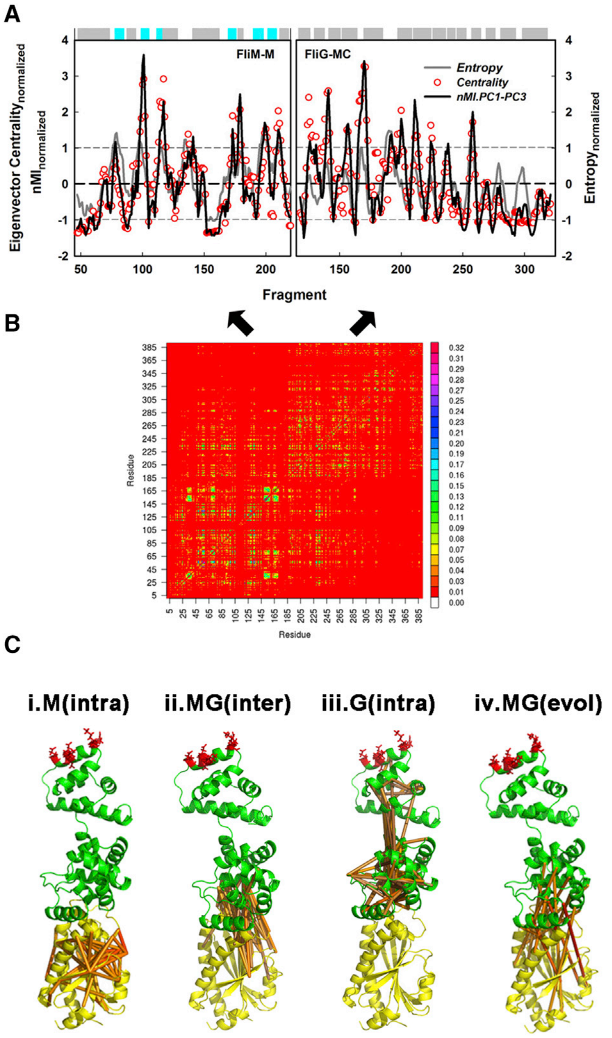

Figure 6. Network Analysis of Local Correlations

(A) The eigenvector centrality tracks the averaged nMI_{PC} profile for the PC1-PC3 motions ($FliM_M$ P_{corr} = 0.96; $FliG_{MC}$ P_{corr} = 0.90). Fragment centrality and entropy are correlated ($FliM_M$ P_{corr} = 0.44; $FliG_{MC}$ P_{corr} = 0.13). Horizontal bar shows secondary structure profile as in Figure 2B.

(B) The covariance matrix. Side bar shows nMI_{local} color scale.

(C) The top (nMI > 0.15; red [high] – bluish brown [low]) (i) $FliM_M$ intra-domain, (ii) $FliM_MFliG_M$ inter-subunit, and (iii) $FliG_{MC}$ intra-domain correlations; and (iv) top coevolved inter-subunit couplings mapped onto the PDB: 4FHR structure, color-coded as in Figure 1.

See also Figures S3 and S4.

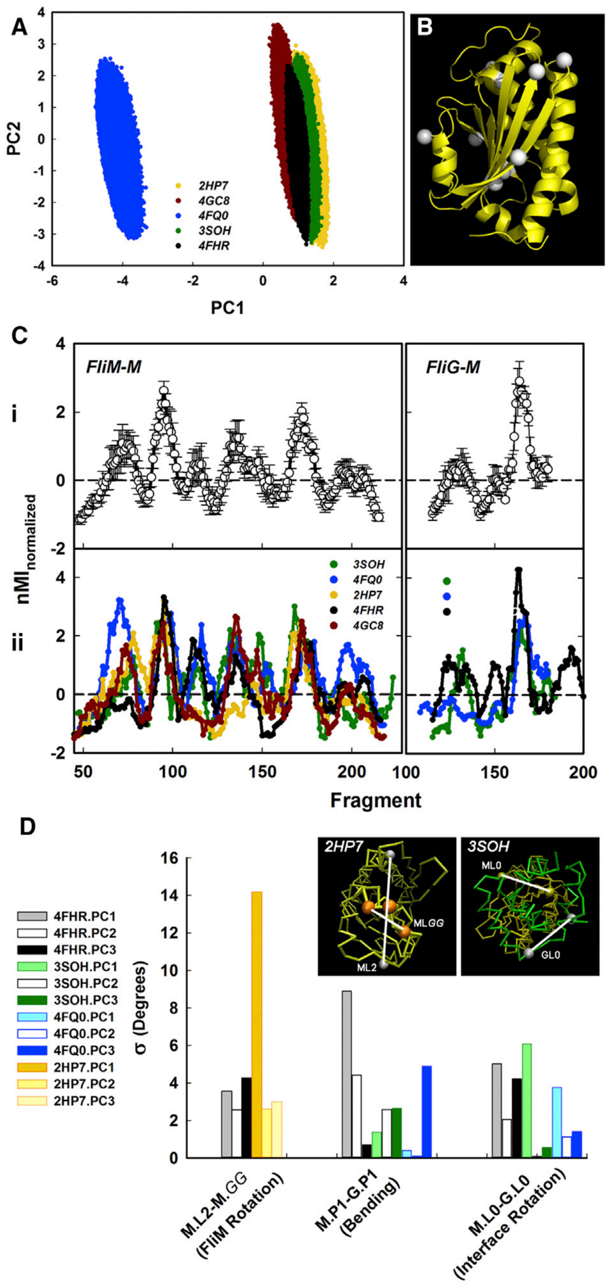

Figure 7. Comparative Dynamics of FliM Structures

(A) PC1PC2 plots of $FliM_M$ ensembles from the monomers (PDB: 2HP7, 4GC8) and complexes (PDB: 3SOH, 4FQ0, 4FHR). The PC1-PC3 conformer spread (σ) was 0.628 ± 0.002 (PDB: 2HP7) and 0.578 ± 0.002 (PDB: 4GC8); 0.448 ± 0.002 (PDB: 3SOH), 0.578 ± 0.002 (PDB: 4FQ0), and 0.582 ± 0.002 (PDB: 4FHR).

(B) Dynamic network nodes (white spheres) mapped onto the PDB: 4FHR $FliM_M$ backbone.

(C) Hinge detection from the fragment nMI_{PC}, contribution. (i) Averaged nMI_{PC1} profile (±σ). (ii) Individual nMI_{PC1} profiles. P_{corr} values (PDB: 4FHR reference) were 0.43 (PDB: 2HP7), 0.52 (PDB: 4GC8), 0.44 (PDB: 3SOH), and 0.57 (PDB: 4FQ0).

(D) First three PC distribution σ of the bending and rotary motions of PDB: 4FHR and $FliM_MFliG_M$ complexes measured with vector pairs as defined in Figure 3D. Insets: snapshots from Movie S4 (PDB: 2HP7) and Movie S5 (PDB: 3SOH) documenting PC1 and complete PC motions.

See also Figure S5.

deletion will more severely reduce the shorter helix in the *H. pylori* serial N-terminal helix/C-terminal loop relay and, hence, torque transfer to the MFVF hinge in the coaxial conformation. In the orthogonal conformation the long C-terminal loop will determine hinge flexibility.

DISCUSSION

The analysis of the *T. maritima* $FliG_MFli_{MC}$ complex revealed the following. (1) Large deviations in FliGc C1-6 residue positions were masked by inter-molecular crystal contacts.

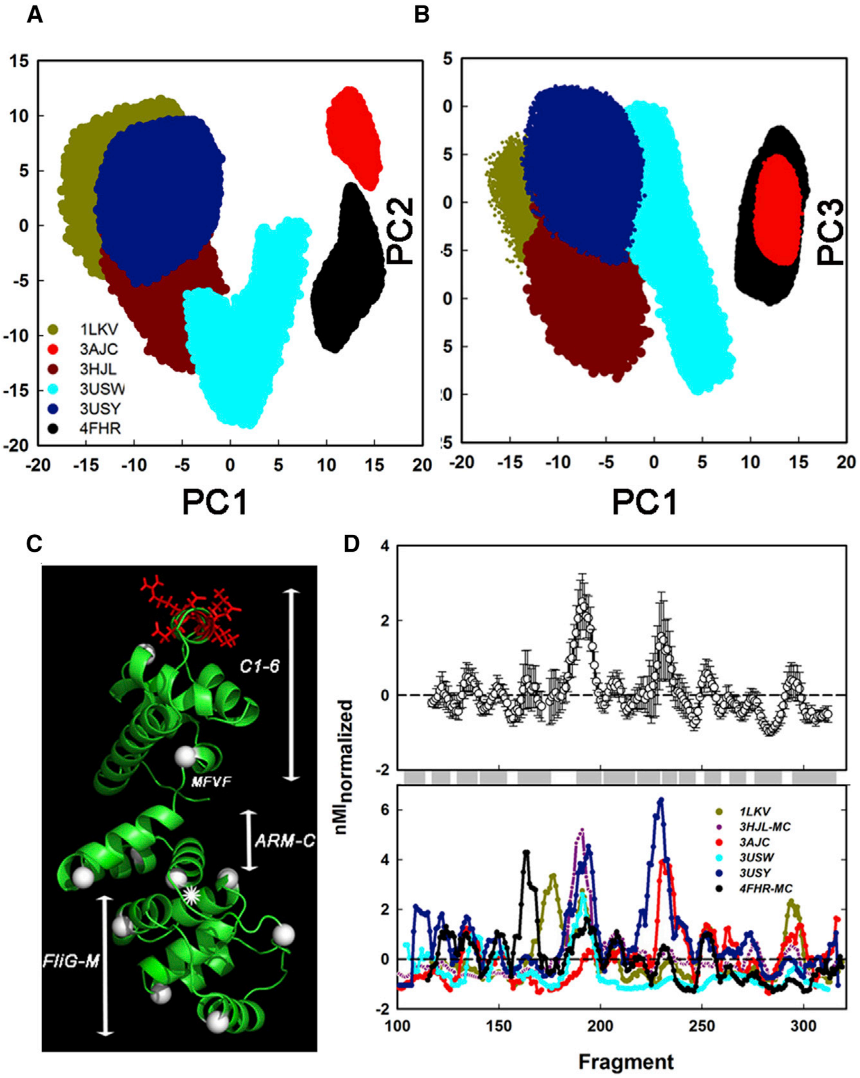

Figure 8. Comparative Dynamics of $FliG_{MC}$ Structures

(A) PC1PC2 plots.

(B) PC1PC3 plots.

(C) Dynamic network nodes (white spheres) mapped onto the PDB: 4FHR $FliG_{Mc}$ backbone. GG pair (asterisk), TH (red side chains).

(D) Averaged nMI_{PC1} profile ($\pm\sigma$) and the individual profiles.

See also Figure S5.

(2) Large C1-6 rotary and bending motions were the output of a two-stage amplification of $FliM_M$ rotary twist fluctuations mediated by the $FliG_{MC}$ GG and MFVF loops. (3) Interfacial loops coupled dynamics of the contacting domains while their internal loops preserved protein fold. (4) $FliG_M$ and ARM-C loops formed a sparsely distributed network. (5) A CW-locked *Salmonella* deletion mimic weakens adjacent $FliG_M$ couplings, but long-range couplings between $FliM_M$ and the TH persist.

The analysis of the FliM and FliG structure library established that: (1) immobilization of the $FliM_M$ GGPG loop upon complex formation generates coaxial rotation of $FliG_M$ relative to $FliM_M$ in *H. pylori* as well as *T. maritima*; (2) different $FliG_{MC}$ conformations from these species show distinct relations between central hinge motions; (3) $FliG_{MC}$ dynamic network architecture is minimally altered by CW-locked deletion mimics; and (4) the $FliG_M$ Fli_{MC} MFXF hinge C1-6 rotation is not matched in isolated $FliG_{MC}$ complexes, despite high intrinsic flexibility. These results integrated with previous knowledge lead to a model for flagellar switch mechanics.

A Mechanical Model for the Flagellar Motor Switch

The model (Figure 10) encapsulates the following mechanical properties.

The $FliM_M$ Switch Module

$FliM_M$ is mechanically stiff, consistent with its role as a dedicated switch module able to propagate conformational transitions distally across FliG to reverse rotor-stator contacts. Complex formation effects a localized change, immobilization of a long loop tethered at both ends to α helices that pivot around the β-sheet center of the $\alpha\beta\alpha$ sandwich to effect $FliG_M$ rotation. The mechanics support the role of $FliM_M$ inter-subunit contacts in transverse conformational spread, as localized by in situ crosslinks and indicated by CW mutations (Park et al., 2006), residue coevolution (Pandini et al., 2015a), and electron paramagnetic spectroscopy (Sircar et al., 2015). Atomic force microscopy data have documented the mechanical rigidity of folds with mixed $\alpha\beta$ topology (Guzman et al., 2010), whereas unshielded β sheets alone deform readily to accommodate shear compared with more rigid, hydrogen-bonded α-helix backbones (Ackbarow et al., 2007). The $FliM_M$ mechanics are in accord with this knowledge.

The $FliG_M$/ARM-C Mechanical Relay

The $FliM_MFliG_M$ interface couples domain motions via a two-point contact between the $FliM_M$ GPGG long loop and $FliG_M$ EHPQ and N-terminal $helix_{MC}$ loops. These couplings link the three layers of the $FliM_M$ sandwich to ARM-M core helices. The ARM-M fold, composed of rigid α-helical levers linked by short loops, forms an elastic domain resilient to deformation during rotation. Its architecture is consistent with the mechanical properties of ARM proteins (Alfarano et al., 2012). $Helix_{MC}$ leverages ARM-M rotation to ARM-C. Engineered N-terminal PEV and homologous deletions in N-terminal $helix_{MC}$ have predictable effects consistent with a shortened lever arm. The MFVF motif, the second central hinge, amplifies ARM-C rotation to C1-6. Torque from $FliM_M$ twist fluctuations is distributed between the hinges, with constrained GG hinge motions compensated for by increased MFVF hinge motions. The flexibility of the composite $helix_{MC}$/GG hinge may be a key source of species variation.

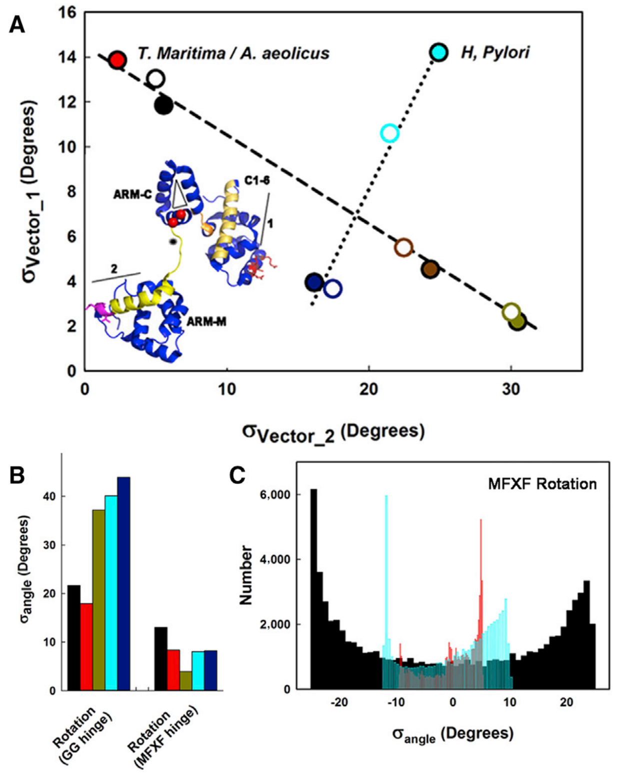

Figure 9. Central Hinge Dynamics

(A) Complete PC hinge-bending amplitudes, recorded as difference angle distributions σ in $FliG_M$ and C1-6 helices relative to ARM-C (inset). Open symbols denote engineered deletion structures (edge color = native structure). Inset: example PDB: 3USY C1-6 terminal helix (1, pale brown), $FliG_M$ $helix_{MC}$ (2, yellow), and ARM-C plane (triangle) (GG pair [red], PQV [magenta], and TH [red side chains]).

(B) Complete PC hinge rotation amplitudes. GL1.GP2 (GG) and GP2.GL2 (MFXF) vector sets (defined in Figure 3C).

(C) PC1 (PDB: 3AJC, 3USY) and PC3 (PDB: 4FHR) MFXF hinge rotation. Colors denote structure ensembles as in Figure 8A.

See also Figures S6 and S7.

Species differences in hinge length and sequence with consequent variations in ARM-C position and domain interactions offer a rationale for the weak ARM-C coevolution signal (Pandini et al., 2015a).

The C1-6 Motor Module

In contrast to $FliG_M$ the C1-6 module is largely devoid of hinge elements, as contacts with adjacent helices attach the TH onto the C3-6 fold, consistent with coevolution data (Pandini et al., 2015a). Short loops adjacent to the TH fine-tune its orientation relative to C1-6 collective motions. The in situ crosslinks target the loops adjacent to the TH as well as the $FliG_M$ interfacial loops (Figure 10). Steric constraints at these end locations would be maximally effective in blocking $FliG_{MC}$ bending motions.

Implications for Mechanism

Structural models of the flagellar motor switch, reviewed in Stock et al. (2012), seek to explain the large TH reorientation in terms of altered domain contacts. The models agree that $FliM_M$ contacts with $FliG_{MC}$ are critical, but differ on the nature of the contacts. One set of models, based on crystallographic data, takes alterations in the $FliM_M$-$FliG_M$ contact as pivotal and sufficient to explain switching. Other models, based on biochemical evidence and presumed mismatch between FliM and FliG subunit stoichiometry in the C ring, posit the pivotal contact as being between $FliM_M$ and FliG ARM-C, although some $FliM_M$ units also contact $FliG_M$. Our study strengthens the case for a pivotal $FliM_M$-$FliG_M$ contact.

The PDB: 4FHR complex reveals that complex formation accentuates a large, angular TH reorientation. The reorientation is still 2-fold, or more, lower than is documented in situ. Additional factors will operate in the C ring. First, hinge-bending motions dominant in the isolated complexes are likely to be blocked by adjacent C-ring subunits and might be compensated for by increased rotation. Second, our study does not address whether intra- or inter-molecular $FliG_{MC}$ stacking interactions exist in the C ring. An extended $helix_{MC}$ in the alternative inter-molecular stacking interaction, as recently proposed (Baker et al., 2016; Sircar et al., 2015), would provide greater leverage for rotation of $FliG_C$. Intra- and inter-molecular stacking contacts observed in the crystals are similar. A solution study of the salt dependence shows that the conformations are interchangeable (Baker et al., 2016).

The stacked *T. martima* conformation in the PEV deletion structure may represent a CW-locked state (Minamino et al., 2011). However, $helix_{MC}$ is soft due to an unstructured C-terminal segment and N-terminal PEV, and homologous deletions do not switch unstacked to stacked $FliG_{MC}$ configurations. Instead, the stacking interaction is strong enough to deform $helix_{MC}$. The two *H. pylori* $FliG_{MC}$ conformations provide snapshots compatible with the in vivo data (Lam et al., 2012), yet their conformational ensembles overlap with themselves and with other unstacked $FliG_{MC}$ conformers (Figure 8). Therefore, we suggest that the stacking interaction provides a mechanism for conformational selection of an intrinsically flexible protein. Weak stacking interactions summed over the ring will provide the free energy difference to lock in the two rotation states. A functional design for the flagellar motor switch requires flexible downstream elements to rapidly switch conformation with minimal energy dissipation, once switching is initiated. Subunits chemically bonded in distinct conformations would dissipate energy and switch slowly. FliG assembles tightly onto FliF (Levenson et al., 2012) and templates the assembly of $FliM(FliN)^3$ distal C-ring complexes (McDowell et al., 2015). Electron microscopy data indicate that the latter may stabilize the FliG ring since it is not clearly visualized in FliFFliG complexes due to presumed disorder (Suzuki et al., 2004), in contrast to the intact C ring (Thomas et al., 2006). Inter-molecular stacking provides a straightforward explanation for how FliG subunits carrying the PEV deletion would favor decreased circumference with a shortened $helix_{MC}$, leading to smaller or more densely packed CW C rings consistent with adaptive remodeling (Lele and Berg, 2015).

The Broader Context: Relevance and Prospects

This study illustrates the importance of backbone flexibility analysis for interpretation of mutagenesis data. It extends earlier work on the F_0 ATP synthase (Pandini et al., 2015b) to show that long-range elastic couplings across subunit interfaces contribute to the coevolution signal. Elastic backbone effects have also been

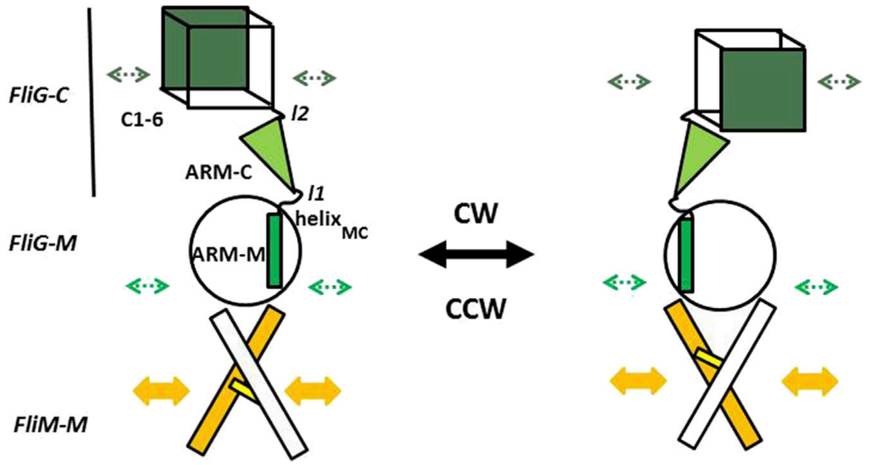

Figure 10. Mechanical Model of the Flagellar Motor Switch

Rotary twist of the stiff $FliM_M$ domain is transmitted via a localized two-point contact to FliG ARM-M. Two central hinges (l1 = GG motif loop, l2 = MFXF motif loop) bounding ARM-C partition $FliG_{MC}$ into three segments, providing two-stage amplification for $FliG_C$ C1-6 reorientation. Long loop l1 flexibility depends on ARM-M/ARM-C stacking interactions. Arrowheads denote location of in situ FliG cross-links (green) and conformational coupling between adjacent $FliM_M$ (yellow).

noted in coevolution analysis of protein-folding landscapes (Morcos et al., 2014; Sutto et al., 2015). These studies add to the literature, cited in the Introduction, stating that coevolved mutations reflect protein conformational dynamics.

The challenge now is to understand the design principles for evolution of protein-protein interactions. Functional modes should provide a more fine-tuned analysis of the dynamics (Hub and de Groot, 2009). Comparative analysis between natural and designed sequences has shown that optimal backbone flexibility is needed for a strong coevolution signal (Ollikainen and Kortemme, 2013). Optimization constraints may explain why some dynamic couplings have coevolved in the $FliM_M FliG_{MC}$ signal complex and others have not. X-Ray structure libraries of rotary motor assemblies, with mechanics that can be measured by single-molecule techniques, are an important stimulus for the development of such analytical tools to study the relation between protein evolution and dynamics.

EXPERIMENTAL PROCEDURES

Generation of tCONCOORD Conformational Ensembles

The X-ray structure library used in this study was downloaded from the PDB. Secondary structure elements and the contact interface within the $FliM_M FliG_{MC}$ complex (PDB: 4FHR) are mapped in Figure S1. Component structures (monomers and partial complexes) are described in Figure S2. tCONCOORD produced a conformational ensemble from each X-ray structure. First, atomic pair distances with upper and lower limits were generated from the structure based on tables of bonding interactions (covalent bonds, hydrogen bonds, salt bridges, etc.) constructed from statistical analysis of the PDB database (de Groot et al., 1997). Second, a new structure was built starting from atoms positioned randomly within a bounding volume around their X-ray coordinates. Successive iterations were performed until convergence was achieved upon satisfaction of the distance constraints or an iteration limit (500) was reached. The structure was rebuilt many times to generate $(256)^2$ = 65,536 equilibrium conformations with full atom detail. tCONCOORD samples large conformational protein transitions by breakage of labile hydrogen bonds solvated by surrounding residues, as validated by test cases and MD simulations (Seeliger et al., 2007).

Principal Component Analysis

PCA, specifically of MD trajectories, was introduced when Amadei et al. (1993) showed that the configurational space can be partitioned into an "essential" subspace with few degrees of freedom describing large-scale slow anharmonic motions, with the remaining space describing local fluctuations. Functional motions of biologically relevant conformational transitions belong to the essential subspace defined by the first few PCs. These physically represent the largest-amplitude collective motions in the macromolecular assembly (de Groot et al., 1996). The variance (σ^2) was taken as a measure of "motion" (Pandini et al., 2015b). The combined variance of the statistically independent, first three PCs (or subset) from the average structure was obtained by summation. Geometric angular distributions between selected vector pairs were used to compute the torsional stiffness and bending moments.

Network Analysis

The conformational dynamics of four-residue fragments were encoded with the SA (Figure S2) for elucidation of the mechanical relays underlying collective PC motions and comparison with the coevolution network. Frequently occurring conformations from 798 high-resolution X-ray structures were extracted as representative fragment states (letters) (Pandini et al., 2010). The SA provides an enriched string set of local conformational states for accurate reconstruction of protein fold. Statistically significant correlations were determined and analyzed with GSATools (Pandini et al., 2013).

Coevolution Analysis

Pfam protein sequence families $FliM_M$ (PF02154), $FliG_M$ (PF14821), and $FliG_C$ (PF01706) (Finn et al., 2010) were filtered at 80% redundancy level. PSICOV-based analysis of residue coevolution between FliM and FliG (Pandini et al., 2015a) was supplemented with direct coupling analysis (Morcos et al., 2011) to increase contact prediction accuracy (Jones et al., 2014). Sequences were matched based on organism membership and genomic locus proximity (<100 genes, *fliG-fliM* distance = 18 ± 24). The final dataset contained more than 1,400 non-redundant, concatenated sequences. The coevolution network was constructed from the top 1.5% correlations, a cut-off intermediate between 2σ (2.2%) and 3σ (0.3%). Randomized libraries generated by shuffling within MSA residue positions assessed significance (Pandini et al., 2015a).

See Supplemental Experimental Procedures for operational details and formalism.

SUPPLEMENTAL INFORMATION

Supplemental Information includes Supplemental Experimental Procedures, seven figures, eight movies, and four molecular models and can be found with this article online at http://dx.doi.org/10.1016/j.str.2016.05.012.

AUTHOR CONTRIBUTIONS

Conceptualization: A.P., F.M., and S.K.; Methodology: A.P., F.M., and S.K.; Software: A.P. and F.M.; Validation: S.K.; Writing – First Draft: A.P. and S.K.; Writing – Review & Editing: A.P., F.M., and S.K.; Visualization: F.M. and S.K.; Supervision/Project Administration: S.K.; Funding Acquisition: F.M. and S.K.

ACKNOWLEDGMENTS

This work was supported by the Royal Society Collaborative Exchange Grant UI175.70592, the Molecular Biology Consortium (S.K) and the Francis Crick

Institute which receives core funding from Cancer Research UK, the UK Medical Research Council, and Wellcome Trust through the Taylor (10179) group.

Received: February 22, 2016
Revised: April 26, 2016
Accepted: May 23, 2016
Published: June 23, 2016

REFERENCES

Ackbarow, T., Chen, X., Keten, S., and Buehler, M.J. (2007). Hierarchies, multiple energy barriers, and robustness govern the fracture mechanics of alpha-helical and beta-sheet protein domains. Proc. Natl. Acad. Sci. USA *104*, 16410–16415.

Alfarano, P., Varadamsetty, G., Ewald, C., Parmeggiani, F., Pellarin, R., Zerbe, O., Pluckthun, A., and Caflisch, A. (2012). Optimization of designed armadillo repeat proteins by molecular dynamics simulations and NMR spectroscopy. Protein Sci. *21*, 1298–1314.

Amadei, A., Linssen, A.B., and Berendsen, H.J. (1993). Essential dynamics of proteins. Proteins *17*, 412–425.

Bai, F., Che, Y.S., Kami-ike, N., Ma, Q., Minamino, T., Sowa, Y., and Namba, K. (2013). Populational heterogeneity vs. temporal fluctuation in Escherichia coli flagellar motor switching. Biophys. J. *105*, 2123–2129.

Baker, M.A., Hynson, R.M., Ganuelas, L.A., Mohammadi, N.S., Liew, C.W., Rey, A.A., Duff, A.P., Whitten, A.E., Jeffries, C.M., Delalez, N.J., et al. (2016). Domain-swap polymerization drives the self-assembly of the bacterial flagellar motor. Nat. Struct. Mol. Biol. *23*, 197–203.

Bray, D., and Duke, T. (2004). Conformational spread: the propagation of allosteric states in large multiprotein complexes. Annu. Rev. Biophys. Biomol. Struct. *33*, 53–73.

Brown, P.N., Hill, C.P., and Blair, D.F. (2002). Crystal structure of the middle and C-terminal domains of the flagellar rotor protein FliG. EMBO J. *21*, 3225–3234.

Czub, J., and Grubmuller, H. (2014). Rotation triggers nucleotide-independent conformational transition of the empty beta subunit of F(1)-ATPase. J. Am. Chem. Soc. *136*, 6960–6968.

de Groot, B.L., van Aalten, D.M., Amadei, A., and Berendsen, H.J. (1996). The consistency of large concerted motions in proteins in molecular dynamics simulations. Biophys. J. *71*, 1707–1713.

de Groot, B.L., van Aalten, D.M., Scheek, R.M., Amadei, A., Vriend, G., and Berendsen, H.J. (1997). Prediction of protein conformational freedom from distance constraints. Proteins *29*, 240–251.

Dyer, C.M., Vartanian, A.S., Zhou, H., and Dahlquist, F.W. (2009). A molecular mechanism of bacterial flagellar motor switching. J. Mol. Biol. *388*, 71–84.

Fernandez, A., and Scheraga, H.A. (2003). Insufficiently dehydrated hydrogen bonds as determinants of protein interactions. Proc. Natl. Acad. Sci. USA *100*, 113–118.

Finn, R.D., Mistry, J., Tate, J., Coggill, P., Heger, A., Pollington, J.E., Gavin, O.L., Gunasekaran, P., Ceric, G., Forslund, K., et al. (2010). The Pfam protein families database. Nucleic Acids Res. *38*, D211–D222.

Guzman, D.L., Randall, A., Baldi, P., and Guan, Z. (2010). Computational and single-molecule force studies of a macro domain protein reveal a key molecular determinant for mechanical stability. Proc. Natl. Acad. Sci. USA *107*, 1989–1994.

Highsmith, S., Wang, C.C., Zero, K., Pecora, R., and Jardetzky, O. (1982). Bending motions and internal motions in myosin rod. Biochemistry *21*, 1192–1197.

Hub, J.S., and de Groot, B.L. (2009). Detection of functional modes in protein dynamics. PLoS Comput. Biol. *5*, e1000480.

Jones, D.T., Singh, T., Kosciolek, T., and Tetchner, S. (2014). MetaPSICOV: combining coevolution methods for accurate prediction of contacts and long range hydrogen bonding in proteins. Bioinformatics *31*, 999–1006.

Lam, K.H., Ip, W.S., Lam, Y.W., Chan, S.O., Ling, T.K., and Au, S.W. (2012). Multiple conformations of the FliG C-terminal domain provide insight into flagellar motor switching. Structure *20*, 315–325.

Lee, L.K., Ginsburg, M.A., Crovace, C., Donohoe, M., and Stock, D. (2010). Structure of the torque ring of the flagellar motor and the molecular basis for rotational switching. Nature *466*, 996–1000.

Lele, P.P., and Berg, H.C. (2015). Switching of bacterial flagellar motors is triggered by mutant FliG. Biophys. J. *108*, 1275–1280.

Levenson, R., Zhou, H., and Dahlquist, F.W. (2012). Structural insights into the interaction between the bacterial flagellar motor proteins FliF and FliG. Biochemistry *51*, 5052–5060.

Lloyd, S.A., and Blair, D.F. (1997). Charged residues of the rotor protein FliG essential for torque generation in the flagellar motor of *Escherichia coli*. J. Mol. Biol. *266*, 733–744.

Lux, R., Kar, N., and Khan, S. (2000). Overproduced *Salmonella typhimurium* flagellar motor switch complexes. J. Mol. Biol. *298*, 577–583.

Ma, Q., Nicolau, D.V., Jr., Maini, P.K., Berry, R.M., and Bai, F. (2012). Conformational spread in the flagellar motor switch: a model study. PLoS Comput. Biol. *8*, e1002523.

Magariyama, Y., Yamaguchi, S., and Aizawa, S. (1990). Genetic and behavioral analysis of flagellar switch mutants of *Salmonella typhimurium*. J. Bacteriol. *172*, 4359–4369.

McDowell, M.A., Marcoux, J., McVicker, G., Johnson, S., Fong, Y.H., Stevens, R., Bowman, L.A., Degiacomi, M.T., Yan, J., Wise, A., et al. (2015). Characterisation of Shigella Spa33 and Thermotoga FliM/N reveals a new model for C-ring assembly in T3SS. Mol. Microbiol. *99*, 749–766.

Minamino, T., Imada, K., Kinoshita, M., Nakamura, S., Morimoto, Y.V., and Namba, K. (2011). Structural insight into the rotational switching mechanism of the bacterial flagellar motor. PLoS Biol. *9*, e1000616.

Morcos, F., Jana, B., Hwa, T., and Onuchic, J.N. (2013). Coevolutionary signals across protein lineages help capture multiple protein conformations. Proc. Natl. Acad. Sci. USA *110*, 20533–20538.

Morcos, F., Pagnani, A., Lunt, B., Bertolino, A., Marks, D.S., Sander, C., Zecchina, R., Onuchic, J.N., Hwa, T., and Weigt, M. (2011). Direct-coupling analysis of residue coevolution captures native contacts across many protein families. Proc. Natl. Acad. Sci. USA *108*, E1293–E1301.

Morcos, F., Schafer, N.P., Cheng, R.R., Onuchic, J.N., and Wolynes, P.G. (2014). Coevolutionary information, protein folding landscapes, and the thermodynamics of natural selection. Proc. Natl. Acad. Sci. USA *111*, 12408–12413.

Ollikainen, N., and Kortemme, T. (2013). Computational protein design quantifies structural constraints on amino acid covariation. PLoS Comput. Biol. *9*, e1003313.

Pandini, A., Fornili, A., and Kleinjung, J. (2010). Structural alphabets derived from attractors in conformational space. BMC Bioinformatics *11*, 97.

Pandini, A., Fornili, A., Fraternali, F., and Kleinjung, J. (2013). GSATools: analysis of allosteric communication and functional local motions using a structural alphabet. Bioinformatics *29*, 2053–2055.

Pandini, A., Kleinjung, J., Rasool, S., and Khan, S. (2015a). Coevolved mutations reveal distinct architectures for two core proteins in the bacterial flagellar motor. PLoS One *10*, e0142407.

Pandini, A., Kleinjung, J., Taylor, W.R., Junge, W., and Khan, S. (2015b). The phylogenetic signature underlying ATP synthase c-ring compliance. Biophys. J. *109*, 975–987.

Park, S.Y., Lowder, B., Bilwes, A.M., Blair, D.F., and Crane, B.R. (2006). Structure of FliM provides insight into assembly of the switch complex in the bacterial flagella motor. Proc. Natl. Acad. Sci. USA *103*, 11886–11891.

Paul, K., Brunstetter, D., Titen, S., and Blair, D.F. (2011). A molecular mechanism of direction switching in the flagellar motor of *Escherichia coli*. Proc. Natl. Acad. Sci. USA *108*, 17171–17176.

Sagi, Y., Khan, S., and Eisenbach, M. (2003). Binding of the chemotaxis response regulator CheY to the isolated, intact switch complex of the bacterial flagellar motor: lack of cooperativity. J. Biol. Chem. *278*, 25867–25871.

Sarkar, M.K., Paul, K., and Blair, D. (2010). Chemotaxis signaling protein CheY binds to the rotor protein FliN to control the direction of flagellar rotation in *Escherichia coli*. Proc. Natl. Acad. Sci. USA *107*, 9370–9375.

Seeliger, D., Haas, J., and de Groot, B.L. (2007). Geometry-based sampling of conformational transitions in proteins. Structure *15*, 1482–1492.

Sfriso, P., Duran-Frigola, M., Mosca, R., Emperador, A., Aloy, P., and Orozco, M. (2016). Residues coevolution guides the systematic identification of alternative functional conformations in proteins. Structure *24*, 116–126.

Sircar, R., Borbat, P.P., Lynch, M.J., Bhatnagar, J., Beyersdorf, M.S., Halkides, C.J., Freed, J.H., and Crane, B.R. (2015). Assembly states of FliM and FliG within the flagellar switch complex. J. Mol. Biol. *427*, 867–886.

Sourjik, V., and Berg, H.C. (2002). Binding of the *Escherichia coli* response regulator CheY to its target measured in vivo by fluorescence resonance energy transfer. Proc. Natl. Acad. Sci. USA *99*, 12669–12674.

Stock, D., Namba, K., and Lee, L.K. (2012). Nanorotors and self-assembling macromolecular machines: the torque ring of the bacterial flagellar motor. Curr. Opin. Biotechnol. *23*, 545–554.

Sutto, L., Marsili, S., Valencia, A., and Gervasio, F.L. (2015). From residue coevolution to protein conformational ensembles and functional dynamics. Proc. Natl. Acad. Sci. USA *112*, 13567–13572.

Suzuki, H., Yonekura, K., and Namba, K. (2004). Structure of the rotor of the bacterial flagellar motor revealed by electron cryomicroscopy and single-particle image analysis. J. Mol. Biol. *337*, 105–113.

Thomas, D.R., Francis, N.R., Xu, C., and DeRosier, D.J. (2006). The three-dimensional structure of the flagellar rotor from a clockwise-locked mutant of *Salmonella enterica* serovar *Typhimurium*. J. Bacteriol. *188*, 7039–7048.

Vartanian, A.S., Paz, A., Fortgang, E.A., Abramson, J., and Dahlquist, F.W. (2012). Structure of flagellar motor proteins in complex allows for insights into motor structure and switching. J. Biol. Chem. *287*, 35779–35783.

Yuan, J., and Berg, H.C. (2013). Ultrasensitivity of an adaptive bacterial motor. J. Mol. Biol. *425*, 1760–1764.

Zhou, J., Lloyd, S.A., and Blair, D.F. (1998). Electrostatic interactions between rotor and stator in the bacterial flagellar motor. Proc. Natl. Acad. Sci. USA *95*, 6436–6441.

Structure, Volume *24*

Supplemental Information

The Gearbox of the Bacterial Flagellar Motor Switch

Alessandro Pandini, Faruck Morcos, and Shahid Khan

SUPPLEMENTAL INFORMATION

Supplemental Data

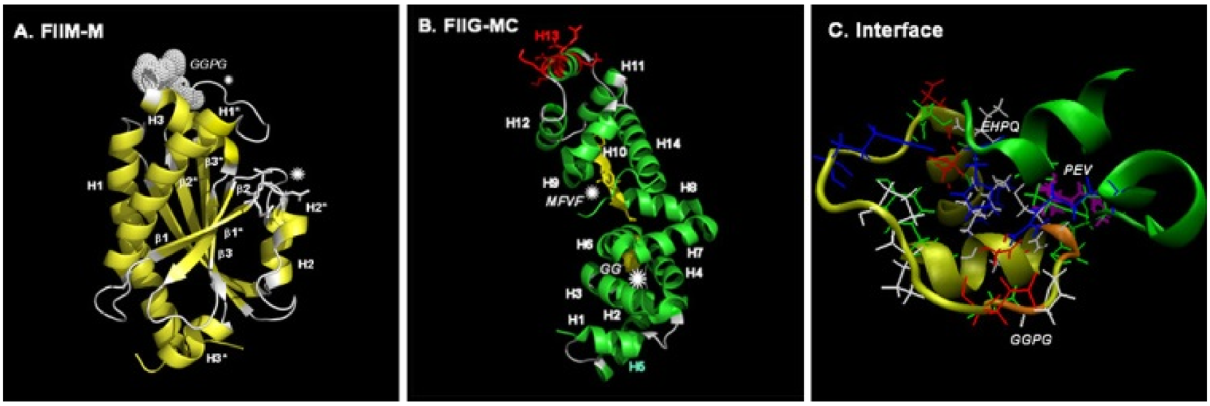

Figure S1, Related to Figure 1: 4FHR Structure Index. *4FHR domain structures and inter-helix loops (white backbone). Asterisks mark segments with high B-factors computed from the conformational ensemble; asterisk size is proportional to B-factor value.* ***A****. FliM$_M$ domain. GGPG motif = white spheres.* ***B****. FliG$_{MC}$ domains. GG / MFVF motifs (yellow segments), TH (H13, red letters / charged residue sidechains) and helix$_{MC}$ (H5, pale green letters) are marked. Helices H1-H5 form the FliG$_M$ ARM domain (ARM-M). FliG$_C$ has two sub-domains either side of the MFXF motif, N-terminal ARM-C (H6-H8), and C-terminal six helix bundle C1-6 (H9-H14).* ***C.*** *Contact residue side-chains at the FliM$_M$ (yellow backbone) and FliG$_M$ (green backbone) interface coloured according to residue type (non-polar (white), acidic (red), basic (blue)). GGPG (orange backbone) and PEV (magenta sidechains) residues are marked.*

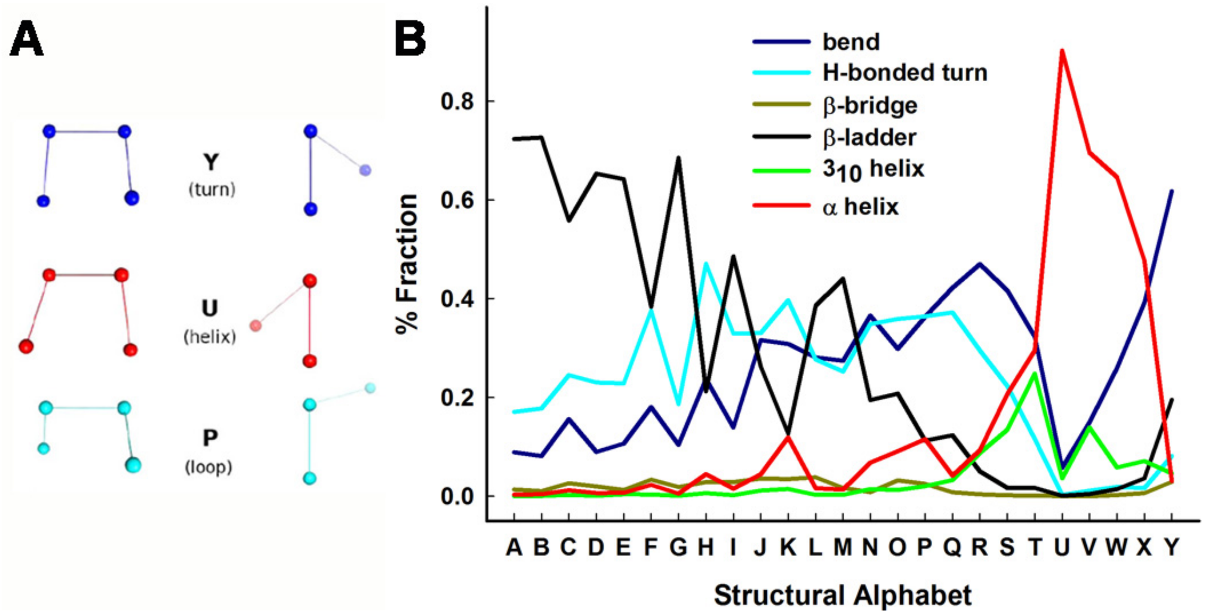

Figure S2, Related to Figure 5A: A. Examples of SA letters. *The SA has 25 letters representing fragments of 4 consecutive C^{α} atoms. Each letter represents a prototypical conformational state. Loop specific conformations are as listed in STRIDE. "The relation between the two views is a 90° rotation around a vertical axis in the paper plane and an adjustment to align the two central atoms to a Newman projection" (from (Pandini et al., 2010) with permission).* ***B. SA secondary structure assignment.*** *The letters were assigned based on screen of 798 high-resolution X-ray structures in PDB. The figure presents a more detailed correspondence of SA with secondary structure elements than initially published (Pandini et al., 2010). Secondary structure nomenclature is in accordance with the DSSP database (Touw et al., 2015).*

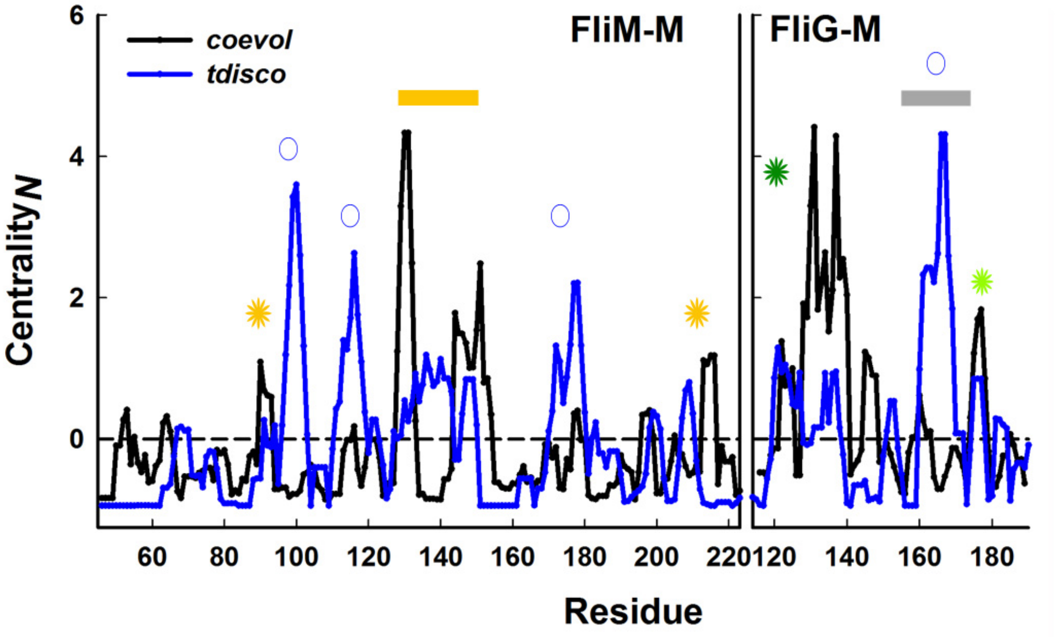

Figure S3, Related to Figure 6C: Coevolution and dynamic network centrality. *Centrality$_N$ = ((Cent$_{residue}$ – Cent$_{mean}$)/σ_{cent}), where Cent$_{mean}$ and σ_{cent} are mean and standard deviation respectively of the residue centrality (Cent$_{residue}$). The GPGG motif loop (yellow bar) and EHPQ motif (green asterisk) are part of the FliMFliG interface. Coevolution signal from these interfacial contacts was reported (Pandini et al., 2015a). FliM$_M$ sheet β1 and β3* (yellow asterisks) are dynamically coupled to FliG$_M$ core helix H2 adjacent to the EHPQ motif C-terminus. These elements are not in contact as seen from the structural map (Figure 6C). The helix$_{MC}$ (grey bar) C-terminal GG loop (light green asterisk) is also important for interface coevolution and dynamics. Other dynamic couplings (open circles), notably helix$_{MC}$ itself, and other strands in the FliM$_M$ β core lack a coevolution signal. P$_{corr}$ = 0.03.*

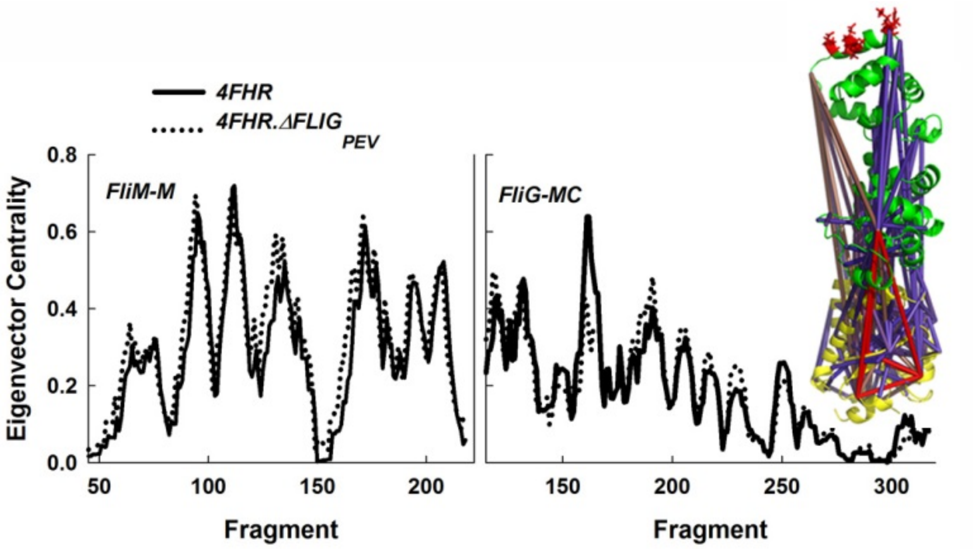

Figure S4, Related to Figure 6A: Perturbation of FliG$_{MC}$ dynamics by ΔPEV. *The energy minimized T. maritima 4FHR PEV deletion protein complex had 798 fewer atom-to-atom contacts in FliG$_M$, of which 431 contacts were formed by the deleted residues. The interfacial contacts between FliM$_M$ and FliG$_M$ were reduced from 530 to 468. T. maritima PEV = PQV (H. pylori), VQY (A. aeolicus). The 4FHR network centrality (± FliG$_{MC}$ PEV) P_{corr} = 0.91.* ***Image:*** *Top FliG$_M$-FliG$_{MC}$ interfacial correlations mapped onto the 4FHR deletion mutant structure.*

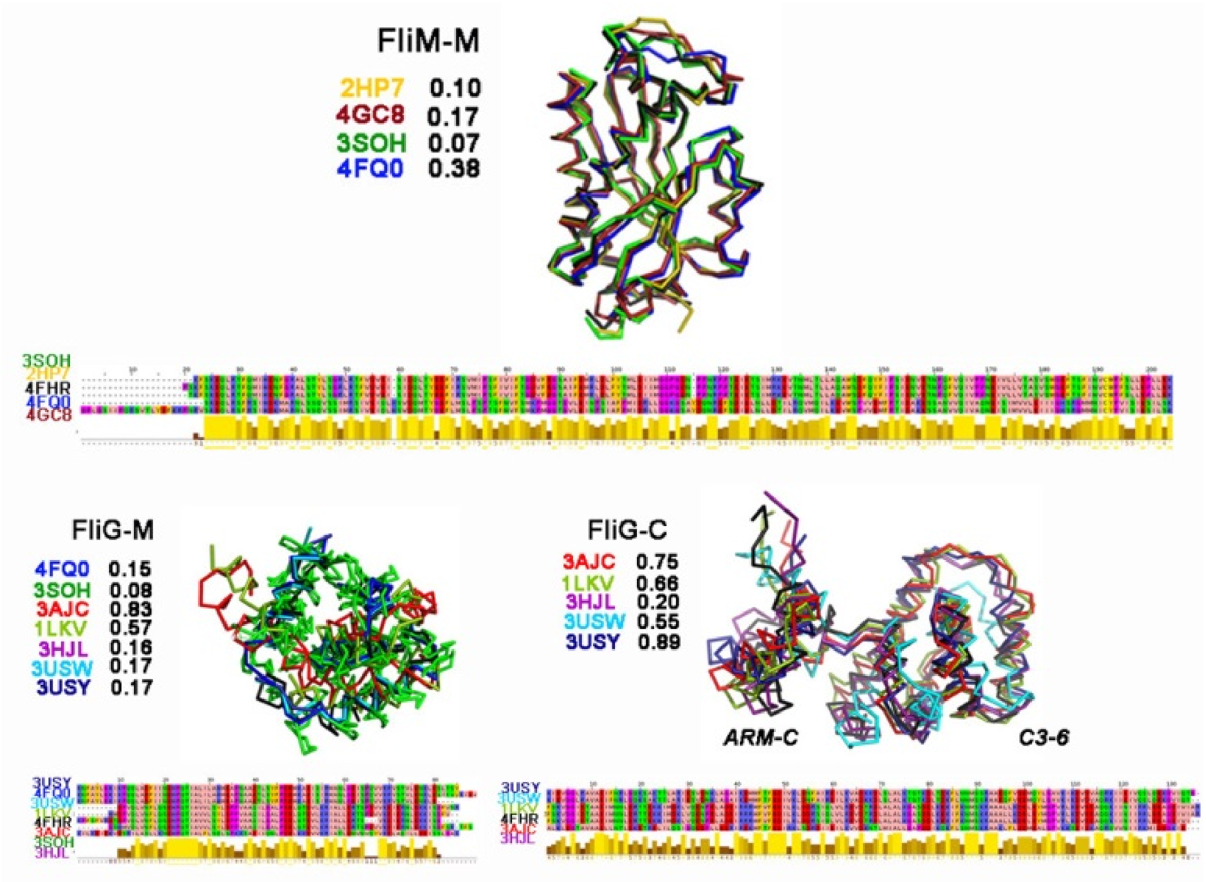

Figure S5, Related to Figures 7 & 8: Conformational variability between flagellar motor protein structures. *$FliM_M$ (T. maritima 2HP7(Park et al.,2006), H. pylori GC8 (Lam et al., 2013)), $FliG_{MC}$,(T. maritima 1LKV (Brown et al.,2002), 3AJC (Minamino et al.,2011), 3USY, 3USW(Lam et al.,2012); $FliG_{NMC}$ (A. aeolicus 3HJL (Lee et al.,2010); $FliM_M FliG_M$ (T. maritima 3SOH (Paul et al., 2011), H. pylori 4FQ0 (Lam et al., 2013)) and $FliM_M FliG_{MC}$ (T. maritima 4FHR). Superimpositions of the X-ray crystal structures for each domain are shown above the MSA of their sequences. The residues in the MSA are coloured according to type (Zappo colouring). T. maritima motility is powered by a single, monopolar flagellum with dominantly CW rotation, interspersed with brief CCW episodes that reorient the bacteria (Gluch et al., 1995). The epsilon proteobacterium, H. pylori, has a monopolar bundle with sheathed flagella to withstand acid pH in the human stomach (Lertsethtakarn et al., 2011). A. aeolicus is an ancient, thermophilic species like T. maritima, with monopolar polytrichous flagella like H. pylori (Takekawa et al., 2015). The RMSDs (angstrom^2) of the structures from the reference 4FHR structure are listed. The $FliM_M$ domain structures (T.maritima RMSD = 0.08 ± 0.02 A^2; H. pylori RMSD = 0.27 ± 0.15 A^2) were arguably different between species; but there was no meaningful difference between the $FliG_M$ domain structures (A. aeolicus RMSD = 0.16 A^2; T. maritima = 0.49 ± 0.38 A^2; H. pylori = 0.16 ± 0.01 A^2). T. maritima $FliG_{MC}$ structures 1LKV and 3AJC had greater RMSD from 4FHR $FliG_M$ compared to the A. aeolicus or H. pylori structures. The $FliG_c$ domain structures were the most variable. The $FliG_c$ RMSDs of other T. maritima structures from 4FHR were comparable to structures from other species (A. aeolicus RMSD = 0.2 A^2; T. maritima = 0.81 ± 0.08 A^2; H. pylori = 0.72 ± 0.24 A^2), as for $FliG_M$,. The 3AJC structure has the PEV deletion, homologous to the PAA deletion in the CW-locked Salmonella mutant.*

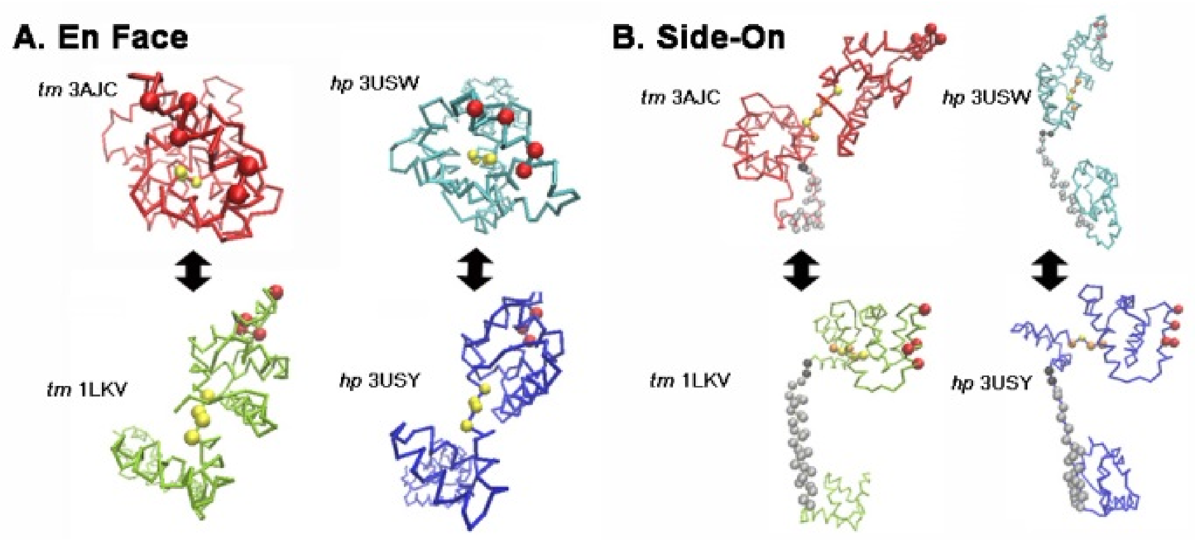

Figure S6, Related to Figure 9A: Comparison of the most divergent T. maritima (tm) and H. pylori (hp) structures. A. *En-face views show co-axial orientation of C1-6 in (3AJC, 3USW), and off-axis orientation (~ 45° tilt angle) of C1-6 relative to FliG$_M$ in (1LKV, 3USY).* ***B.*** *Side-on views show helix$_{MC}$ (grey spheres) split into two segments in 3AJC, but linearly extended in the other structures. The N-terminal half of helix$_{MC}$ contacts ARM-M in the hp structures. GG pair (black spheres); MFXF motif (yellow spheres); TH charged residues (red spheres).*

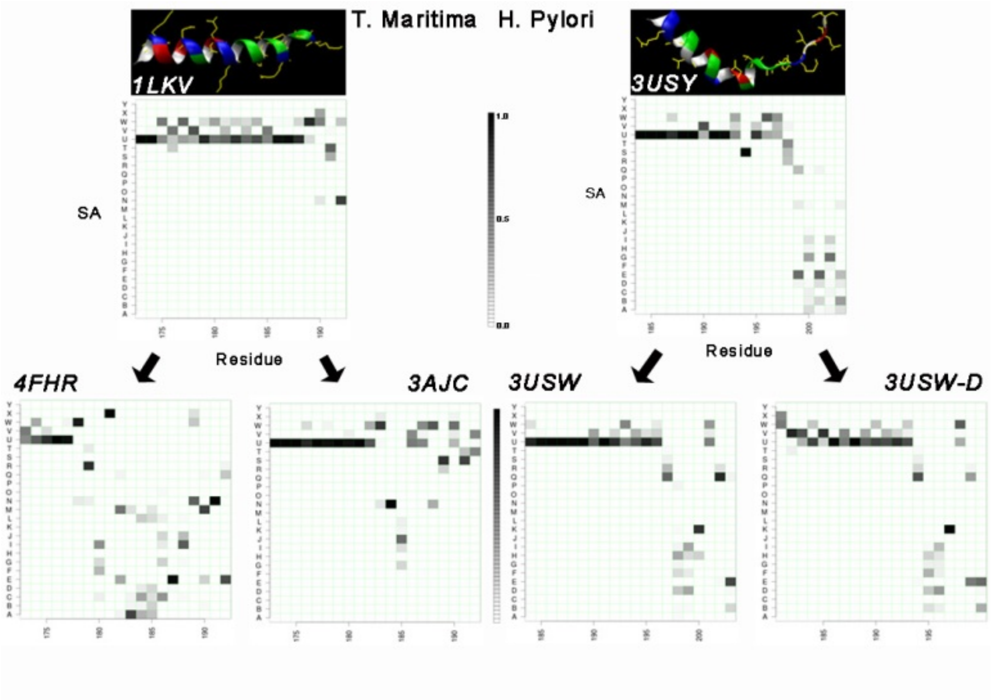

Figure S7, Related to Figure 9A: Helix$_{MC}$ dynamics. *Helix$_{MC}$ - GG loop (25 residue segment) conformational spectra. The segment includes 7 residues grafted from 1LKV / 3USW conformations to others where these were not resolved. Greyscale bars show frequency of the SA-encoded conformations as in Figure 5. Arrows link graft donor–acceptor structures. In isolation, the extended helix$_{MC}$ remains α-helical in T. maritima 1LKV. In contrast, the C-terminal third of H. pylori 3USY adopts β-sheet conformations. In the 3AJC / 4FHR structures with the intramolecular ARM-M H11 / ARM-C H13-H14 stacking contact (Vartanian et al.,2012) the helix, including the grafted residues, is disrupted. In 3AJC, the central residues adopt loop conformations. In 4FHR, most (C-terminal two-third) of helix$_{MC}$ is non-helical; possibly due to torsion created by FliM$_{M.}$ In the H. pylori 3USW or its deletion (3USW-D), helix$_{MC}$ dynamics are similar to 3USY.* ***Images*** *show the segment in the donor structures. Backbone coloured according to residue type (acid (red); basic (blue); polar (green); hydrophobic (white)) indicates propensity for internal solvation. Crystal contacts (yellow sidechains).*

Supplemental Experimental Procedures

Structure preparation and tCONCOORD simulation

The PDB structure files were prepared for tCONCOORD simulations at neutral pH and 300°K in Molecular Operating Environment 2013.08 (Chemical Computing Group Inc., Montreal, QC H3A 2R7, Canada). Missing residues were grafted in from alternate conformations. The system was energy-minimized with the OPLS-AA force field before simulation within the GROMACS 4.5.5 environment (Pronk et al., 2013), as in the recent extensive comparison of tCONCOORD and MD (Fornili et al., 2013) . Default solvation score was 2.2. Comparison with experimental B-factors and geometrical analyses of the conformer ensembles were performed with the GROMACS *g-rmsd* and *g-sgangle* functions respectively. Crystal contacts were extracted from the PDB files with the CCP4 suite *ncont* utility (Winn et al., 2011).

PCA and network analysis

The PCs were generated by diagonalization of the covariance matrix of C^{α} positions after removal of the overall rotational and translational motions. The combined variance of the PCs was obtained by summation. For example,

$$(\sigma_{PC1\text{-}3})^2 = (\sigma_{PC1})^2 + (\sigma_{PC2})^2 + (\sigma_{PC3})^2 \qquad (1)$$

The SA-encoded tCONCOORD ensembles formed string sets. The variance at fragment positions was given by the Shannon entropy, S_f.

$$S_f = -\sum_{j=1}^{k} p_{ij}.log_2\, p_{ij}; \qquad (2)$$

where p_{ij} is the fraction of the ensemble with fragment position *i* occupied by SA letter *j*.

The SA-encoded covariance matrix was used to generate a network model; with the residues as nodes and the correlations as edges. The contribution of a node to the network scaled with its connectivity, estimated by the eigenvector centrality, *E*, calculated directly from the correlation matrix:

$$E.(M)_{corr} = E.\lambda; \qquad (3)$$

where $(M)_{corr}$ is the correlation matrix and λ the corresponding eigenvalue.

The correlation of conformational changes in a pair of protein segments *(i,j)* was calculated as normalized mutual information (nMI_{local}) between the associated columns in the structural string alignment.

$$nMI_{local}(Ci;\, Cj) = (I(Ci;\, Cj) - \varepsilon(Ci;\, Cj))/H(Ci,\, Cj) \qquad (4)$$

where *Ci* and *Cj* are the relevant columns in the structural string, *I(Ci;Cj)* is the mutual information between them, *H(Ci,Cj)* is the joint entropy, and *ε(Ci;Cj)* is the expected finite size error. Significance (nMI > 0) was determined with the false discovery rate test by comparison against a randomized background distribution obtained by shuffling. The top correlations (nMI > 0.15, <10% of the total) were mapped onto the PDB structures.

The correlation between local and global motions was calculated as the nMI (nMI_{PC}) between the array of fragment states and the array of PC states obtained from the C^{α} covariance matrix.

$$nMI_{PC} = I(Ci;\, sPCj)\, /\, H(Ci,\, sPCj) \qquad (5)$$

where *Ci* is the vector of states sampled by fragment *i*, *sPCj* is the vector of global states associated with the *j*th PC , *I*(*Ci*;*sPCj*) is their MI, and *H*(*Ci*,*sPCj*) is their joint entropy. Further details are given in (Pandini et al.,2012. Pandini et al., 2015b).

Mechanics

The torsional stiffness, K, is given by the equation $\sigma^2 = k_B T K^{-1}$; where k_B = Boltzmann constant and T = temperature. The bending moment, M is computed from the Euler-Bernoulli equation $(\frac{d^2w}{dx^2}) = -M/EI$; where E = Young's modulus, I = area moment of inertia, w = deflection, x = length. Force and M balance, applicable to each segment, apply over the beam.

Supplemental References

Fornili, A., Pandini, A., Lu, H.C., and Fraternali, F. (2013). Specialized Dynamical Properties of Promiscuous Residues Revealed by Simulated Conformational Ensembles. Journal of chemical theory and computation *9*, 5127-5147.

Gluch, M.F., Typke, D., and Baumeister, W. (1995). Motility and thermotactic responses of Thermotoga maritima. Journal of bacteriology *177*, 5473-5479.

Lam, K.H., Lam, W.W., Wong, J.Y., Chan, L.C., Kotaka, M., Ling, T.K., Jin, D.Y., Ottemann, K.M., and Au, S.W. (2013). Structural basis of FliG-FliM interaction in Helicobacter pylori. Molecular microbiology *88*, 798-812.

Lertsethtakarn, P., Ottemann, K.M., and Hendrixson, D.R. (2011). Motility and chemotaxis in Campylobacter and Helicobacter. Annual review of microbiology *65*, 389-410.

Paul, K., Gonzalez-Bonet, G., Bilwes, A.M., Crane, B.R., and Blair, D. (2011). Architecture of the flagellar rotor. The EMBO journal *30*, 2962-2971.

Pronk, S., Pall, S., Schulz, R., Larsson, P., Bjelkmar, P., Apostolov, R., Shirts, M.R., Smith, J.C., Kasson, P.M., van der Spoel, D., *et al.* (2013). GROMACS 4.5: a high-throughput and highly parallel open source molecular simulation toolkit. Bioinformatics (Oxford, England) *29*, 845-854.

Takekawa, N., Nishiyama, M., Kaneseki, T., Kanai, T., Atomi, H., Kojima, S., and Homma, M. (2015). Sodium-driven energy conversion for flagellar rotation of the earliest divergent hyperthermophilic bacterium. Scientific reports *5*, 12711.

Touw, W.G., Baakman, C., Black, J., te Beek, T.A., Krieger, E., Joosten, R.P., and Vriend, G. (2015). A series of PDB-related databanks for everyday needs. Nucleic acids research *43*, D364-368.

Winn, M.D., Ballard, C.C., Cowtan, K.D., Dodson, E.J., Emsley, P., Evans, P.R., Keegan, R.M., Krissinel, E.B., Leslie, A.G., McCoy, A., *et al.* (2011). Overview of the CCP4 suite and current developments. Acta crystallographica *67*, 235-242.

Molecular Models

MM S1, Related to Figure 6: *4FHR FliM$_M$ dynamic correlation network*
MM S2, Related to Figure 6: *4FHR FliM$_M$ – FliG$_M$ inter-subunit dynamic correlation network*
MM S3, Related to Figure 6: *4FHR FliG$_M$ dynamic correlation network*
MM S4, Related to Figure 6: *4FHR FliM$_M$ – FliG$_M$ inter-subunit coevolution network*

Movies

Movie S1, Related to Figure 3C: *4FHR PC1 bending motion (side view)*
Movie S2, Related to Figure 3C: *4FHR PC3 rotary motion (en-face view)*
Movie S3, Related to Figure 6A: *4FHR PEV deletion PC3 rotary motion (en-face view)*
Movie S4, Related to Figure 7D: *2HP7 rotary motion (en-face view). PC1 (first half) and PC average (second half)*
Movie S5, Related to Figure 7D: *3SOH rotary motion (en-face view). PC1 (first half) and PC average (second half)*
Movie S6, Related to Figure 7D: *4FQ0 rotary motion (en-face view). PC1 (first half) and PC average (second half)*
Movie S7, Related to Figure 8F: *3AJC rotary motion (en-face view). PC1 (first half) and PC average (second half)*
Movie S8, Related to Figure 8F: *3USW rotary motion (en-face view). PC1 (first half) and PC average (second half)*
